# A Proline Mimetic for the Design of New Stable Secondary Structures: Solvent-Dependent Amide Bond Isomerization of (*S*)-Indoline-2-carboxylic Acid Derivatives

**DOI:** 10.1021/acs.joc.1c00184

**Published:** 2021-06-03

**Authors:** Matteo Pollastrini, Filippo Lipparini, Luca Pasquinelli, Federica Balzano, Gloria Uccello Barretta, Gennaro Pescitelli, Gaetano Angelici

**Affiliations:** † Dipartimento di Chimica e Chimica Industriale, Università di Pisa, Via G. Moruzzi 13, 56124 Pisa, Italy

## Abstract

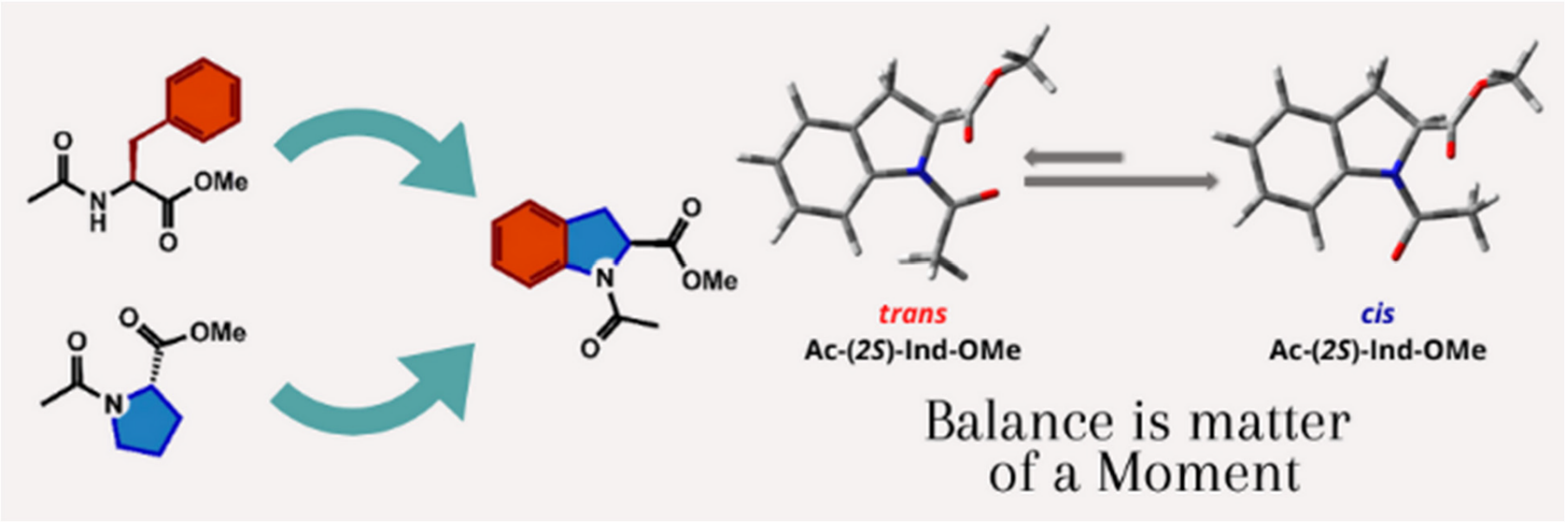

A thorough experimental and computational study on the conformational properties of (*S*)-indoline-2-carboxylic acid derivatives has been conducted. Methyl (*S*)-1-acetylindoline-2-carboxylate, both a mimetic of proline and phenylalanine, shows a remarkable tendency toward the *cis* amide isomer when dissolved in polar solvents. This behavior is opposite to the general preference of proline for the *trans* isomer, making indoline-2-carboxylic acid a good candidate for the design of different secondary structures and new materials.

## Introduction

Conformational diversity is Nature’s way of disclosing important properties; therefore, a deeper understanding of the intrinsic conformational order of natural and unnatural amino acids is essential for the rational design of biomimetic compounds. Proline and phenylalanine are two important amino acids often involved, respectively, in protein conformational switching and aggregation phenomena. For proline and its analogues, the pioneering work of Wennemers’ group showed that a deep knowledge of the conformational properties of model peptides in different environments,^1^ especially for the *cis*/*trans* isomerization equilibrium, can lead to a number of applications in catalysis^2^ or in the synthesis of collagene-like materials,^3−5^ as a tool for polyproline II crystal structure resolution^6^ or in the use of oligoprolines in supramolecular assembled materials.^7,8^ Regarding phenylalanine, since the groundbreaking studies by Gazit about the aggregation properties of amyloid peptides,^9,10^ so many developments have taken place in nanobiotechnology,^11,12^ and biomaterials^13^ that it seems appropriate to refer to more extensive reviews.^14−16^ The deeper comprehension and combination of the many factors that control the *cis*/*trans* isomerization and aggregation, like n→π* interactions,^17−24^ π–π stacking,^25,26^ or steric factors,^27−31^ have led to the study of many peptide analogues. For example, Tomasini’s group developed oxazolidinone-containing peptides,^32,33^ showing that the Phe-d-Oxd moiety is a privileged scaffold for controlling the formation of supramolecular materials,^34,35^ thanks to the preferential *trans* conformation of generic Xaa-d-Oxd bonds and the presence of phenylalanine. Moreover, they recently developed new soft materials based on l-DOPA,^36,37^ a psychoactive analogue of phenylalanine, and in collaboration with them we could spot through chiroptical techniques the elusive π-helix motif attained by oligomers containing pyroglutamic acid,^38^ a proline mimetic. (*S*)-Indoline-2-carboxylic acid ((2*S*)-Ind) is an interesting case, as it is both a mimetic of l-proline and l-phenylalanine. (2*S*)-Ind can be considered as the result of the fusion of an aromatic ring to the pyrrolidine bond between C4 and C5 of proline. At the same time, (2*S*)-Ind can also be seen as a phenylalanine with the side chain conformationally locked in a fixed orientation. Both these features make (2*S*)-Ind a good candidate for the investigation of its conformational properties. Contrary to the many studied proline analogues with substituents at position C4 and C5,^39−43^ pseudoprolines,^44^ or other bicyclic proline derivatives,^45,46^ (2*S*)-Ind cannot undergo stabilization or destabilization by ring puckering, as the 5-membered ring is intrinsically quasiplanar.

**Scheme 1:**
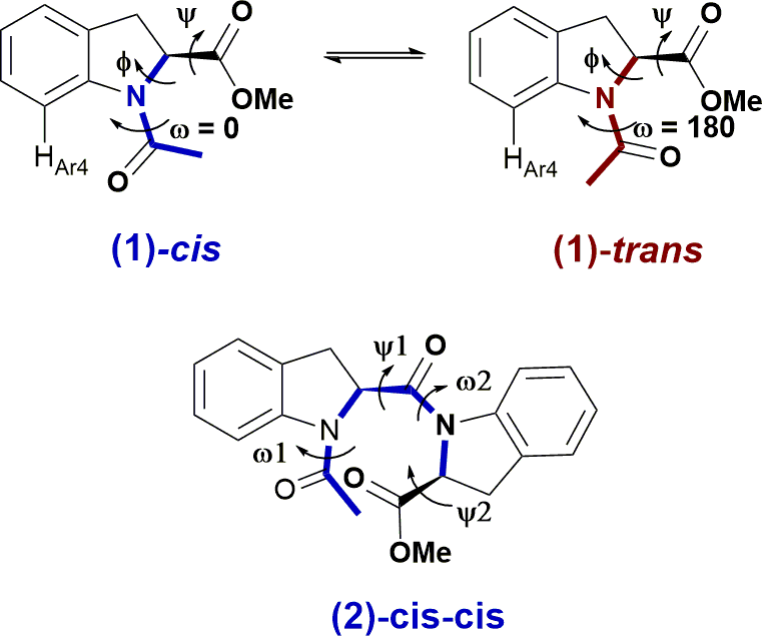
(Top) *Cis–Trans* Isomerization Equilibrium of the Amidic Bond in Ac-(2*S*)-Ind-OMe (**1**) and Major Torsional angles. (Bottom) Structure of Ac-(2*S*)-Ind-(2*S*)-Ind-OMe (**2**) Shown as the Major *Cis–Cis* Conformer, Relative to the Two ω Angles

The biological importance of kinetic and thermodynamic quantities of prolinamide conformational interconversions is well demonstrated, for example, by the extensive work of Fischer’s group.^47−51^*Cis*/*trans* proline isomerization has indeed often been found as the rate-limiting step in the protein-folding processes. Regarding specifically (2*S*)-Ind, an earlier explorative computational study of Torras et al.^52^ on the *N*-acetyl-*N*′-methylamide derivative (Ac-(2*S*)-Ind-NHMe) investigated the structural role of the aromatic ring, highlighting that its presence further reduces the intrinsically low conformational flexibility of proline, giving higher preference for the *cis* state of the peptide bond involving the pyrrolidine nitrogen. However, the presence of a terminal secondary amide might have a strong influence on the conformation of the molecule, given the possibility of hydrogen bond formation. Consequently, in the conformational analysis of proline and its analogues, acetylated methyl esters or *N*-acetyl-*N*′,*N*′-dimethylamides are often preferred.^53^ For this reason we decided to investigate Ac-(2*S*)-Ind-OMe (**1**) and the dimer Ac-(2*S*)-Ind-(2*S*)-Ind-OMe (**2**) (Scheme 1) to explore both the methyl ester derivative and the amidic bond in the dipeptide. Referring to Scheme 1, it was anticipated that in Ac-(2*S*)-Ind-OMe angle ϕ is fixed by the pyrrolidine ring; thus, the main degrees of conformational freedom of **1** are represented by the torsional angles ω and ψ. The amide angle ω is expected to assume values around 0° and 180° leading to *cis* and *trans* isomers, respectively, distinguished by NMR. Conversely, the rotational barrier for angle ψ is too low to be recognized by NMR, but it is still important for the overall conformational equilibrium.

## Results and Discussion

**Figure 1:**
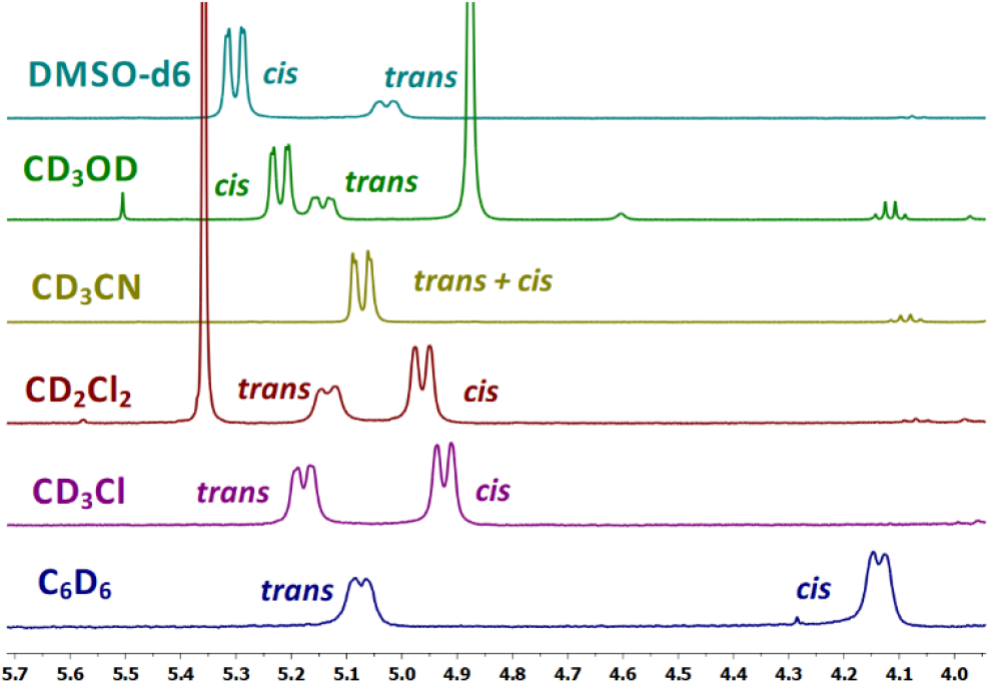
^1^H NMR in the Hα region of a 0.1 M solution of **1** in different solvents at 25 °C.

We were pleased to see that the ^1^H NMR in CDCl_3_ of **1** showed well-separated signals, and through a complete bidimensional analysis it was possible to unambiguously assign each signal of the *cis* and *trans* isomers; the procedure is detailed in the Experimental Section. The capability to distinguish each signal is useful to study the physicochemical parameters of the equilibrium in solution. It was possible to evaluate the populations of *cis* and *trans* isomers, and consequently, the value of the *cis*-to-*trans* equilibrium constant *K_trans/cis_* at 25 °C, through the ratio between the integrated areas of the two peaks corresponding to the Hα of the two isomers in the ^1^H NMR spectrum, found between 4.1 and 5.2 ppm (Figure 1 and Table 1). The preferred species in a 0.1 M CDCl_3_ solution of **1** is the *cis* conformer, with a corresponding *K_trans/cis_* = 0.87. The conformational preference seems to be independent of the concentration, as the *trans*/*cis* ratio did not change analyzing 0.04 and 0.8 M solutions of **1** in CDCl_3_. More interestingly, we observed a marked influence of the solvent polarity on the equilibrium (Figure 1 and Table 1). More polar solvents favor the prevalence of the *cis* conformer. Another interesting phenomenon, also evident in Figure 1, is that the signal of the Hα in the *cis* conformer moves significantly to lower frequencies depending on the polarity of the solvent, while the Hα signal corresponding to the *trans* conformer is less affected.

**Table 1:** *K_trans/cis_* of Ac-(2*S*)-Ind-OMe (**1**) Obtained from ^1^H NMR Spectroscopy of 0.1M Solutions in Different Deuterated Solvents at 298 K

solvent	*K*_trans/cis_	δ (ppm) *trans*^a^	δ (ppm) *cis*^a^	ε_r_^b^
C_6_D_6_	0.72	5.06	4.14	2.3
CDCl_3_	0.87	5.17	4.91	4.8
CD_2_Cl_2_	0.63	5.10	4.93	8.9
CD_3_OD	0.47	5.15	5.22	32.7
CD_3_CN	0.43^c^	5.04	5.04	37.5
DMSO-*d*_6_	0.31	5.02	5.30	46.7

a Chemical shift values of the Hα of the *trans* and *cis* conformer.

b Solvent dielectric constant.

c For CD_3_CN, *K*_*trans/cis*_ was calculated through the ratio between the integrated areas of the CH_3_ acetamide signals of the two isomers.

As it can be seen in Table 1, by testing several solvents, the *trans*/*cis* ratio spans from 0.72 in benzene-*d*_6_ to 0.31 in DMSO-*d*_6_, depending on the dielectric constant (ε_r_) of the solvent. This observation can be rationalized by assuming a larger dipole moment for the *cis* isomer than for the *trans* one, which results in a stabilization of the *cis* conformer by more polar solvents.^54^ The experimental *K_trans/cis_* values were fitted against an empirical correlation of the type defined by eq SI4 (see the Supporting Information, SI), which describes a solvent-dependent conformational equilibrium where the solvent is seen as a uniform dielectric with relative permittivity ε_r_.^55^ A very good fit was observed for all solvents, except benzene, demonstrating that the equilibrium is indeed controlled by the solvent reaction field, while benzene is possibly capable of specific interactions with the solute aromatic ring.

Comparing the measured *K_trans/cis_* with those reported for Ac-Pro-OMe, we can see that the nature of the solvent and the presence of the aromatic ring drastically affects the conformational equilibrium around the amide bond (Table 2). More importantly, contrary to most proline-mimetic compounds reported in the literature, **1** shows in solution an excess of the *cis*-amide isomer which increases with the solvent dielectric constant.

**Table 2:** Comparison of the *K*_trans/cis_ of Ac-(2*S*)-Ind-OMe (**1**) and Ac-Pro-OMe in Different Solvents at 298 K

Xaa	CDCl_3_	DMSO-*d*_6_	C_6_D_6_
Ac-Pro-OMe	3.6^53^	3.8^53^	∼4.9^56^
Ac-(2*S*)-Ind-OMe	0.87	0.31	0.72

To confirm the importance of solvent polarity in the stabilization of the *cis* conformer of **1**, we performed a series of titrations. We observed that adding sequential amounts of D_2_O to a DMSO-*d*_6_ solution of **1** did not affect at all the preference for the *cis* isomer, while adding portions of DMSO-*d*_6_ to a solution of **1** dissolved in benzene-*d*_6_ proportionally increased the population of the *cis* isomer. These experiments show that the conformational preference is dominated by the solvent polarity, as expected, rather than by solute–solvent hydrogen bonding.

**Figure 2:**
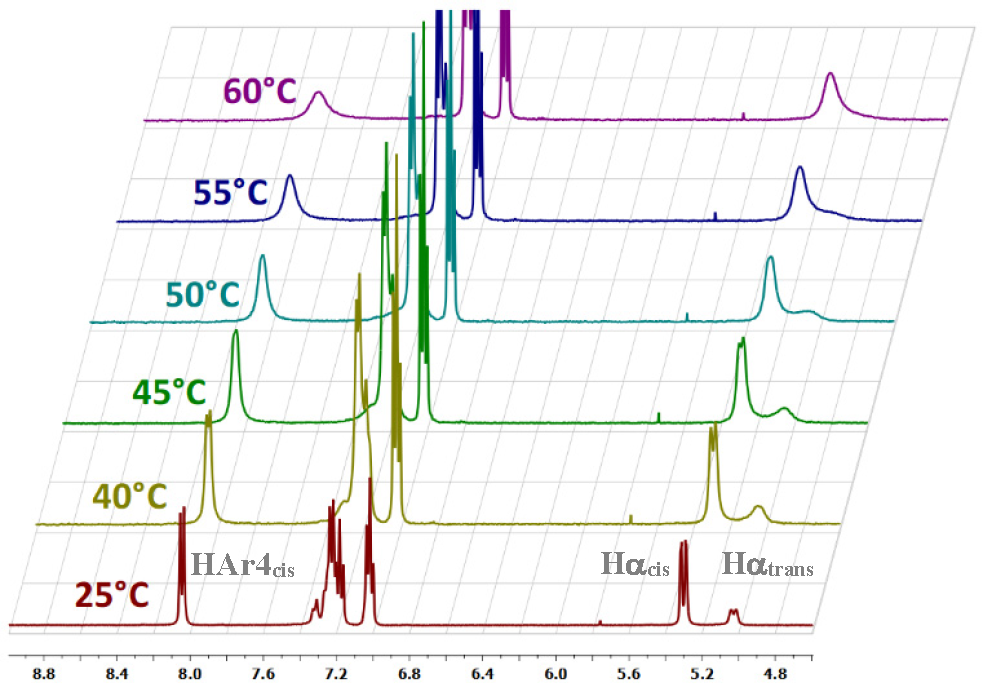
^1^H NMR of a 0.1 M solution of **1** in DMSO-*d*_6_ at different temperatures

With the aim of evaluating the thermodynamic parameters associated with the isomerization equilibrium in DMSO-*d*_6_, we performed variable-temperature NMR experiments to be analyzed by the Van’t Hoff equation.^57^ As can be seen in Figure 2, the increase of temperature led to a coalescence of the *cis/trans* Hα signals around 5.2 ppm already at 60 °C, indicating a fast exchange between the two populations. However, the characteristic signal of the aromatic proton H_Ar4*cis*_, deshielded by the proximate acetamide group when the amidic bond is in the *cis* conformation, remains anchored around 8.2 ppm, allowing the determination of *K*_eq_ at different temperatures. By plotting ln *K*_eq_ vs 1/*T*, Δ*H*° and Δ*S*° can be obtained (see the SI). The estimated values are Δ*H*° = +3.10 kcal/mol and Δ*S*° = +8.2 cal/molK for the *cis*-to-*trans* equilibrium (in DMSO). Thus, an enthalpy/entropy compensation effect seems to be at play for the considered conformational equilibrium.

Different methods have been proposed to measure the rate constants and the activation energy for the *cis/trans* isomerization.^58,59^ However, considering the unequal population between the two exchanging species, as well as similar spin–lattice relaxation time *T*_1_ of the two species (*T*_1*cis*_ = 2.39 s; *T*_1*trans*_ = 2.46 s) we found the approach outlined by Perrin and Dwyer to be convenient.^60^ 2D exchange spectroscopy (EXSY), which provides a map of the exchanging species, was used. This approach allows for the calculation of the rate constants for a chemical exchange process by measuring the cross-peak to diagonal peak intensity ratio. The ratio of the rate constants, obtained with the optimal mixing time found (τ_m_ = 0.03 s), was *K_trans/cis_* = *k*_*c*→*t*_/*k*_*t*→*c*_ = 0.31. This value is fully consistent with that obtained from the Hα peak integral ratio at 25 °C (see above). From the rate constants, we can also calculate the free energy of activation for the *cis* to *trans* and the *trans* to *cis* isomerization according to the absolute rate theory. The estimated values were Δ*G*^⧧^_c→t_ = 16.4 kcal/mol and Δ*G*^⧧^_t→c_ = 15.7 kcal/mol, which lie in the lower range of observed *cis/trans* barriers for *N,N*-disubstituted acetamides.^61^ NMR spectra of **1** acquired in CD_2_Cl_2_ to the lower temperature of −60 °C did not influence at all the conformational equilibrium.

A well-recognized factor, which favors the *trans* conformer over the *cis* conformer in proline analogues, is the n→π* interaction between adjacent carbonyl groups.^20,23^ The nature of the terminal group can affect the equilibrium, as esters are more electrophilic than amides and act as better acceptors for the n→π* interaction.^53^ In our case, given the *cis* preference of **1**, we speculated that other factors may overcome a possible n→π* interaction. Therefore, we expected that the presence of a tertiary amide as the acceptor group would not dramatically change the conformational preference. For that reason, we decided to synthesize compound **2** as model to confirm the preference of the *cis* conformer in an (*S*)-indoline-2-carboxylic acid derivative involved in a peptide bond (Scheme 1). Compound **2** is short enough to be a convenient model to investigate the impact of a tertiary amide in the backbone, avoiding the occurrence of cooperative effects which would show up in larger oligomers.

**Figure 3:**
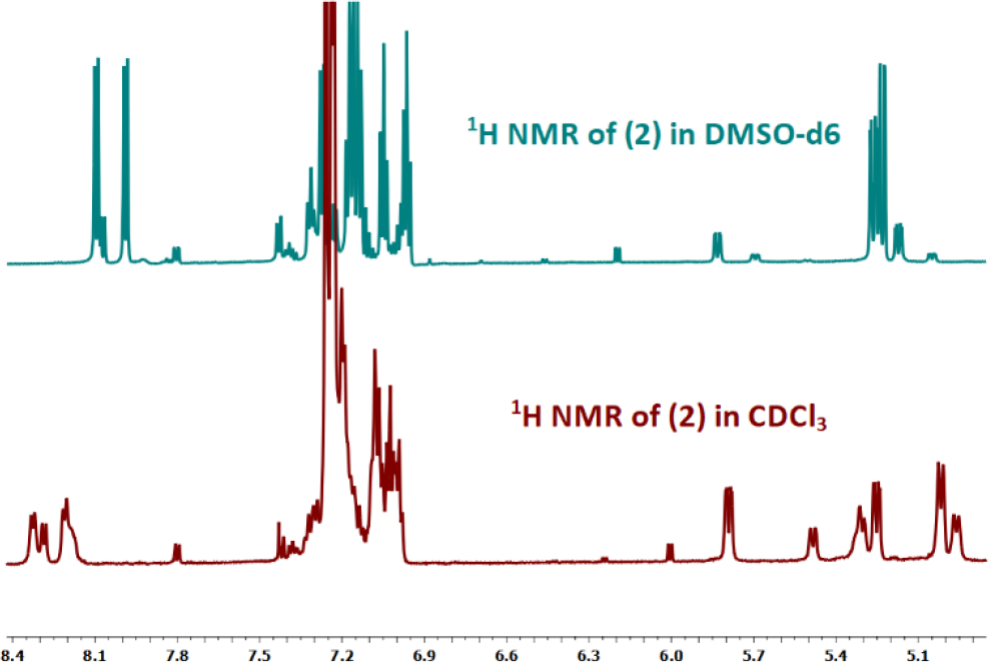
^1^H NMR of **2** in the Hα region and in the aromatic region in CDCl_3_ and DMSO-*d*_6_.

The NMR analysis in DMSO-*d*_6_ of the dimer Ac-(2*S*)-Ind-(2*S*)-Ind-OMe (**2**) demonstrated for both amidic bonds a strong preference for the *cis* conformation, confirming this tendency also when a tertiary amide moiety is present as terminal substituent. In Figure 3, it is possible to appreciate the influence of solvent polarity in CDCl_3_ and DMSO-*d*_6_, while a complete attribution of NMR signals of the predominant *cis–cis* conformer of **2** in DMSO-*d*_6_ is reported in the Experimental Section. Although compound **2** is too short to fold into a stable secondary structure, we expect the same preference for the *cis* geometry of the amide junction to be retained in longer oligomers of indoline-2-carboxylic acid, which will be the subject of future studies.

**Figure 4:**
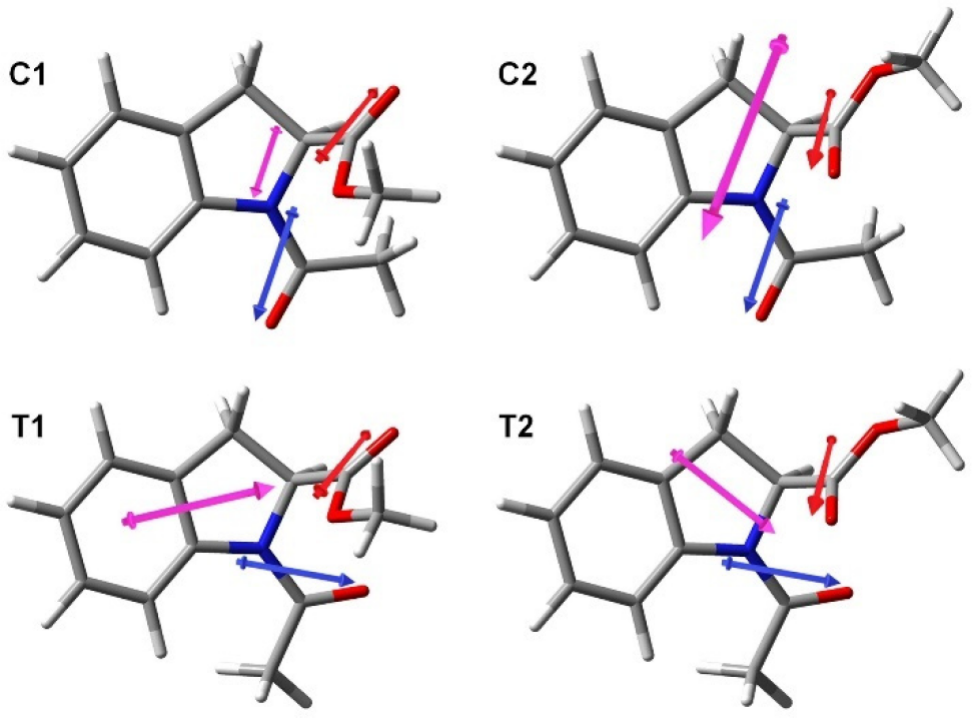
Optimized geometries of the four conformers of **1** in DMSO obtained using the B3LYP-D3BJ functional and the 6-311+G(d,p) basis set and the IEFPCM continuum solvation model. Arrows represent dipole moments: magenta, overall molecular dipole moment (the size is proportional to the magnitude); blue and red, local dipole moments associated to the amide and ester moiety, respectively, estimated at the same level of calculation on *N*,*N*-dimethylacetamide and methyl acetate.

To investigate the *cis/trans* isomerization from a different perspective, a computational study was performed to determine the relative stability of the two isomers of **1** in various solvents. Four different conformations (called C1, C2 and T1, T2, respectively) were considered, characterized by values of the ω dihedral of approximately 0 and 180 deg (C and T isomers), and of the ψ dihedral around +160 and −20 degrees (1 and 2 conformers), respectively. The optimized geometries of the C1, C2 and T1, T2 conformers in DMSO are reported in Figure 4.

For each starting geometry, a full optimization was performed, followed by a frequency calculation to characterize the optimized geometries as minima and compute, for each conformer, its Gibbs free energy. The computed Gibbs free energy differences between the various conformers in different solvents are reported in Table 3, while all of the optimized geometries and absolute Gibbs free energies are reported in the SI.

**Table 3:** Computed Relative Free Energies of the Four Conformers of Ac-(2*S*)-Ind-OMe, in kcal/mol

solvent	C1	C2	T1	T2	*K*_trans/cis_
benzene	0.0	0.1	0.4	0.4	0.55
CHCl_3_	0.0	0.0	0.4	0.3	0.52
CH_2_Cl_2_	0.1	0.0	0.5	0.4	0.53
CH_3_OH	0.3	0.0	0.6	0.5	0.48
CH_3_CN	0.3	0.0	0.6	0.5	0.48
DMSO	0.3	0.0	0.6	0.5	0.48

We note that, qualitatively, DFT calculations nicely reflect the experimental finding that the *cis* isomer is favored in all solvents, and there is a tendency toward the stabilization of the *cis* isomer in more polar solvents. Quantitatively, however, the impact on the conformational equilibrium of the different solvents is underestimated by calculations. From the computed dipole moments in various solvents of the *cis* and *trans* isomers, calculated as the Boltzmann average of the dipoles of the two conformers for each isomer, it appears that there is no definite prevalence of either isomer as the more polar one. This is due to the combination of the local dipoles allied with the amide and ester moieties, which in the four isomers arrange in different ways as depicted in Figure 4, yielding an overall dipole with variable direction and intensity. It is clear that, being the populations of the four conformers all very similar, and the respective dipole moments very variable, subtle changes in the populations may affect the average dipole moment substantially. This may be the reason why polarizable continuum solvent models seem not fully adequate to quantitatively describe the solvent-dependent conformational equilibrium of Ac-(2*S*)-Ind-OMe (**1**).

For the sake of completeness, we carried out geometry optimizations in various solvents also on compound **2** (see the SI). In the dimer, angles ω1, ω2, ψ1, and ψ2 (Scheme 1) are allowed to vary, yielding 16 potential conformers of which 15 corresponded to stable minima. In this case, too, we found the *cis–cis* conformers (i.e., with ω1 and ω2 ≈ 0°) to be the most stable ones, in good agreement with the experimental data.

**Figure 5:**
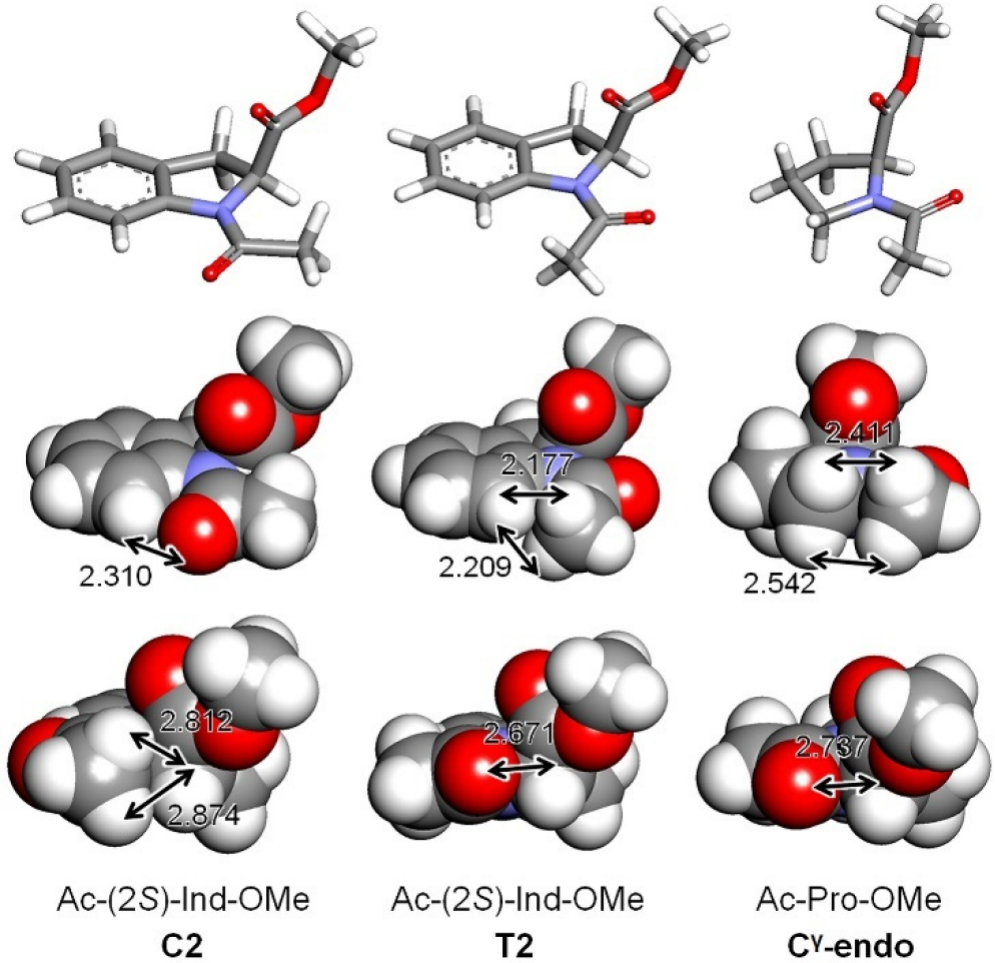
Analysis of selected van der Waals contacts (in Å) for structures optimized at B3LYP-D3BJ/6-311+G(d)/IEFPCM (DMSO) level of calculation, seen from two viewpoints. Conformers C2 and T2 of Ac-(2*S*)-Ind-OMe (**1**) were considered for better comparison with the lowest-energy conformer of Ac-Pro-OMe.

In the attempt to rationalize the observed behavior of (*S*)-indoline-2-carboxylic acid derivatives, we estimated all of the possible interactions that could influence their conformational preferences by using natural bond orbital (NBO) analysis. We first verified the extent of the n→π* interaction for compound **1**, in comparison with Ac-Pro-OMe which prefers the amide *trans* conformation.^17^ All the typical geometrical indicators of the n→π* interaction (C=O··COO distance, C=O–C Bürgi–Dunitz trajectory, pyramidalization of ester C),^20^ as well as the overlap between the n_C=O_ and π*_COO_ orbitals, were similar for the two compounds in their *trans* conformations (see the SI). This finding suggested that the different conformational behavior is not related to a different extent of the n→π* interaction. In fact, the main geometrical difference between Ac-(2*S*)-Ind-OMe (**1**) and Ac-Pro-OMe is not in the reciprocal arrangement between the amide and ester groups but in the fact that in the former compound the *N*-acetyl moiety lies in the same plane as the phenyl ring, as shown in Figure 5. A possible reason for the higher stability of the *cis* isomer of Ac-(2*S*)-Ind-OMe (**1**) is a subtle combination between steric and electrostatic effects involving the *N*-acetyl moiety. In the *trans* isomer of Ac-(2*S*)-Ind-OMe, a steric repulsion between CH_3_ and H_Ar4_ is detectable, which is relieved both in the *cis* isomer (between CH_3_ and CHα) and in Ac-Pro-OMe (between CH_3_ and CH_2_δ). Conversely, the *cis* isomer allows for a stabilizing attraction between C=O and H_Ar4_, although this latter effect is less important than anticipated. Atom charges calculated with natural population analysis reveal a slight increase of H_Ar4_ charge (by 10% in CHCl_3_) passing from the *trans* to the *cis* isomer of Ac-(2*S*)-Ind-OMe, which might be indicative of a C–H_Ar4_··O=C interaction in the *cis* isomer. At the same time, however, the atom charge on C=O oxygen is the same (within 0.5% in CHCl_3_) for the two isomers. Moreover, atom charges are only slightly affected when switching from CHCl_3_ to DMSO; for example, the C=O oxygen negative charge increases by ∼2% (absolute value) in the more polar solvent, while the H_Ar4_ charge increases by ∼1%. So, although the calculations hint at a possible C–H··O=C interaction, we must conclude that they do not highlight a clear solvent dependence for this interaction.

## Conclusions

The collected experimental and computational evidence unambiguously demonstrated that (*S*)-indoline-2-carboxylic acid derivatives, when involved in amide bonds, have a strong preference for the *cis* conformation, especially in polar solvents. It is interesting to note how these results confirm the findings of Siebler et al.^53^ on the importance of dipole moment for proline derivatives isomerization, although in our case the effect of the polarity of the solvent is much more pronounced. Besides the influence of the dipole moment on the conformational equilibrium, a combination of steric and electrostatic interactions may contribute to stabilizing the amide *cis* geometry. Considering that small differences at a molecular level transpose to big changes of macroscopic properties, these findings could lead to the synthesis of longer oligomers forming stable polyproline I like structures, or the use in peptide sequences of (*S*)-indoline-2-carboxylic acid as a preferential scaffold to form β-hairpins, or as a conformational constrain for the design of new organo-catalysts.

## Experimental Section

### General Information and Materials

All nonaqueous reactions were run in oven-dried glassware under a positive pressure of argon, with exclusion of moisture from reagents and glassware, transferring solvents and liquid reagents with hypodermic syringes. The glassware has been dried with a heating gun under vacuum and allowed to cool under argon. Anhydrous solvents and liquid reagents were obtained using standard drying techniques. Solid reagents were of commercially available grade, used without further purification and, when necessary, stored in a controlled atmosphere and/or at −20 °C. (*S*)-Indoline-2-carboxylic acid was purchased from abcr GmbH. Reactions were monitored by thin-layer chromatography using Merck silica gel 60 F254 plates. Visualization of the developed chromatogram was performed by UV absorbance, aqueous potassium permanganate, or iodine. Flash chromatography was performed using Sigma-Aldrich silica gel 60, particle size 40–63 μm, with the indicated solvent system. NMR spectra, unless otherwise specified, were recorded on Bruker Avance DRx 400, 401.36 MHz for ^1^H and 100.92 MHz for ^13^C. Chemical shifts are reported in ppm with the deuterated solvent signal as the internal standard. Data are reported as follows: chemical shift, integration, multiplicity (s = singlet, d = doublet, t = triplet, q = quartet, qn = quintet, m = multiplet and br = broad), and coupling constant in hertz. All ^13^C spectra were obtained with complete proton decoupling. Structural assignments were made with additional information from gCOSY, gHSQC, gHMBC, and NOESY experiments. 2D-EXSY (exchange spectroscopy) experiments, based on 2D-ROESY (rotating-frame Overhauser enhancement spectroscopy) sequence, and NOESY experiments were recorded on a VARIAN INOVA 600 MHz instrument. The optimized 2D map was recorded by using a relaxation time of 15 s, a mixing time of 0.03 s; 128 increments of 4 transients of 2K points each were collected. HPLC-ESI-Q/ToF flow injection analyses (FIA) were carried out with a 1200 Infinity HPLC (Agilent Technologies, USA), coupled with a quadrupole–time-of-flight tandem mass spectrometer (6530 Infinity Q-TOF; Agilent Technologies) through a Jet Stream ESI interface (Agilent). Mass Hunter Workstation Software (B.04.00) was used to control the HPLC and the mass spectrometer, for data acquisition, and for data analysis.

### Methyl (*S*)-1-Acetylindoline-2-carboxylate Ac-(2*S*)-Ind-OMe (**1**)

(*S*)-Indoline-2-carboxylic acid (H-Ind-OH) (2.8 g, 17.2 mmol) was dissolved in 280 mL of methanol and cooled to 0 °C with an ice bath. To this suspension was added dropwise thionyl chloride (1.87 mL, 25.7 mmol, 1.5 equiv). The reaction mixture was stirred for 1 h and allowed to reach room temperature. Afterward, the resulting solution was heated at 70 °C and allowed to stir at reflux for 16 h. After being cooled at room temperature, the reaction mixture was concentrated under reduced pressure. The residue was dissolved in ethyl acetate (EtOAc) and washed with an aqueous saturated solution of sodium bicarbonate (NaHCO_3_) three times. The combined organic layers were washed with brine, dried over Na_2_SO_4_, and concentrated under reduced pressure. The resulting crude material was purified by flash chromatography on silica gel using 10 to 50% EtOAc in hexane to give the desired product (H-(2*S*)-Ind-OMe) as a white solid in an 82% yield (2.5 g, 14.1 mmol). ^1^H NMR (400 MHz, CDCl_3_) δ = 7.12–6.99 (m, 2H), 6.79–6.68 (m, 2H), 4.39 (dd, *J* = 10.2, 5.5 Hz, 2H), 3.76 (s, 3H), 3.36 (m, 2H).

In a round-bottom flask, 510 mg (2.9 mmol, 1 equiv) of H-(2*S*)-Ind-OMe was dissolved in 25 mL of dry dichloromethane and the resulting solution stirred at 0 °C. A catalytic amount (0.5%) of 4-dimethylaminopyridine (DMAP) and 1.60 mL (11.5 mmol, 4 equiv) of triethylamine (TEA) were added to the solution. Acetic anhydride (2.2 mL, 23 mmol, 8 equiv) was then added dropwise, and after 15 min, the reaction mixture was warmed at room temperature and let stirring for 16 h. The reaction mixture was then concentrated under reduced pressure, and the residue was dissolved in dichloromethane, acidified with HCl 1 M, and washed three times with dichloromethane. The combined organic layer layers were dried over Na_2_SO_4_, filtered, and concentrated under reduced pressure. The resulting crude product was purified by flash chromatography on silica gel using 10 to 50% EtOAc in hexane to give the desired product as a white solid in an 85% yield (538 mg, 2.45 mmol). TLC *R*_*f*_: 0.6 (Hex/EtOAc = 7:3), Mp: 65 °C. ^1^H NMR (400 MHz, CDCl_3_) δ = 8.21 (d, *J* = 8.1 Hz, 1H_cis_), 7.28–7.12 (m, 2H_cis_ + 3H_trans_), 7.03 (t, *J* = 7.7 Hz, 1H_cis_ + 1H_trans_), 5.17 (d, *J* = 10.7 Hz, 1H_trans_), 4.91 (d, *J* = 10.7 Hz, 1H_cis_), 3.77 (s, 3H_cis_), 3.73 (s, 3H_trans_), 3.62 (dd, *J* = 16.6, *J* = 10.7 Hz, 1H_cis_), 3.47 (dd, *J* = 16.6 Hz, *J* = 10.7 Hz, 1H_trans_), 3.27 (d, *J* = 16.6 Hz, 1H_cis_), 3.10 (d, *J* = 16.6 Hz, 1H_trans_), 2.48 (s, 3H_trans_), 2.17 (s, 3H_cis_). ^13^C{^1^H} NMR (100 MHz, CDCl_3_) δ: 171.8, 168.9, 142.6, 130.9, 128.4, 128.0, 127.9, 125.7, 124.2, 124.0, 123.5, 117.4, 113.8, 61.4, 60.6, 53.0, 52.5, 33.6, 31.5, 29.7, 24.5, 23.7. HRMS (TOF MS ES+): *m*/*z* [M + Na]^+^ calcd for C_12_H_13_NO_3_ 242.0788; found 242.0784. Analytical HPLC purity 97%.

### Methyl (*S*)-1-((*S*)-1-Acetylindoline-2-carbonyl)indoline-2-carboxylate, Ac-((2*S*)-Ind)_2_-OMe (**2**)

In a round-bottom flask, (*S*)-indoline-2-carboxylic acid (H-(2*S*)-Ind-OH) (0.6 g, 3.6 mmol, 1 equiv) was dissolved in 25 mL of dry dichloromethane and allowed to stir and cool in an ice bath. Triethylamine (TEA) (2.0 mL, 14.4 mmol, 4 equiv) and a catalytic amount (0.5%) of DMAP were added. To this solution, 2.7 mL of acetic anhydride (28.8 mmol, 8 equiv) was then added dropwise. The reaction mixture was warmed at room temperature and stirred for 16 h. The resulting mixture was then concentrated under reduced pressure, and the residue was dissolved in dichloromethane, acidified with HCl 1 M, and washed three times with dichloromethane. The combined organic layer layers were dried over Na_2_SO_4_, filtered, and concentrated under reduced pressure. The product Ac-(2*S*)-Ind-OH was obtained as a white solid with a yield of 85% (0.63g) and used without further purification. ^1^H NMR (400 MHz, DMSO-*d*_6_) δ = 8.20 (d, *J* = 8.0 Hz, 0.75H), 7.30–6.95 (m, 3.25H), 5.21 (d, *J* = 9.0 Hz, 0.75 H) and 4.87 (d, *J* = 9.0 Hz, 0.25H), 3.67–3.20 (m, 2H), 2.52 (s, 2.25H) and 2.19 (s, 0.75H).

In a two-neck round-bottom flask, 210 mg (1.02 mmol, 1 equiv) of Ac-(2*S*)-Ind-OH was dissolved in dry dichloromethane under argon. The coupling reagent 2-chloro-1-methylpyridinium iodide (Mukaiyama’s reagent) (365 mg, 1.43 mmol, 1.4 equiv) and freshly distilled TEA (0.4 mL, 2.86 mmol, 2.8 equiv) were added into the solution. The amine H-(2*S*)-Ind-OMe (181 mg, 1.02 mmol, 1 equiv) was added, and the reaction mixture was heated at reflux for 16 h before being allowed to cool to room temperature. The reaction mixture was then diluted with DCM and washed with HCl 1M, aqueous saturated sodium bicarbonate, and brine. The organic layer was then dried over Na_2_SO_4_ and evaporated under reduced pressure. The resulting solid was purified by flash chromatography on silica gel using 10–50% EtOAc in hexane. The desired product was obtained as white solid in an isolated yield of 46% for solubility issues (168 mg, 0.46 mmol). TLC Rf = 0.6 (DCM:MeOH = 9:1), M.P. = 175 °C (dec)^1^H NMR (400 MHz, CDCl_3_) δ = 8.35–8.14 (m, 1H), 7.36–7.11 (m, 5H), 7.11–6.95 (m, 2H), 5.87–5.75, 5.51–5.45, 5.34–5.21 and 5.12–4.92 (four m, 2H), 3.95–3.00 (m, 7H), 2.47 and 2.18 (couple of d, 3H). ^1^H NMR characterization of prevailing cis–cis species (>70%) in the stereoisomers mixture in DMSO-*d*_6_ on a 600 MHz is reported in the SI. ^13^C{^1^H} NMR (150 MHz, DMSO-*d*_6_) δ = 171.7, 169.7, 169.2, 143.5, 142.2, 129.5, 128.9, 127.4, 127.2, 124.7, 124.3, 123.1, 116.0, 115.9, 115.8, 113.8, 61.2, 61.0, 60.0, 59.7, 59.6, 53.1, 52.1, 33.4, 33.0, 32.9, 30.5, 23.6, 23.5, 20.7, 14.1. HRMS (TOF MS ES+): *m*/*z* [M + Na]^+^ calcd for C_12_H_20_N_2_O_4_ 387.1315; found 387.1317. Analytical HPLC purity 98%.

### Comment over the Stability of Indoline-2-carboxylic Acid Derivatives

The starting material (*S*)-indoline-2-carboxylic acid (H-(2*S*)-Ind-OH) has been purchased by abcr GmBh with a declared purity up to 95%. In our opinion, the risk of degradation of the carboxylic moiety is low, but on the other hand, the risk of oxidation to form 1*H*-indole-2-carboxylic acid could have been higher, and we checked regularly, over a long period of time (more than one year), the purity of the SM and of the synthesized derivatives by ^1^H NMR. We could observe that if the SM and H-Ind-OMe were not correctly stored under argon at 0 °C they could change color to a reddish powder and show the formation of a small signal on the ^1^H NMR typical of the indole. However, we were pleased to notice then whenever the secondary amine was protected as an acetamide or involved in a peptide bond, compounds remain stable and pure even after 2 years.

### Assignment of ^1^H NMR Signals of **1**

*Cis* and *trans* stereoisomers of **1** were attributed on the basis of NOESY analysis (see the SI). Even though the majority of dipolar interactions suffered from exchange processes occurring between the two species, one effect was selective for the trans one. In particular, in CDCl_3_ Hα at 5.17 ppm produced NOE at 2.17 ppm (acetyl group), which is due to exchange processes, but not at 2.48 ppm, which is its acetyl group. On the contrary, Hα at 4.91 ppm gave NOEs at both frequencies of acetyl groups. Therefore, Hα centered at 5.17 and 4.91 ppm must be attributed to *trans* and *cis* isomer, respectively. Starting from the frequency of Hα and based on the analysis of scalar correlations and integration, the complete assignment of *cis* and *trans* isomer was obtained (see the SI).

### Optimization of the Mixing Time for EXSY Experiments

The rate constants and the activation energy for the *cis*/*trans* isomerization were determined in DMSO-*d*_6_ from 2D exchange spectroscopy (EXSY) which provides a map of the exchanging species. Considering the greatly unequal populations of the two exchanging species, as well as similar spin–lattice relaxation time *T*_1_ of the two species (*T*_1*cis*_ = 2.39 s; *T*_1*trans*_ = 2.46 s) we found the approach outlined by Perrin and Dwyer convenient. This approach allows for calculation of rate constant for chemical exchange by knowing the diagonal peak to cross-peak intensity ratio. For a simple two-site exchange, the total exchange rate *k* (*k* = *k*_*t*→*c*_ + *k*_*c*→*t*_) is given by the equation
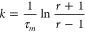
The term *r* accounts for unequal populations and is defined as
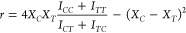
where *I*_*CC*_ and *I*_*TT*_ are the diagonal peak intensities of two exchangeable resonances in the EXSY, *I*_*TC*_ and *I*_*CT*_ are the intensities of the exchange cross peaks, and *τ*_*m*_ is the mixing time. *X*_*C*_ and *X*_*T*_ are the mole fractions of the *cis* and *trans* forms. The choice of the mixing time *τ*_*m*_ is critical. Kinetic effects on the cross-peak intensities will be too small to measure accurately, if it is too short. However, the effects will be so large as to be insensitive to the kinetic parameters, if it is too long. The optimum mixing time should be chosen to minimize the error in the rate constant and an approximate expression was shown to be
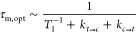
We found that τ_m_ = 0.03 s is the closest to the optimal value.

### ^1^H NMR Characterization of Prevailing *Cis–Cis* Species (>70%) of **2** in DMSO-*d*_6_ on a 600 MHz Instrument

The main species of Ac-Ind-Ind-OMe (**2**) in DMSO-*d*_6_ was fully characterized by compared analysis of homonuclear and heteronuclear scalar correlations in COSY and HSQC maps and homonuclear dipolar correlations in ROESY map (see the SI). The prevailing conformer was identified as the *cis*-*cis* one on the basis of the significant ROE detected between the more intense singlets at 2.05 and 3.73 ppm due to the methyl protons of the acetyl and the ester functions, respectively. Therefore, among NMR signals of Hα of the conformer mixture (5.00–6.00 ppm), the two more intense ones (doublet of doublets) at 5.24 and 5.27 ppm were assigned to the two units of the *cis*–*cis* species. In particular, the high-frequency signal (5.27 ppm) was attributed to the Hα of the residue with the *N*-acetyl terminal substituent, which is in spatial proximity of the methyl group of acetyl moiety (ROE constraint). Accordingly, to the above said stereochemical assignment, no ROEs between aromatic protons and Hα of methyl ester terminal residue were detected. Starting from each Hα, the two diastereotopic protons of their adjacent methylenes were assigned (3.29/3.78 ppm for the *N*-acetyl residue and 3.34/3.76 ppm for the methyl ester residue) by means of their scalar correlations. Har_1_ of each residue was identified on the basis of the ROE effects produced by the methylene protons. Resonances of aromatic protons of each residue were assigned on the basis of their scalar correlations, starting from Har_1_. NMR characterization data for the *cis*–*cis* species are collected in the SI.

### Computational Details

Conformational analysis was run by the systematic conformational search algorithm implemented in Spartan’18 (Wavefunction, Inc., Irvine, CA, 2018) using the Merck Molecular Force Field (MMFF). Geometry optimizations were performed using density functional theory with the B3LYP exchange-correlation functional,^62^ augmented with Grimme’s GD3 empirical dispersion correction dispersion,^63^ in conjunction with the 6-311+G(d) basis set. Solvation effects have been accounted for using the integral equation formalism^64,65^ formulation of the polarizable continuum model (IEF-PCM).^66^ Natural bond orbital (NBO) analysis was run with NBO version 3.^67^ All of the DFT calculations have been performed using the Gaussian 16 suite of programs.^68^

### HPLC Analysis of **1** and **2**

A 1000 ppm solution was prepared in DMSO and then further diluted in MeOH to ca. 50 ppm and injected in the chromatographic system. The separation was performed on an Agilent Zorbax Extend C18 Rapid resolution HT column (50 × 2.1 mm, 1.8 μm particle size). The injection volume was 1 μL, and the column temperature was 30 °C. Separation was obtained by using a gradient of 0.1% formic acid in water (eluent A) and 0.1% formic acid in acetonitrile (eluent B) programmed as follows: 90% A for 2 min, followed by a linear gradient to 50% B in 9 min, then to 70% B in 3.3 min, finally to 90% B in 3.7 min held for 12 min at 90% B. Re-equilibration time for each analysis was 13 min. The chromatographic runs were performed at a flow rate of 0.2 mL/min. The eluents were all HPLC-MS grade, Sigma-Aldrich. The MS acquisition was performed in full scan, and the Jet Stream ESI operating conditions were: drying gas (N_2_, purity >98%): 350 °C at 10 L/min; capillary voltage 4.5 kV; nebulizer gas 35 psig; sheath gas (N_2_, purity >98%): 375 °C at 11 L/min. High-resolution mass spectra were acquired in the range 100–3200 *m*/*z* in high-resolution positive mode. The fragmentor was kept at 175 V, nozzle voltage 1000 V, skimmer 65 V, octapole RF 750 V. The mass axis was calibrated prior analyses using the Agilent tuning mix HP0321 (Agilent Technologies) prepared in acetonitrile and water.

## Supporting Information Available

Full characterization of the synthesized compounds, NMR experiments, and calculation details (PDF)
FAIR data, including the primary NMR FID files, for compounds **1** and **2** (ZIP)
