# Supplementary material for: A Proline Mimetic for the Design of New Stable Secondary Structures: Solvent-Dependent Amide Bond Isomerization of (S)-Indoline-2-carboxylic Acid Derivatives
Source: J Org Chem. 2021 Jun 3;86(12):7946–54. doi: 10.1021/acs.joc.1c00184 (PMC8456495; doi:10.1021/acs.joc.1c00184)
Supplement: Supplementary file 1 — jo1c00184_si_001.pdf [file jo1c00184_si_001.pdf]

SUPPORTING INFORMATION

A proline mimetic for the design of new stable secondary structures: solvent dependent amide-bond isomerization of (*S*)-indoline-2-carboxylic acid derivatives

Matteo Pollastrini,[a] Filippo Lipparini,[a] Luca Pasquinelli,[a] Federica Balzano, [a] Gloria Uccello Barretta, [a] Gennaro Pescitelli [a] and Gaetano Angelici*[a]

[a]
Dipartimento di Chimica e Chimica Industriale
Università di Pisa
Via Giuseppe Moruzzi, 13 - 56124 Pisa
E-mail: gaetano.angelici@unipi.it

List of contents

1. ^{1}H NMR spectrum of Ac-(2*S*)-Ind-OMe (1) in $CDCl_3$ (0.1 M) at 400 MHz 3

2. ^{13}C{^{1}H} NMR spectrum of Ac-(2*S*)-Ind-OMe (1) in $CDCl_3$ at 100 MHz 4

3. COSY Correlations of Ac-(2*S*)-Ind-OMe (1) in $CDCl_3$ at 400 MHz 5

4. HSQC Correlations of Ac-(2*S*)-Ind-OMe (1) in $CDCl_3$ at 400 MHz 6

5. HMBC Correlations of Ac-(2*S*)-Ind-OMe (1) in $CDCl_3$ at 400 MHz 7

6. NOESY Correlations of Ac-(2S)-Ind-OMe (1) in $CDCl_3$ at 600 MHz........ 8

7. NOESY Correlations of Ac-(2S)-Ind-OMe (1) in DMSO-d6 at 600 MHz........ 9

8. ^{1}H NMR spectrum of Ac-(2*S*)-Ind-OMe (1) in benzene-d6 (0.1 M) at 400 MHz 10

9. ^{1}H NMR spectrum of Ac-(2*S*)-Ind-OMe (1) in CD_2Cl_2 (0.1 M) at 400 MHz 11

10. ^{1}H NMR spectrum of Ac-(2*S*)-Ind-OMe (1) in CD_3CN (0.1 mM) at 400 MHz 12

11. ^{1}H NMR spectrum of Ac-(2*S*)-Ind-OMe (1) in CD_3OD (0.1 M) at 400 MHz 13

12. ^{1}H NMR spectrum of Ac-(2*S*)-Ind-OMe (1) in DMSO-d6 (0.1 M) at 400 MHz 14

13. Variation of Ktrans/cis depending on the dielectric constants of the solvents 15

14. ^{1}H NMR of Ac-(2*S*)-Ind-OH in Acetic acid-d_4 at 400 MHz 16

15. ^{1}H NMR of Ac-(2*S*)-Ind-OH in DMSO-d6 at 400 MHz 17

16. ^{1}H NMR spectrum of Ac-(2*S*)-Ind-OMe (1) in DMSO-d6 (0.1 M) at different temperatures at 400 MHz.. 18

17. ^{1}H NMR spectrum of Ac-(2*S*)-Ind-OMe (1) in CD_2Cl_2 (0.1 M) at different temperatures at 400MHz 19

18. Titration of a 0.1 M solution of Ac-(2S)-Ind-OMe (1) in benzene-d6 (13.9 mg in 600 μL) with DMSO-d6 at 400 MHz. Variation of $K_{trans/cis}$ observed integrating the area of the two isomeric Hα. 20

19. 2D EXSY map of Ac-(2*S*)-Ind-OMe (1) in DMSO-d6 (0.1 M) at 600 MHz 21

20. ^{1}H NMR spectrum of Ac-(2*S*)-Ind-OMe (1) in $CDCl_3$ (0.04 M) at 400 MHz 22

21. ^{1}H NMR spectrum of Ac-(2*S*)-Ind-OMe (1) in $CDCl_3$ (0.8 M) at 400 MHz 23

22. ^{1}H NMR of Ac-(2*S*)-Ind-(2*S*)-Ind-OMe (2) in $CDCl_3$ (0.1 M) at 600 MHz 24

23. ^{1}H NMR of Ac-(2*S*)-Ind-(2*S*)-Ind-OMe (2) in DMSO-d6 (0.1 M) at 600 MHz 25

24. $^{13}C\{^1H\}$ NMR of Ac-(2*S*)-Ind-(2*S*)-Ind-OMe (2) in DMSO-d6 at 150 MHz 26

25. ^1H NMR signals assignment for cis-cis conformer of Ac-Ind-Ind-OMe (2) in DMSO-d6 on the basis of homonuclear scalar and dipolar correlations detected in COSY and ROESY maps.. 27

26. ROESY Maps of (2) in DMSO-d6.. 28

27. ^{1} HNMR characterization of the *cis-cis* species of (2) 29

28. HPLC-MS analysis of compound (1).. 30

29. HPLC-MS analysis of compound (2).. 32

30. Correlation between cis→trans equilibrium and solvent polarity 34

31. Representative van't Hoff plot based on the cis/trans equilibrium 35

32. Computed absolute free energies (in atomic units) of the various conformers of Ac-(2S)-Ind-OMe (1) in various solvents. 36

33. Computed dipole moments of the Cis and Trans isomers of Ac-(2S)-Ind-OMe, in kcal/mol. 36

34. Analysis of n-π* interaction.. 37

35. Computed relative free energies (in kcal/mol) of the various conformers of Ac-(2*S*)-Ind-(2*S*)-Ind-OMe in chloroform and DMSO.. 62

36. Geometries, computed SCF and free energies of all the optimized structures of the conformers of Ac-(2*S*)-Ind-(2*S*)-Ind-OMe in various solvents. 62

1. ^{1}H NMR spectrum of Ac-(2*S*)-Ind-OMe (1) in $CDCl_3$ (0.1 M) at 400 MHz

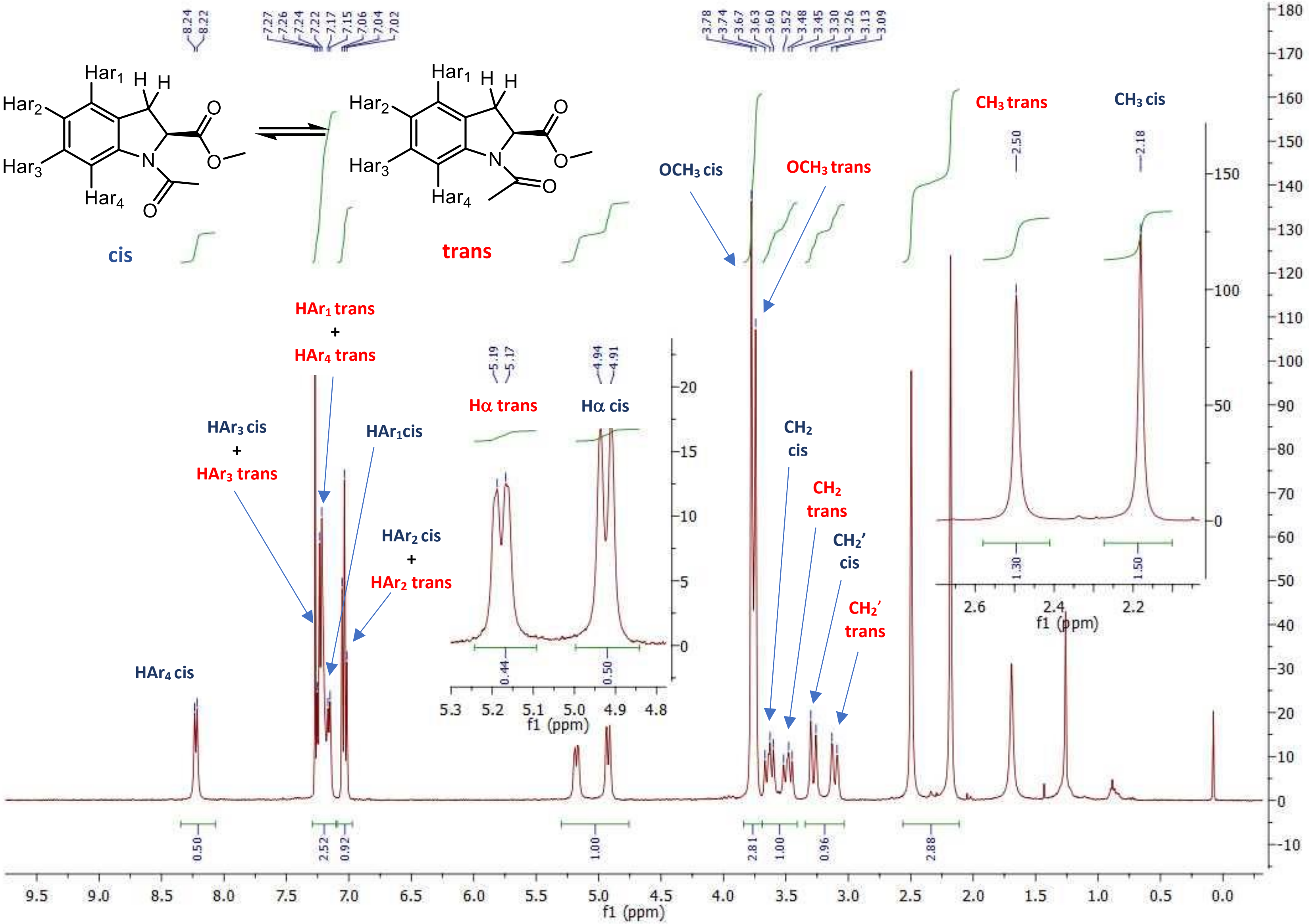

2. $^{13}C\{^1H\}$ NMR spectrum of Ac-(2*S*)-Ind-OMe (1) in $CDCl_3$ at 100 MHz

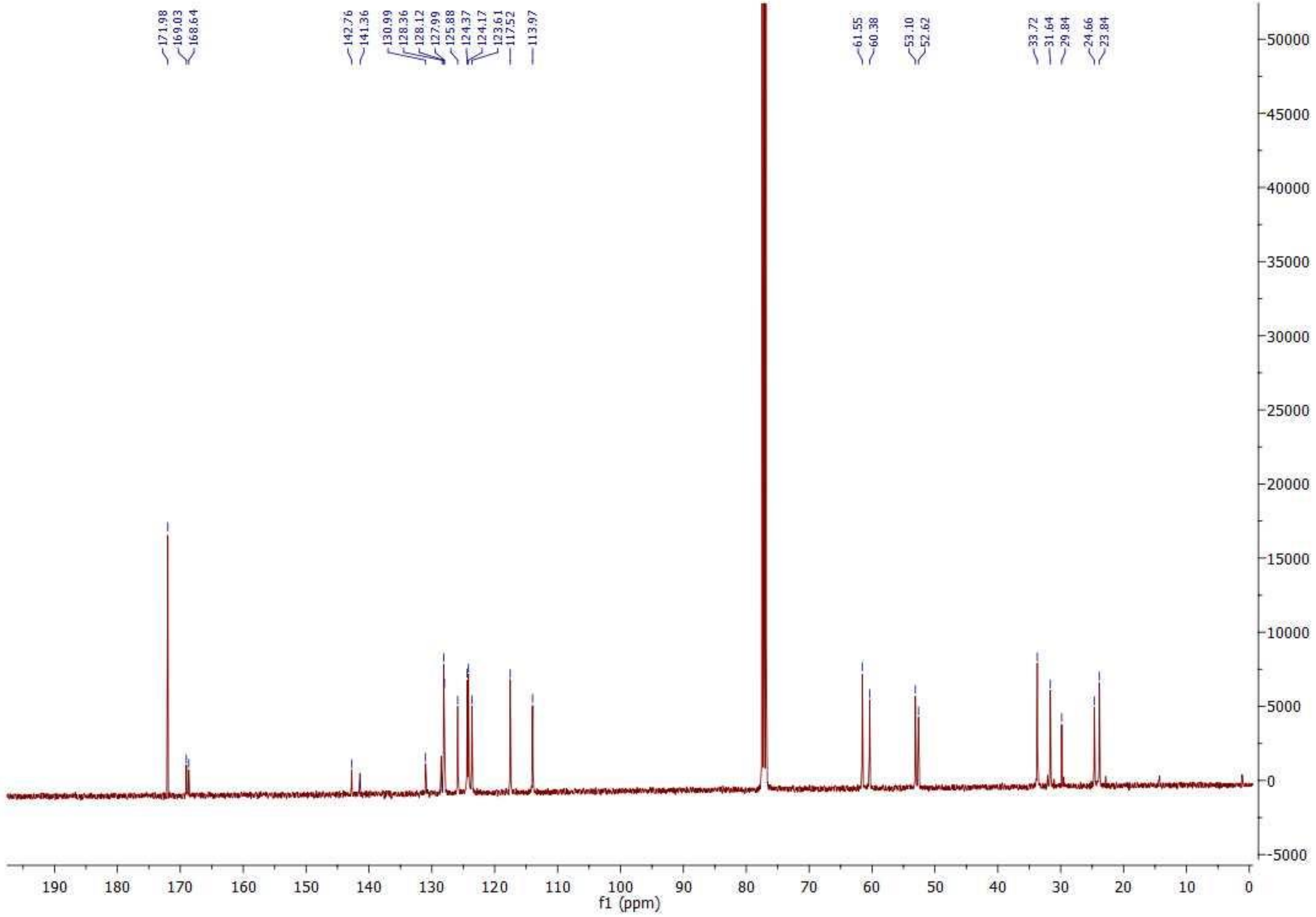

3. COSY Correlations of Ac-(2*S*)-Ind-OMe (1) in $CDCl_3$ at 400 MHz

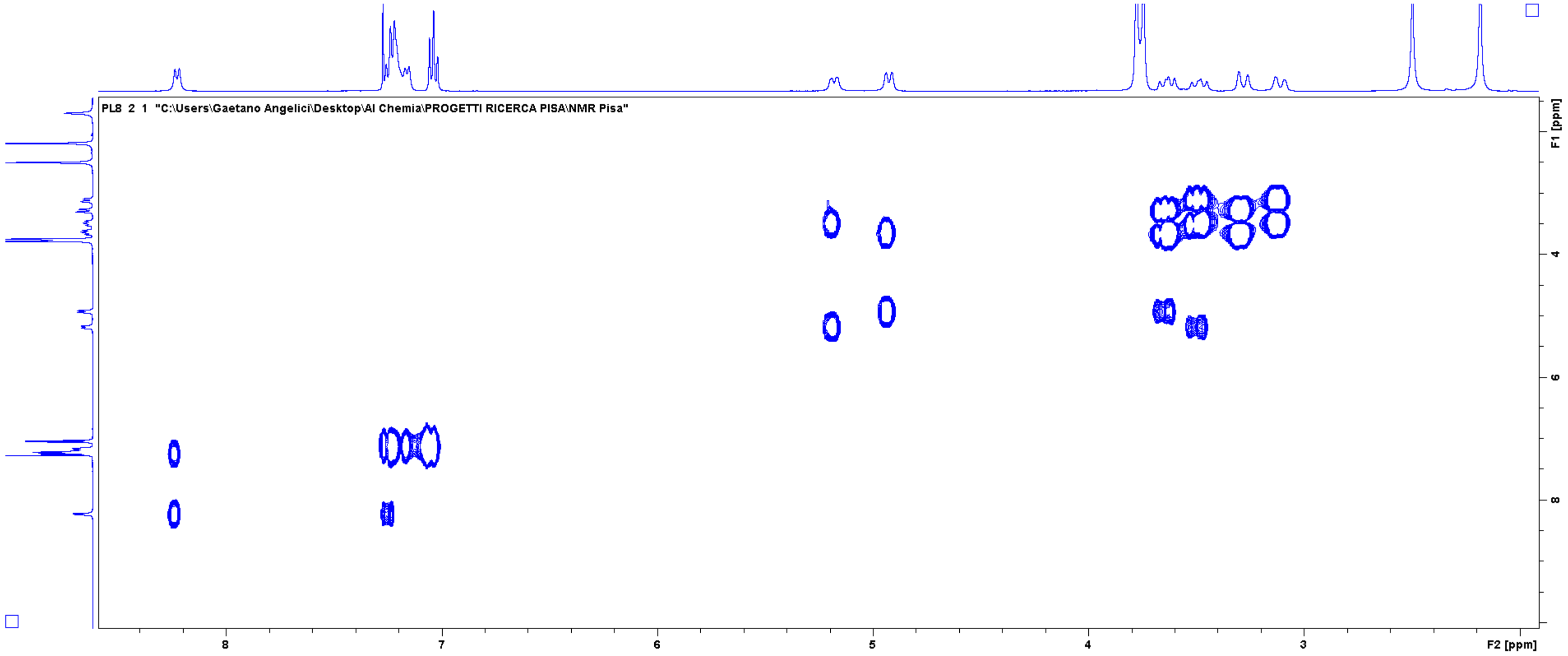

4. HSQC Correlations of Ac-(2*S*)-Ind-OMe (1) in $CDCl_3$ at 400 MHz

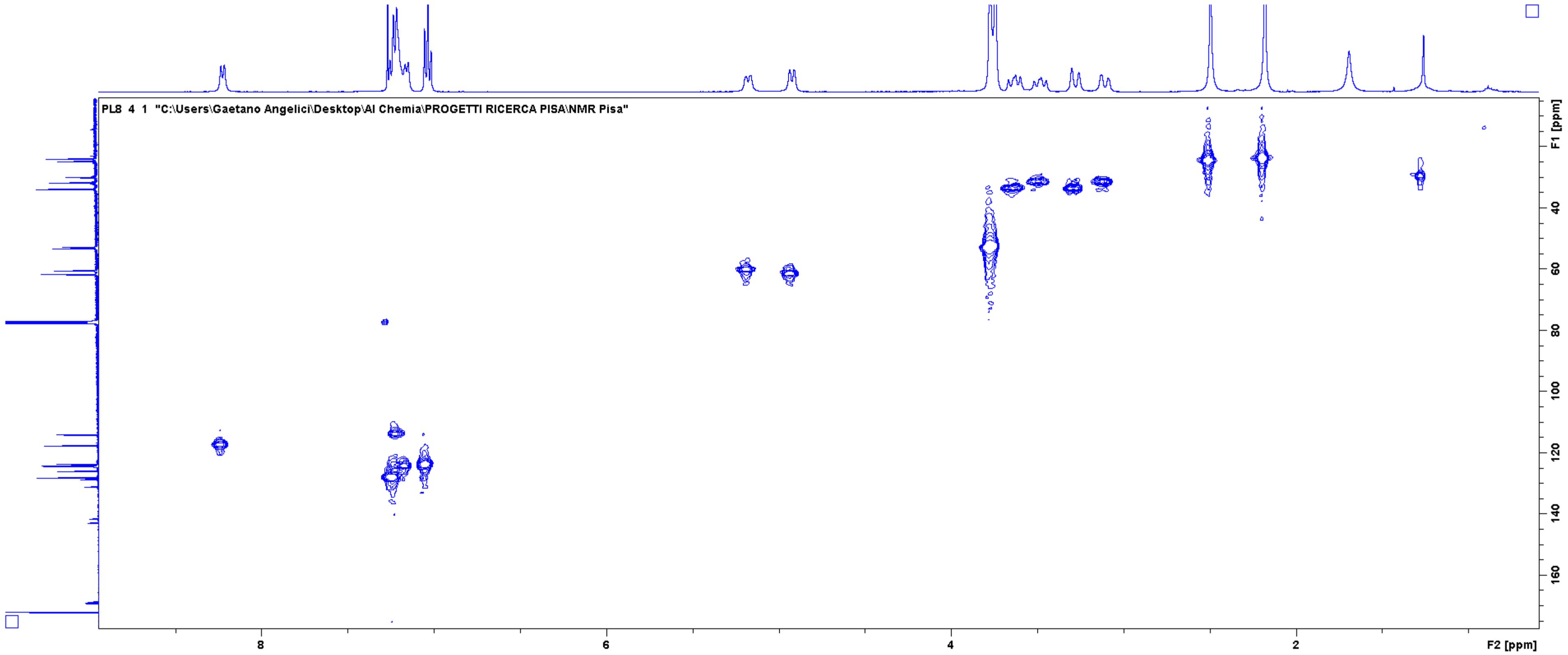

5. HMBC Correlations of Ac-(2*S*)-Ind-OMe (1) in $CDCl_3$ at 400 MHz

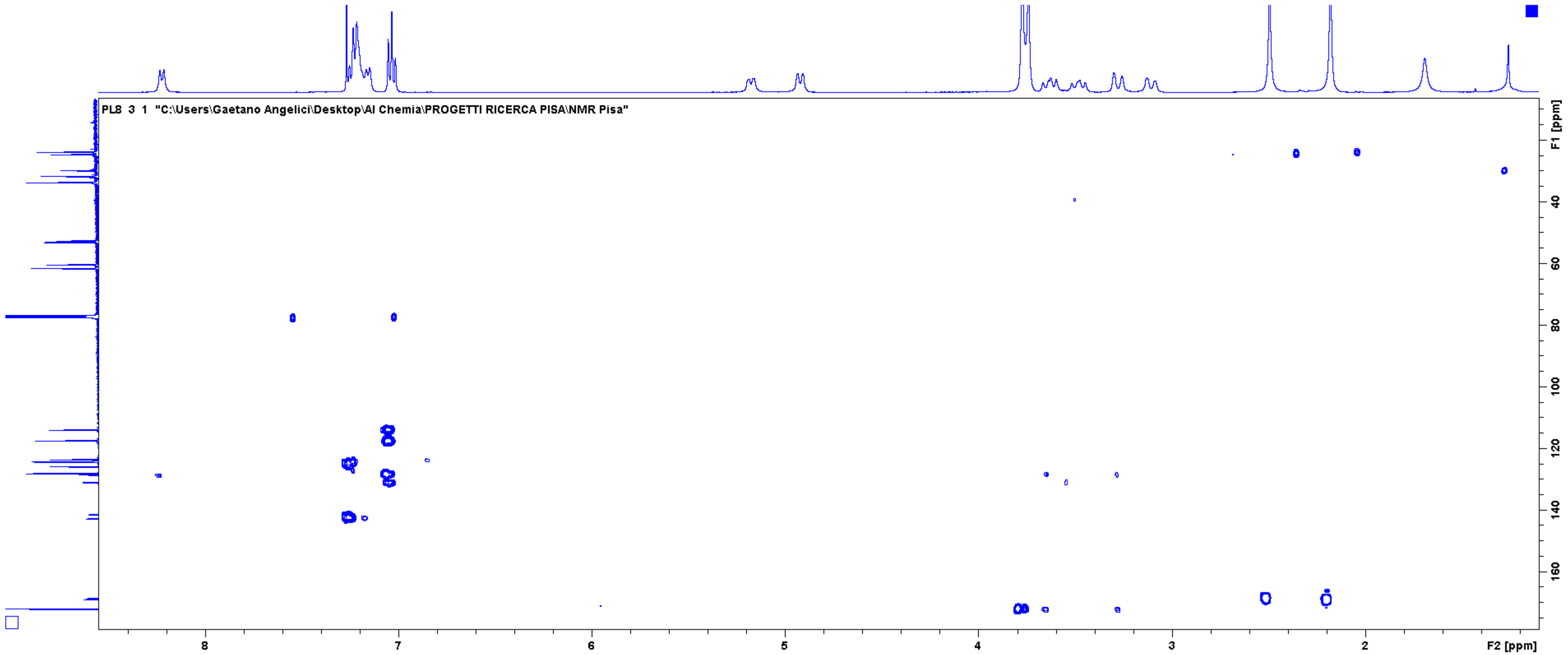

6. NOESY Correlations of Ac-(2S)-Ind-OMe (1) in $CDCl_3$ at 600 MHz

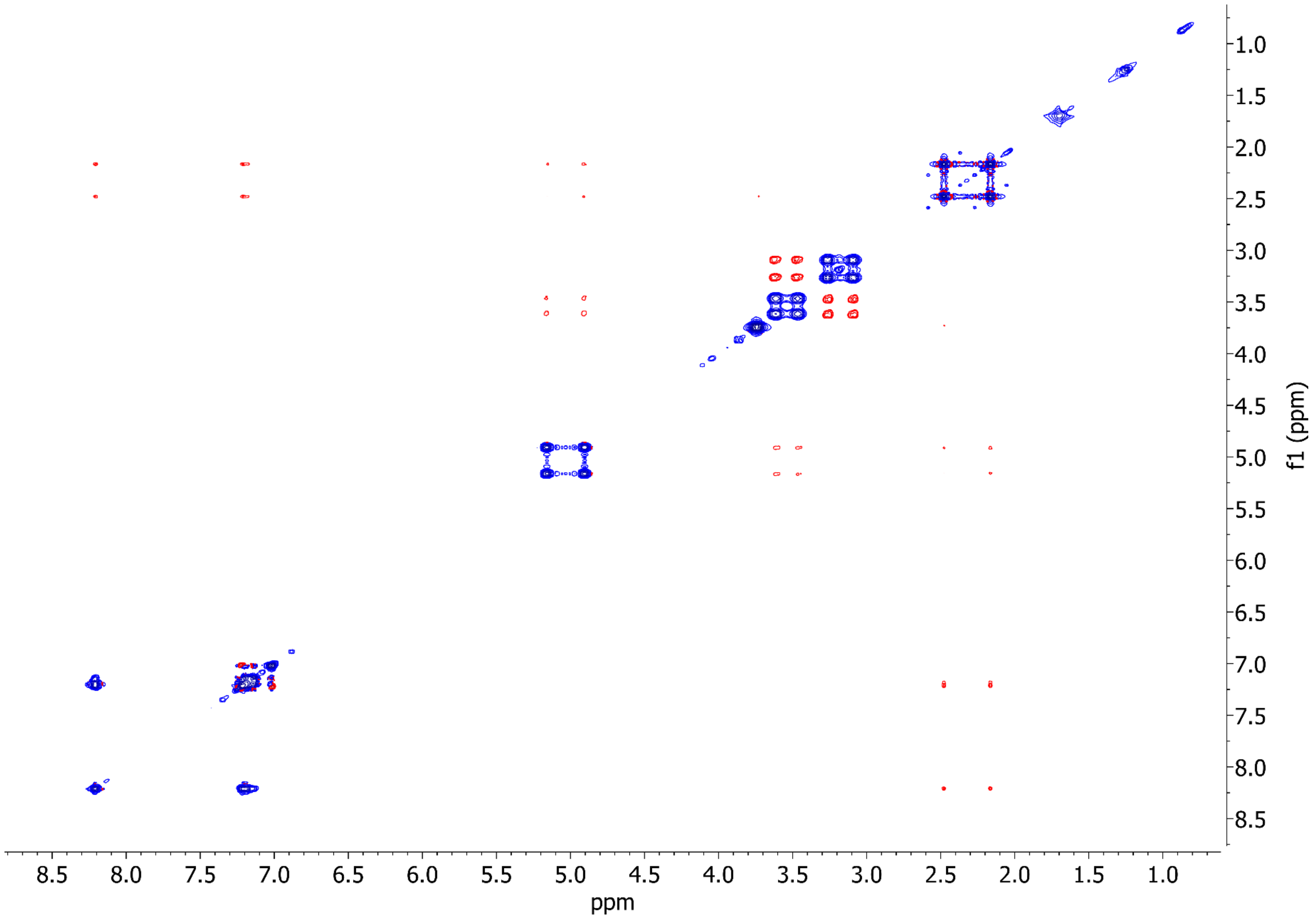

7. NOESY Correlations of Ac-(2S)-Ind-OMe (1) in DMSO-d6 at 600 MHz

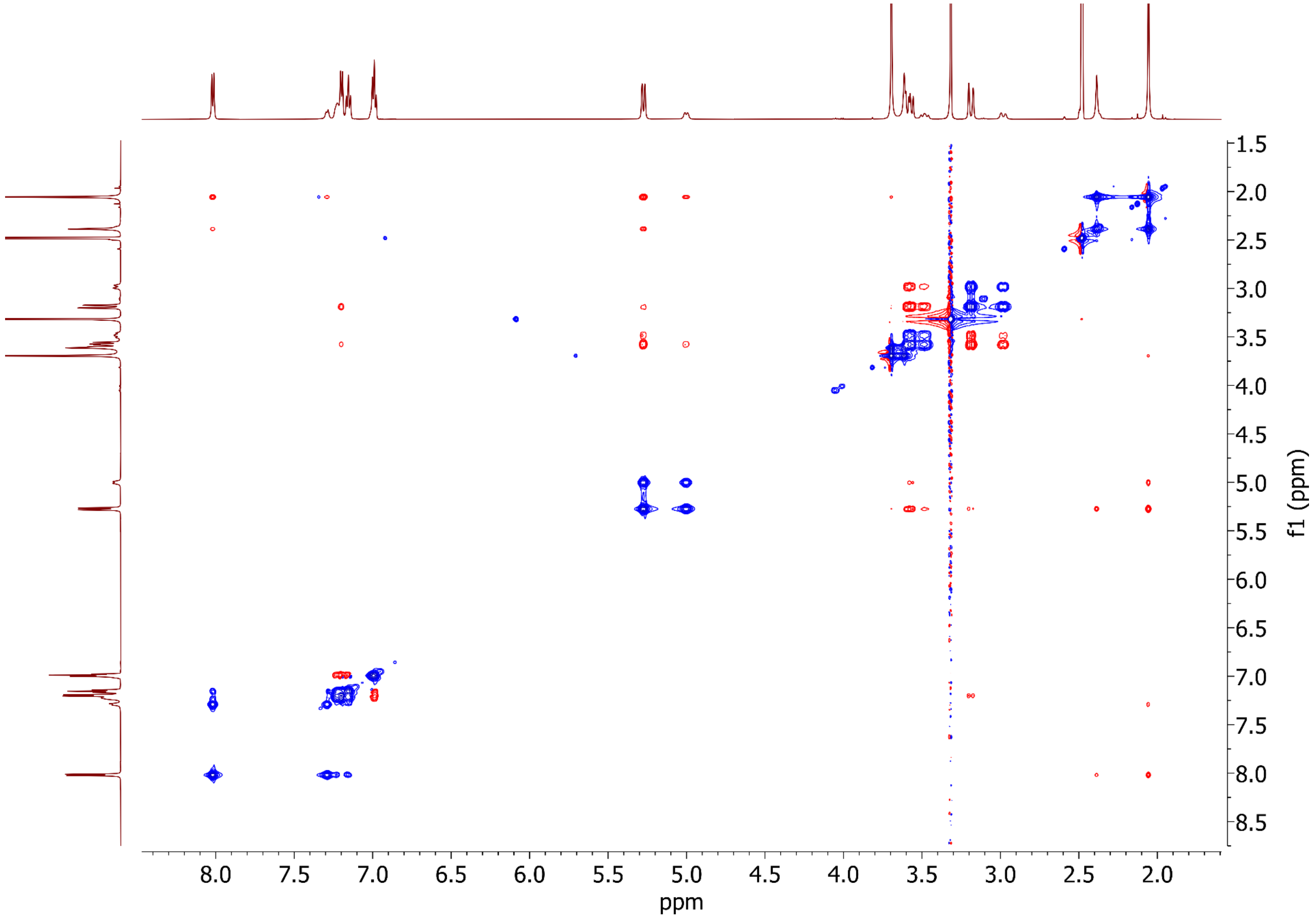

8. ^{1}H NMR spectrum of Ac-(2*S*)-Ind-OMe (1) in benzene-d6 (0.1 M) at 400 MHz

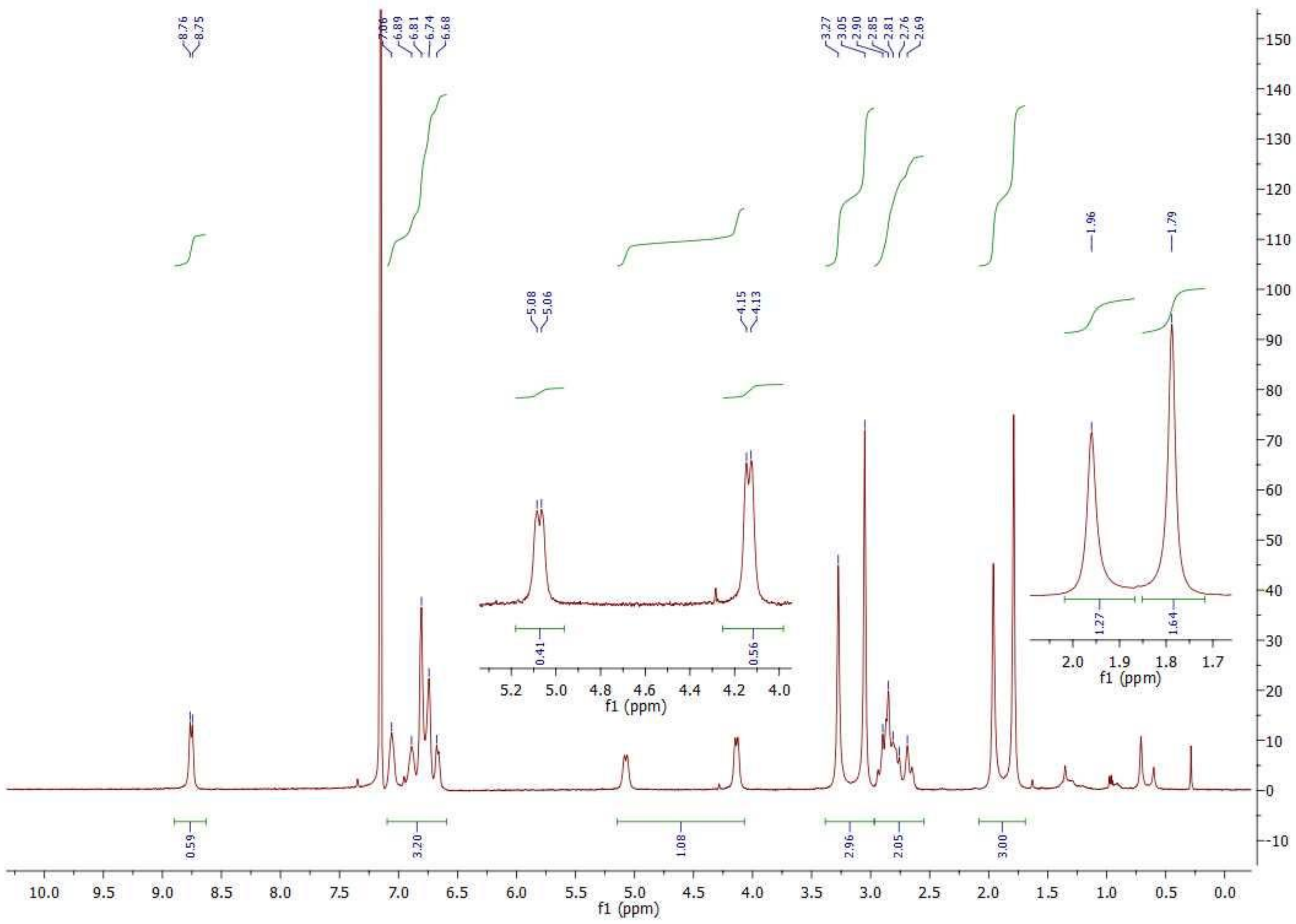

9. ^{1}H NMR spectrum of Ac-(2*S*)-Ind-OMe (1) in CD_2Cl_2 (0.1 M) at 400 MHz

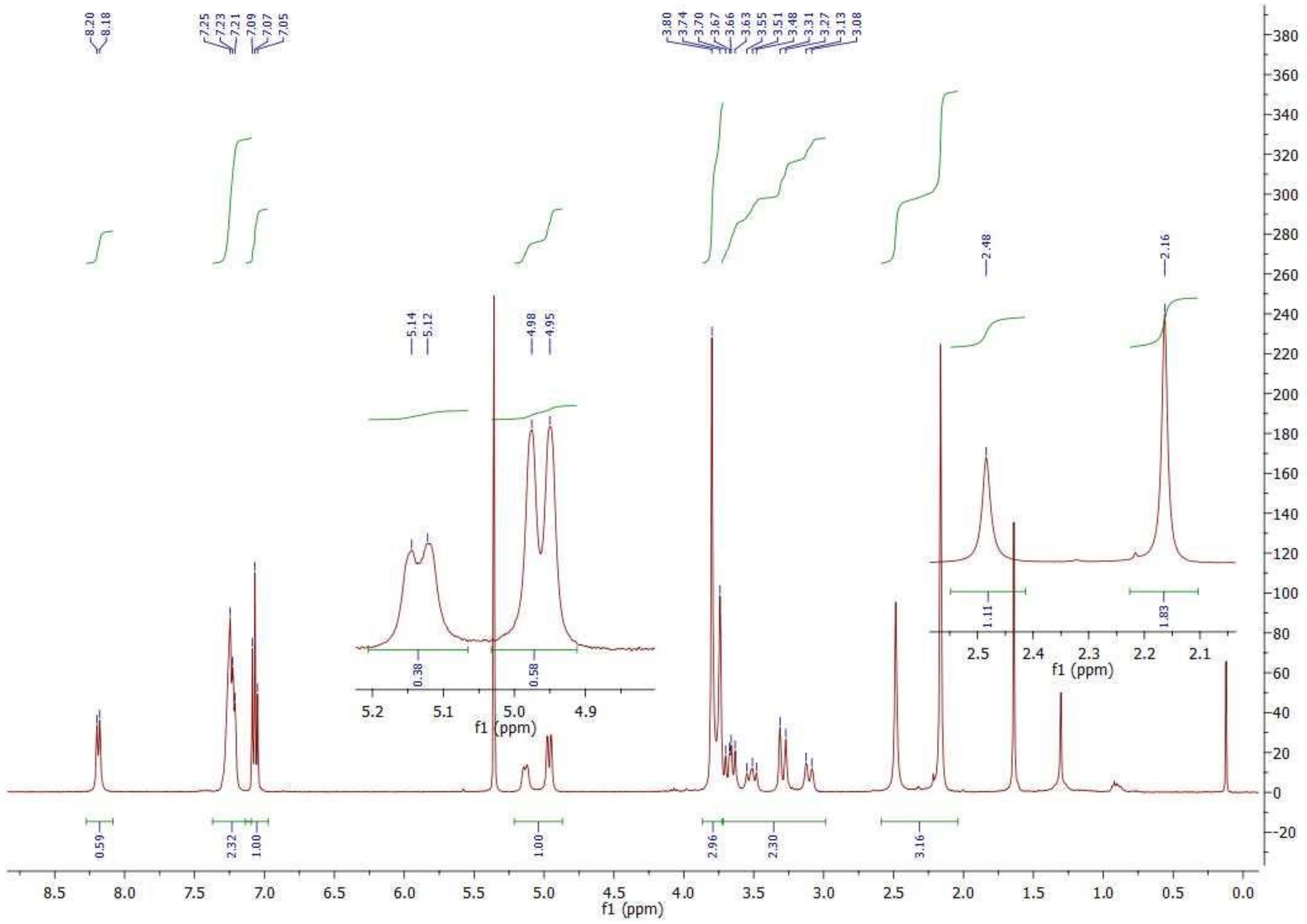

10. ^{1}H NMR spectrum of Ac-(2*S*)-Ind-OMe (1) in CD_3CN (0.1 mM) at 400 MHz

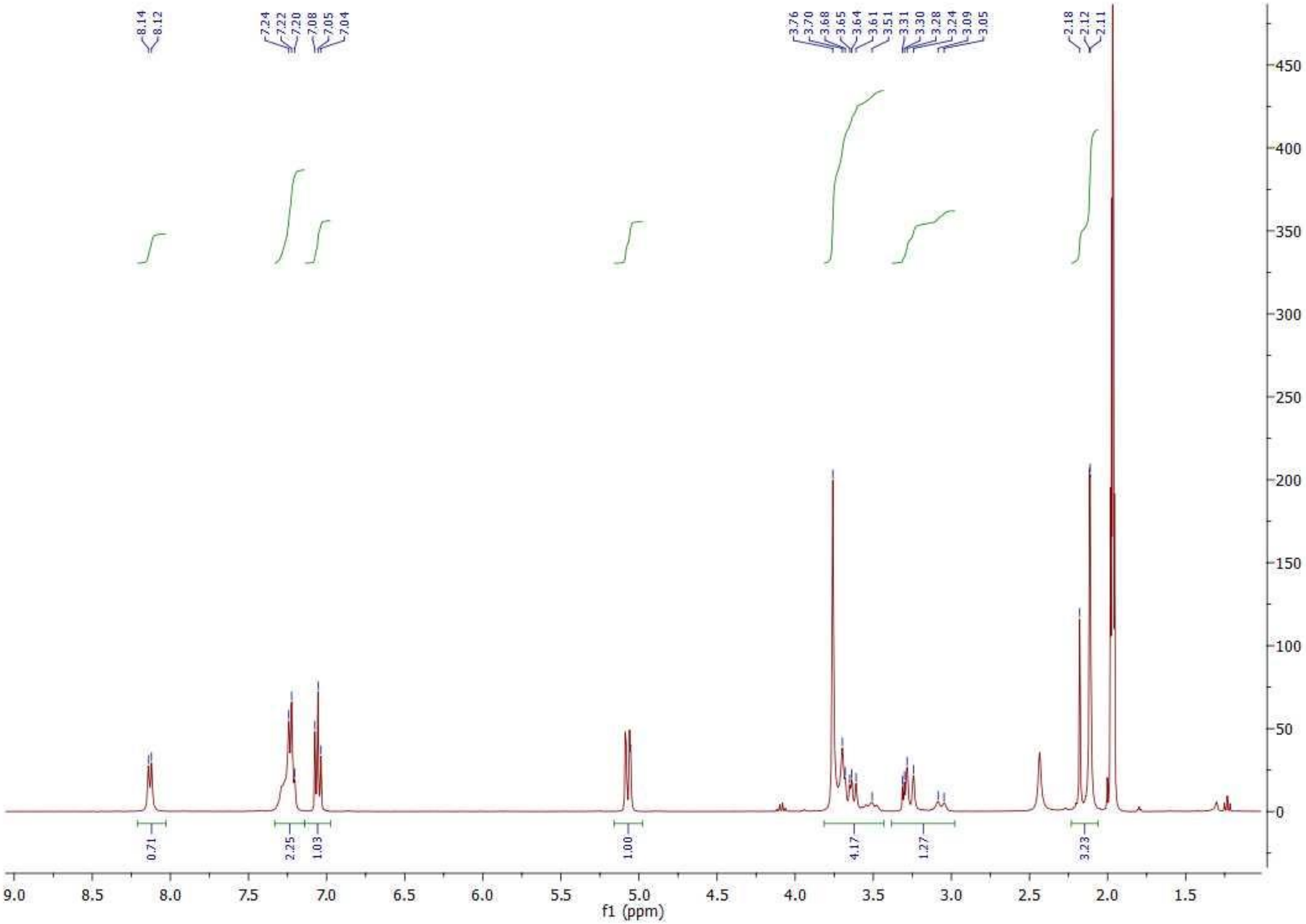

11. ^{1}H NMR spectrum of Ac-(2*S*)-Ind-OMe (1) in CD_3OD (0.1 M) at 400 MHz

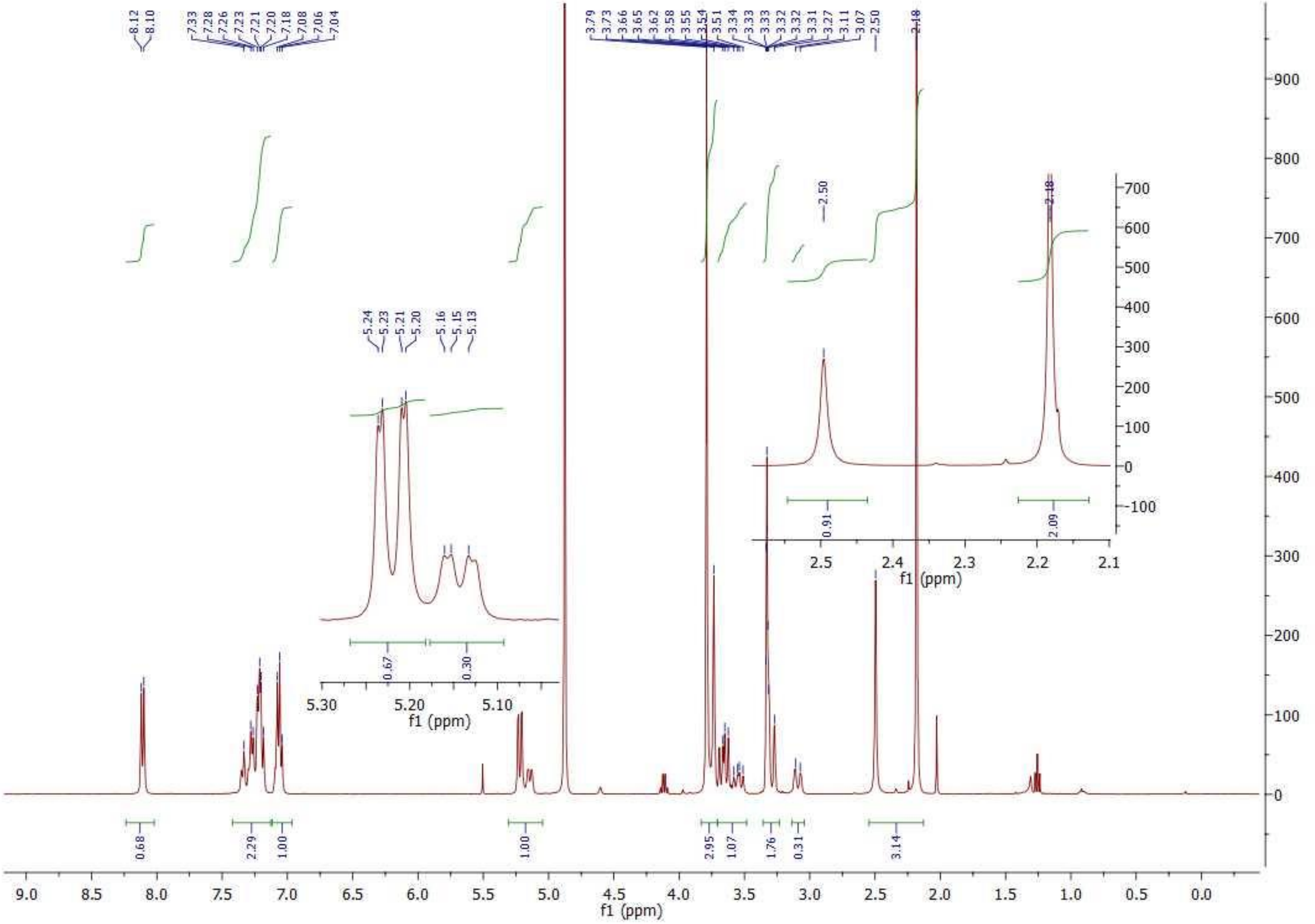

12. ^{1}H NMR spectrum of Ac-(2*S*)-Ind-OMe (1) in DMSO-d6 (0.1 M) at 400 MHz

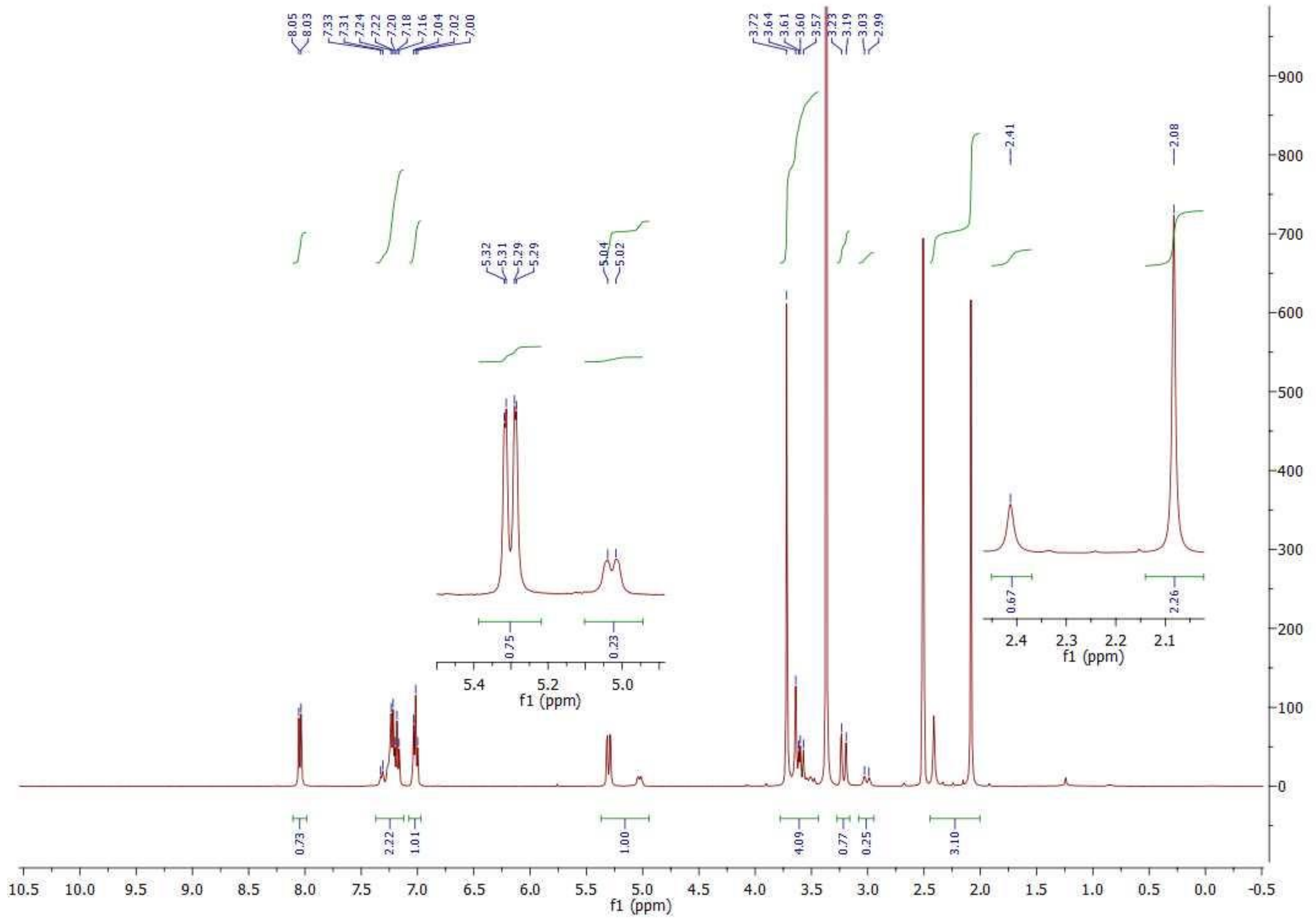

13. Variation of Ktrans/cis depending on the dielectric constants of the solvents

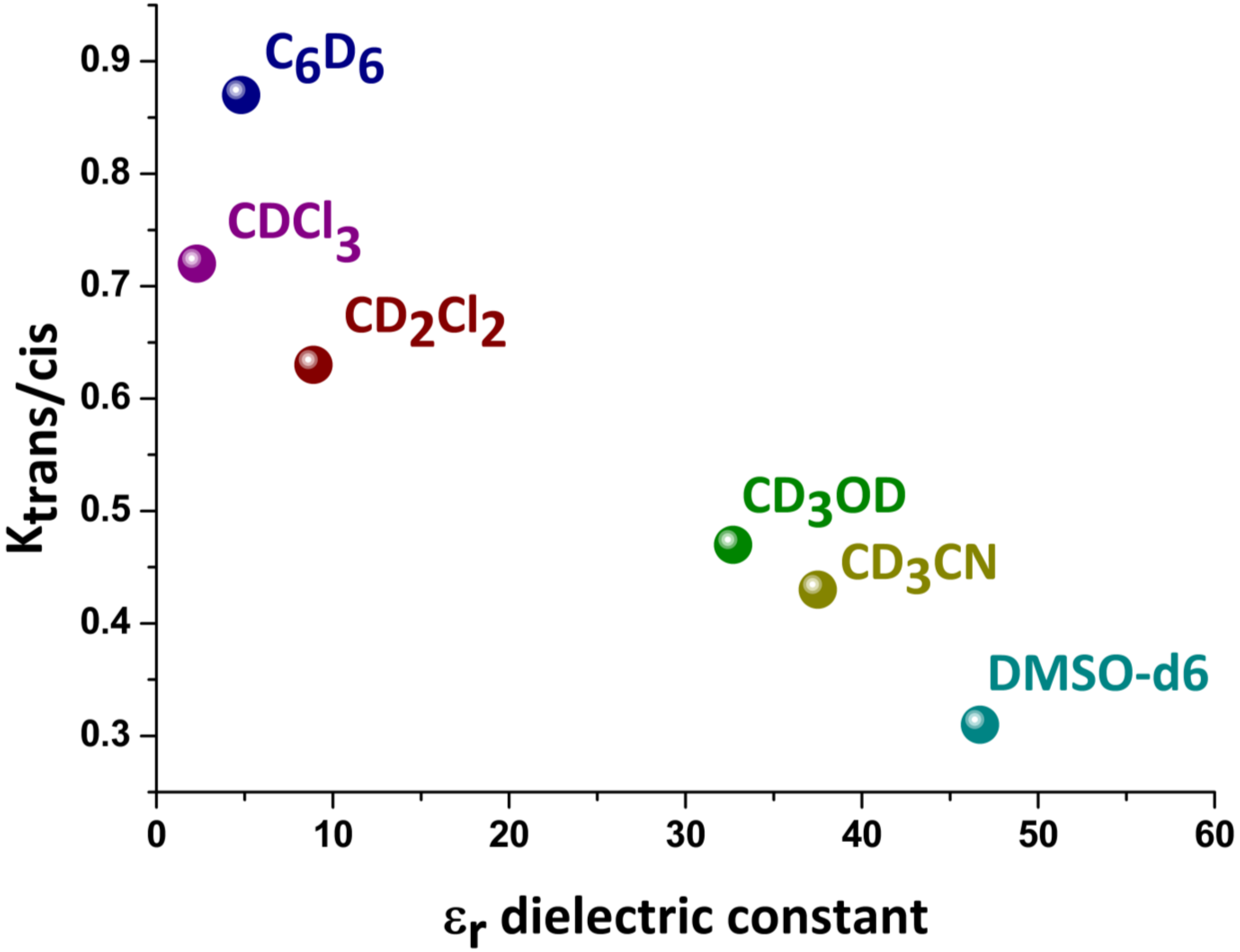

14. ^{1}H NMR of Ac-(2*S*)-Ind-OH in Acetic acid-d_4 at 400 MHz

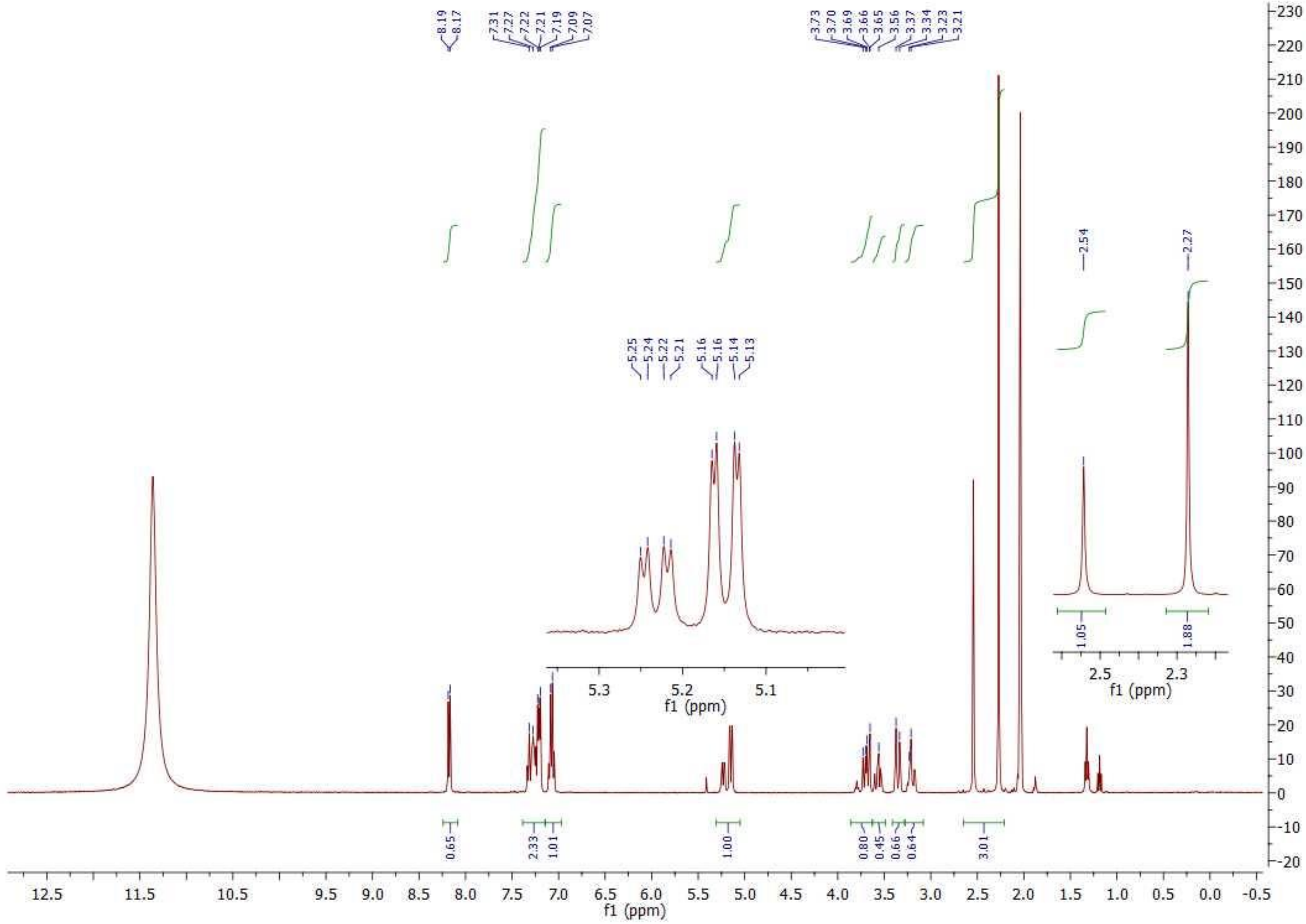

15. ^{1}H NMR of Ac-(2*S*)-Ind-OH in DMSO-d6 at 400 MHz

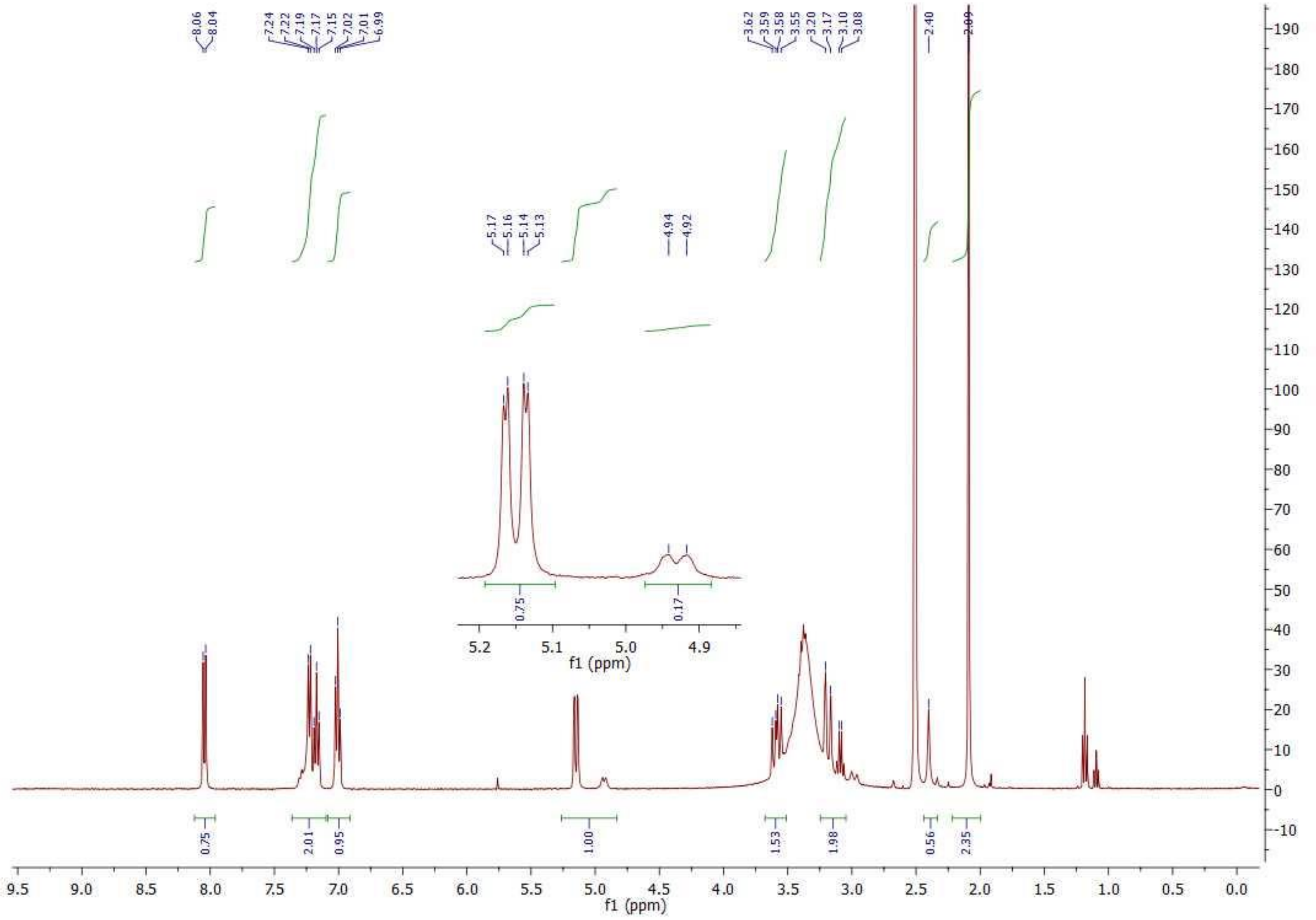

16. ^{1}H NMR spectrum of Ac-(2*S*)-Ind-OMe (1) in DMSO-d6 (0.1 M) at different temperatures at 400 MHz

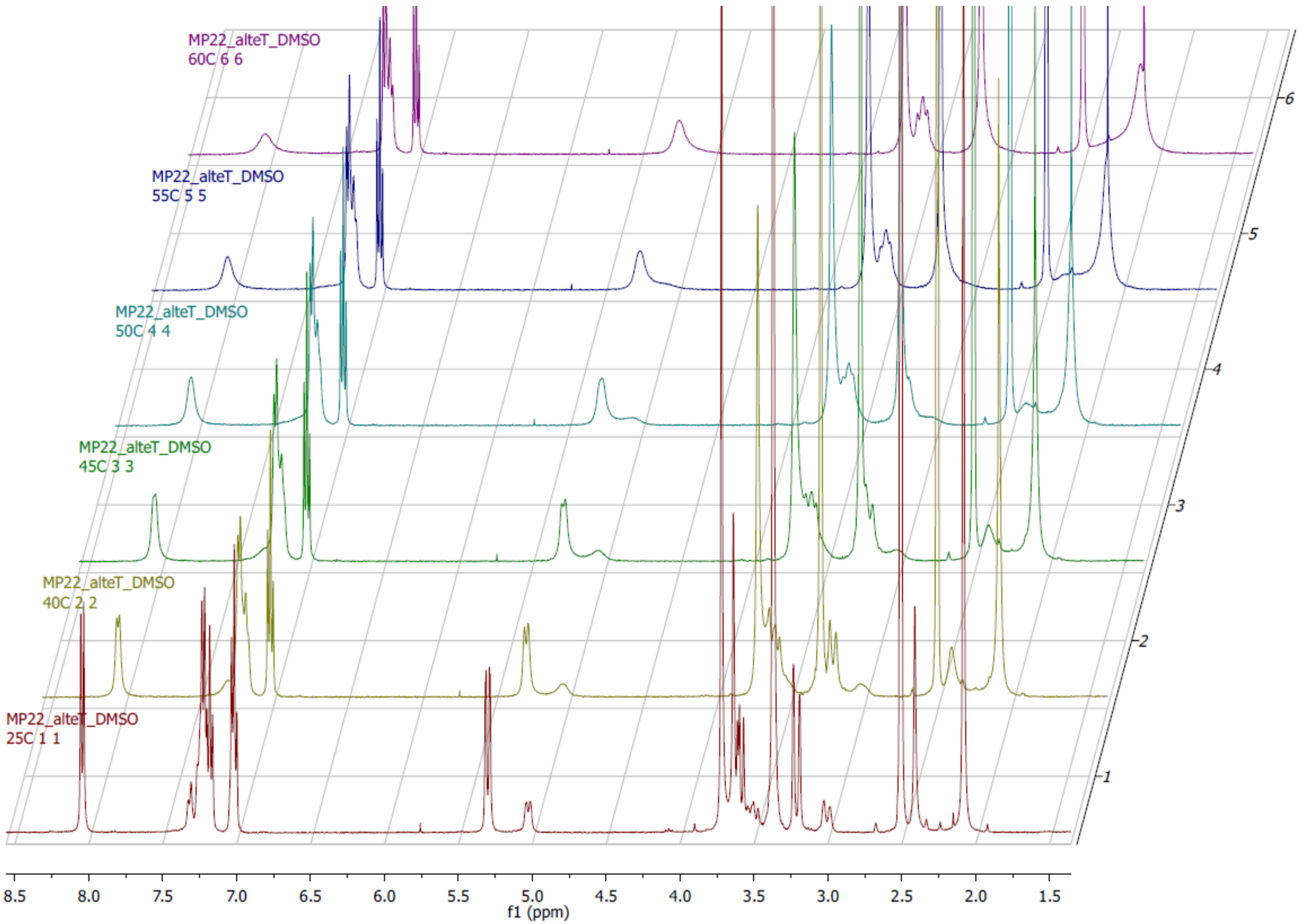

17. ^{1}H NMR spectrum of Ac-(2*S*)-Ind-OMe (1) in CD_2Cl_2 (0.1 M) at different temperatures at 400MHz

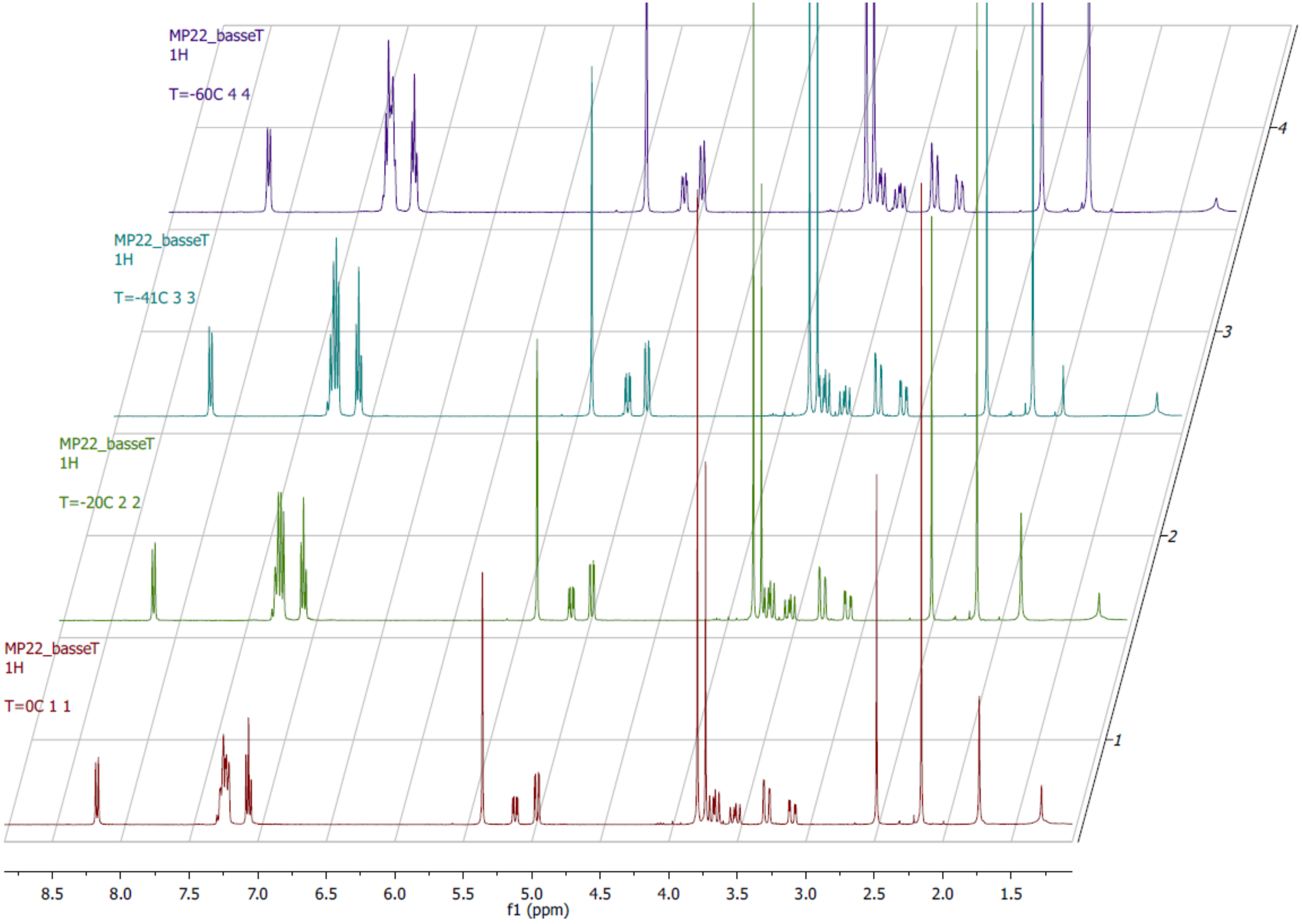

18. Titration of a 0.1 M solution of Ac-(2S)-Ind-OMe (1) in benzene-d6 (13.9 mg in 600 μL) with DMSO-d6 at 400 MHz. Variation of $K_{trans/cis}$ observed integrating the area of the two isomeric Hα.

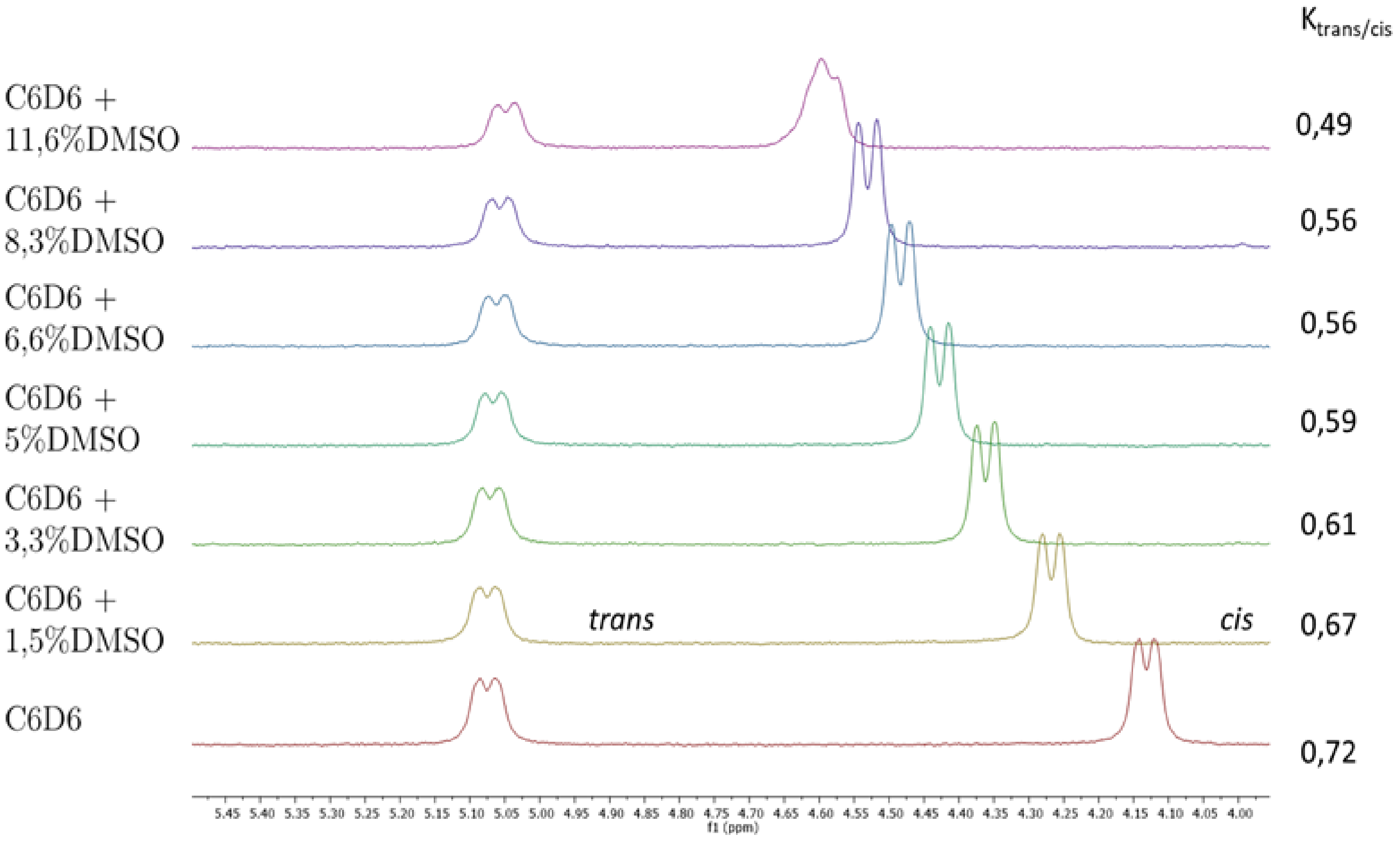

19. 2D EXSY map of Ac-(2*S*)-Ind-OMe (1) in DMSO-d6 (0.1 M) at 600 MHz

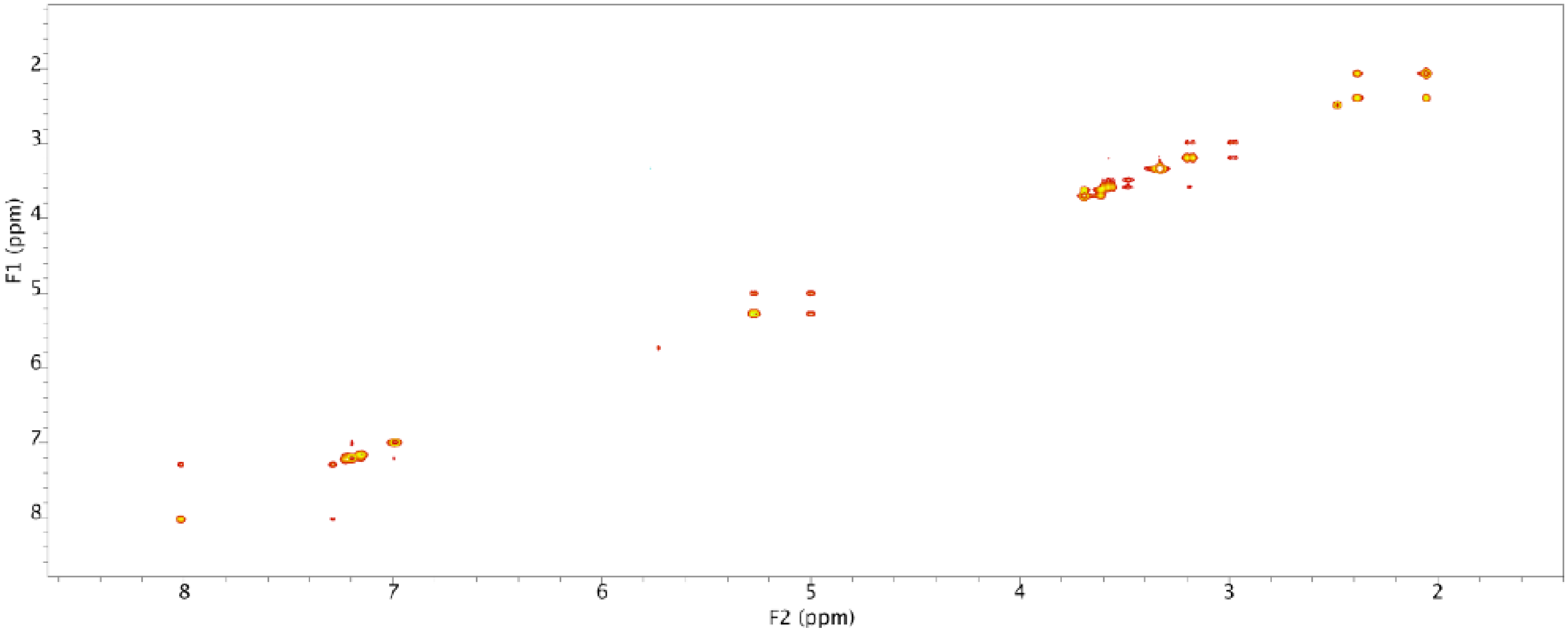

20. ^{1}H NMR spectrum of Ac-(2*S*)-Ind-OMe (1) in $CDCl_3$ (0.04 M) at 400 MHz

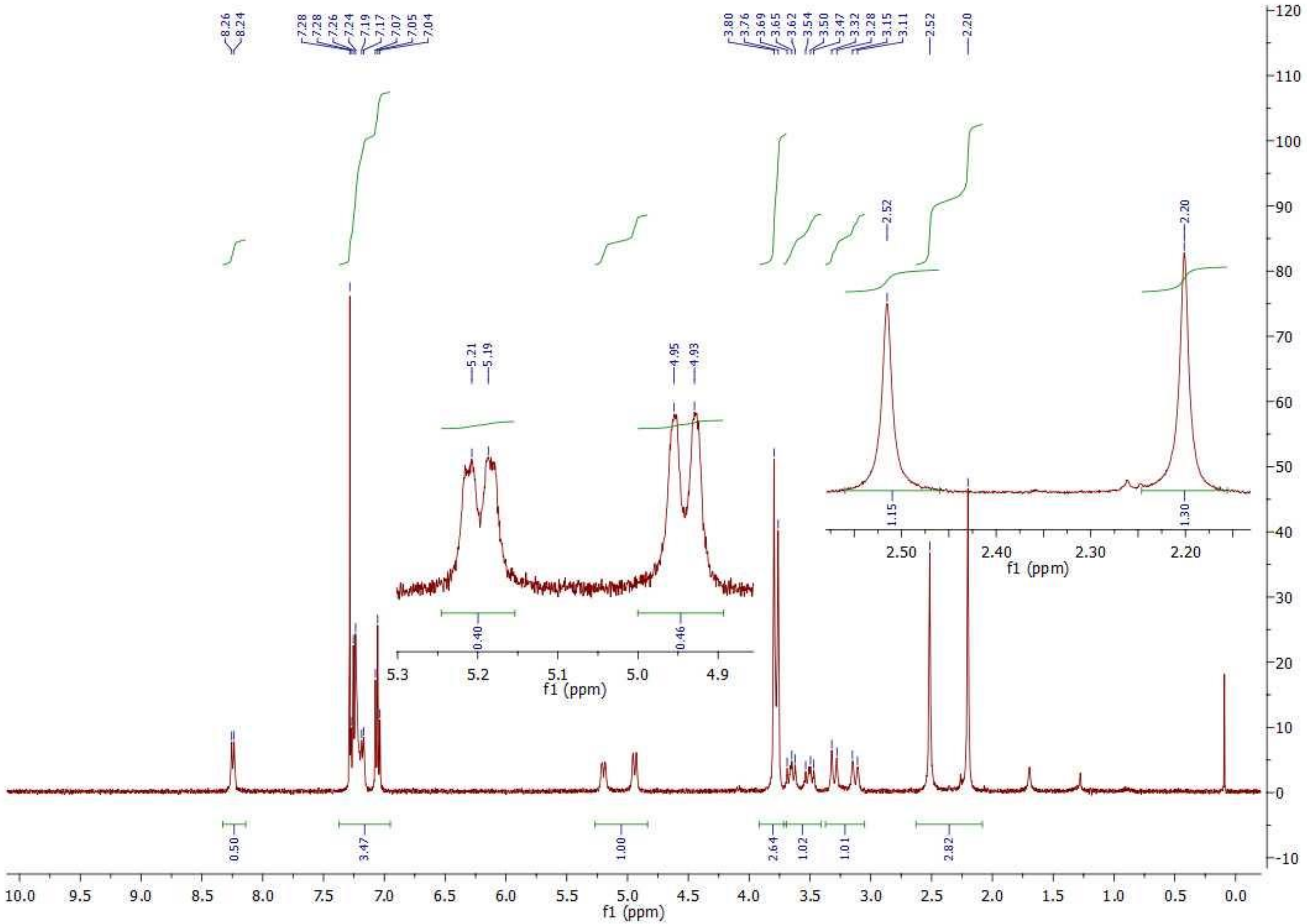

21. ^{1}H NMR spectrum of Ac-(2*S*)-Ind-OMe (1) in $CDCl_3$ (0.8 M) at 400 MHz

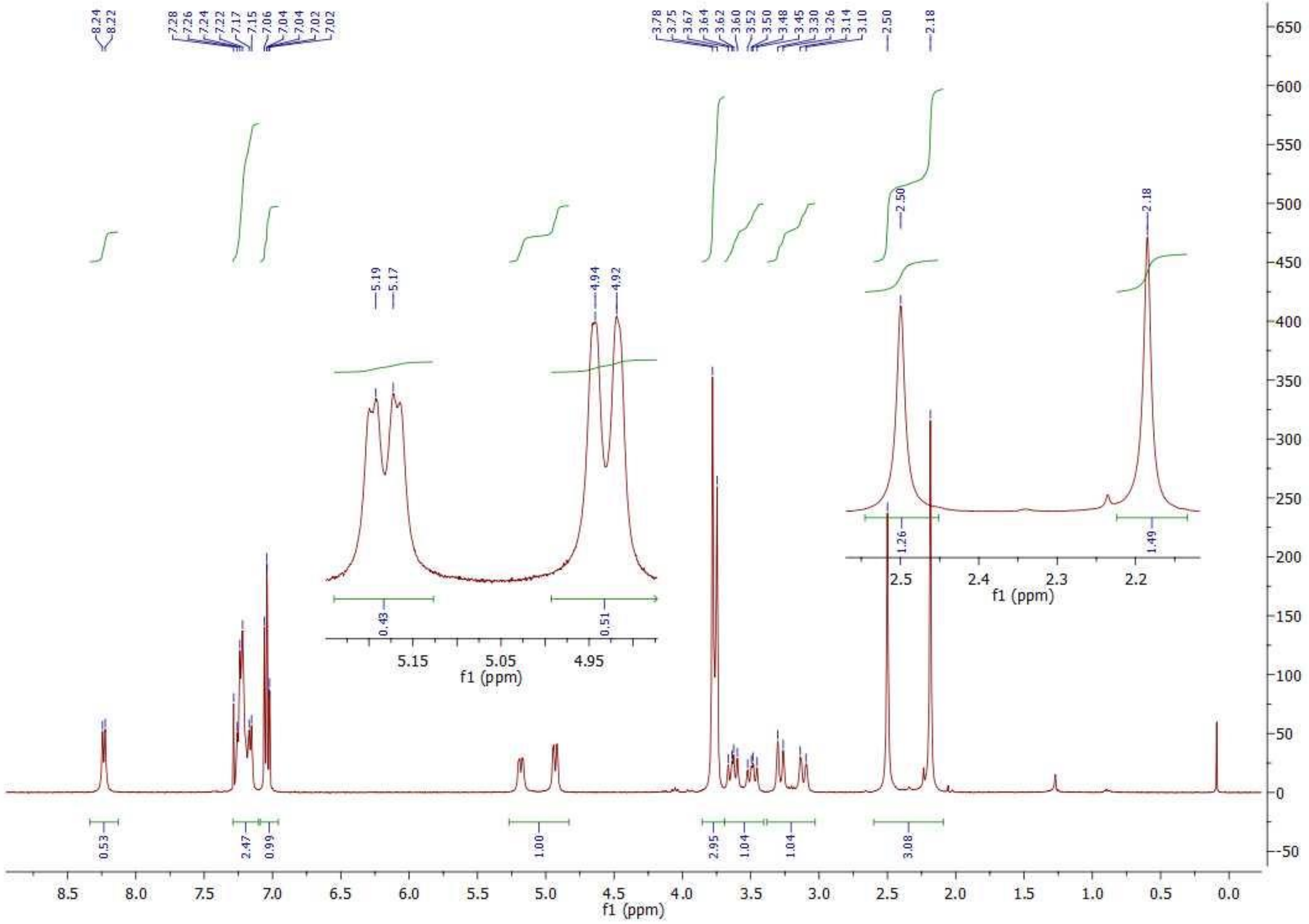

22. ¹H NMR of Ac-(2*S*)-Ind-(2*S*)-Ind-OMe (2) in $CDCl_3$ (0.1 M) at 600 MHz

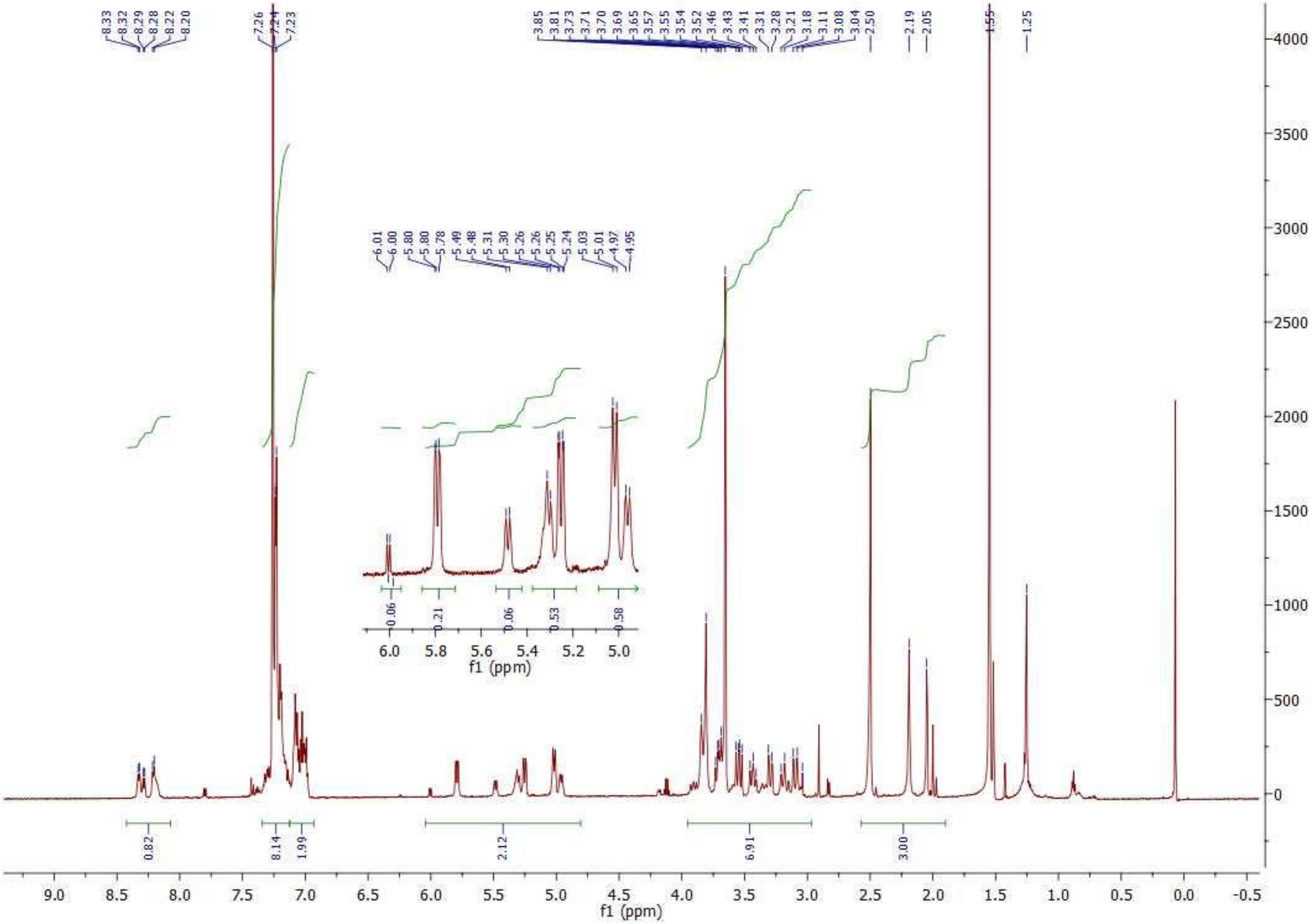

23. ^{1}H NMR of Ac-(2*S*)-Ind-(2*S*)-Ind-OMe (2) in DMSO-d6 (0.1 M) at 600 MHz

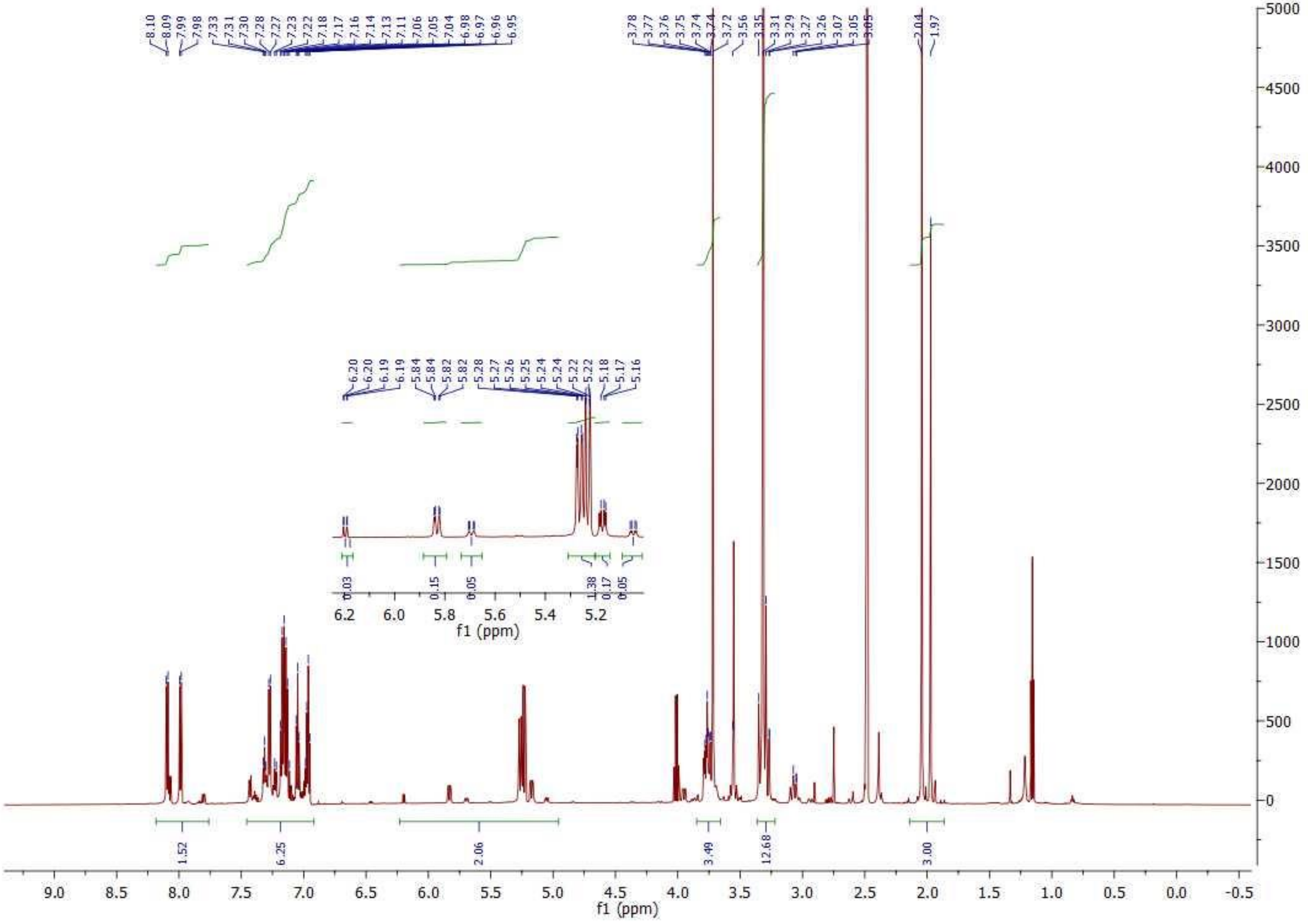

24. $^{13}C\{^1H\}$ NMR of Ac-(2*S*)-Ind-(2*S*)-Ind-OMe (2) in DMSO-d6 at 150 MHz

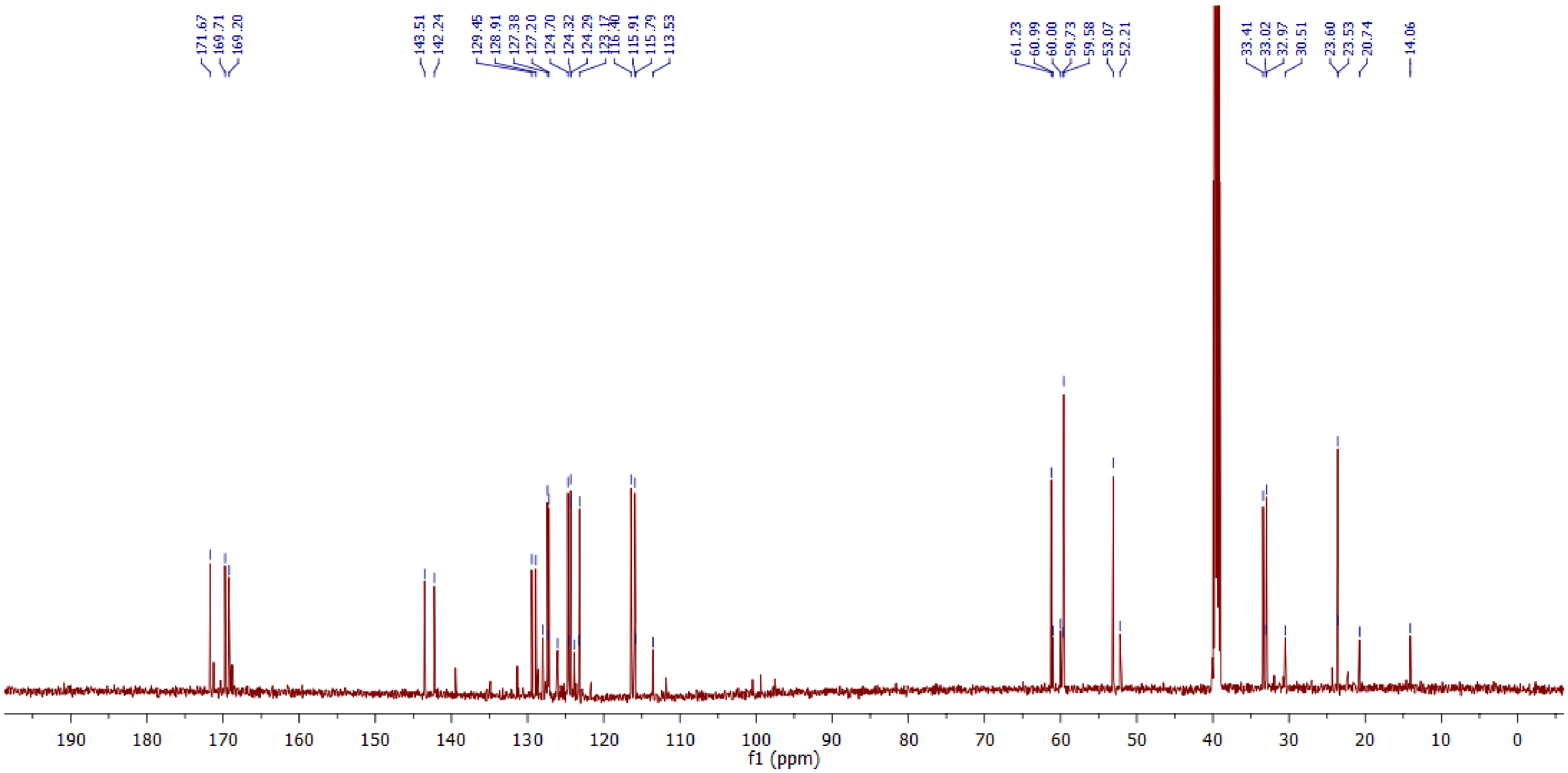

25. ^{1}H NMR signals assignment for cis-cis conformer of Ac-Ind-Ind-OMe (2) in DMSO-d6 on the basis of homonuclear scalar and dipolar correlations detected in COSY and ROESY maps

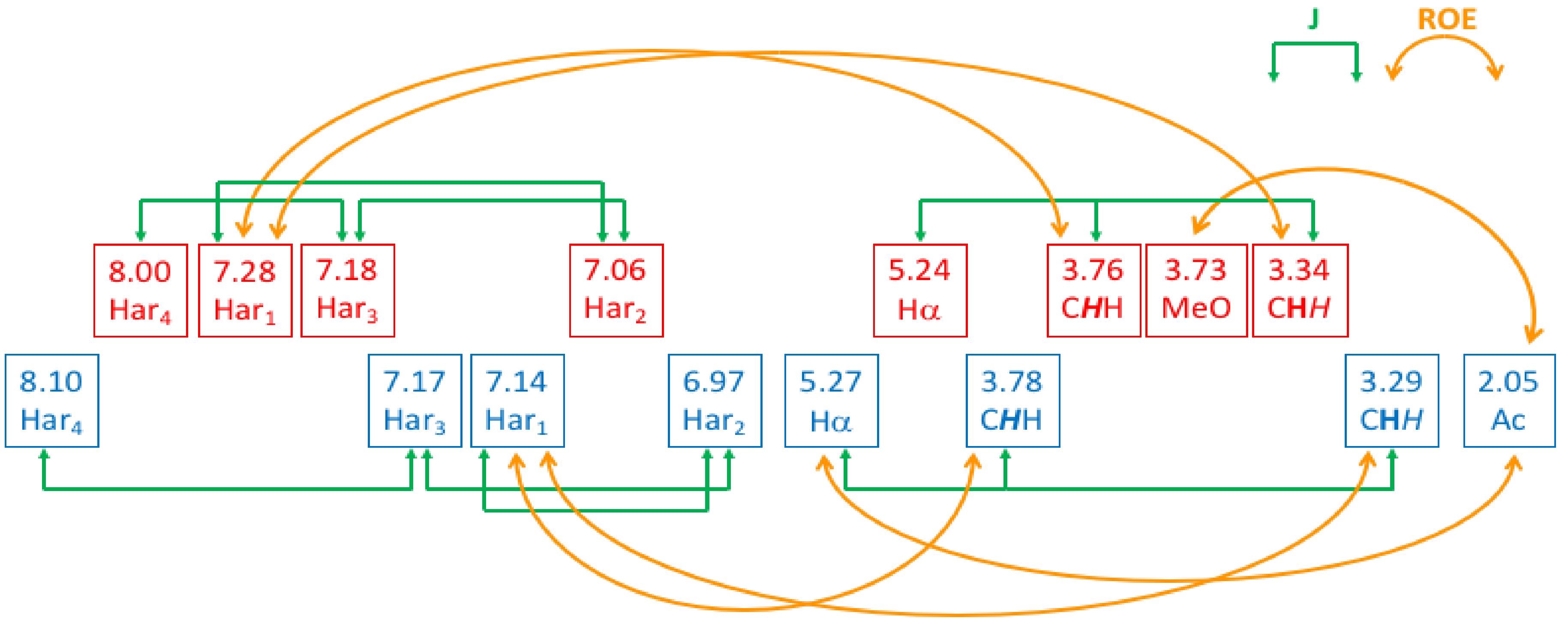

N-Ac terminal residue
methyl ester terminal residue

26. ROESY Maps of (2) in DMSO-d6

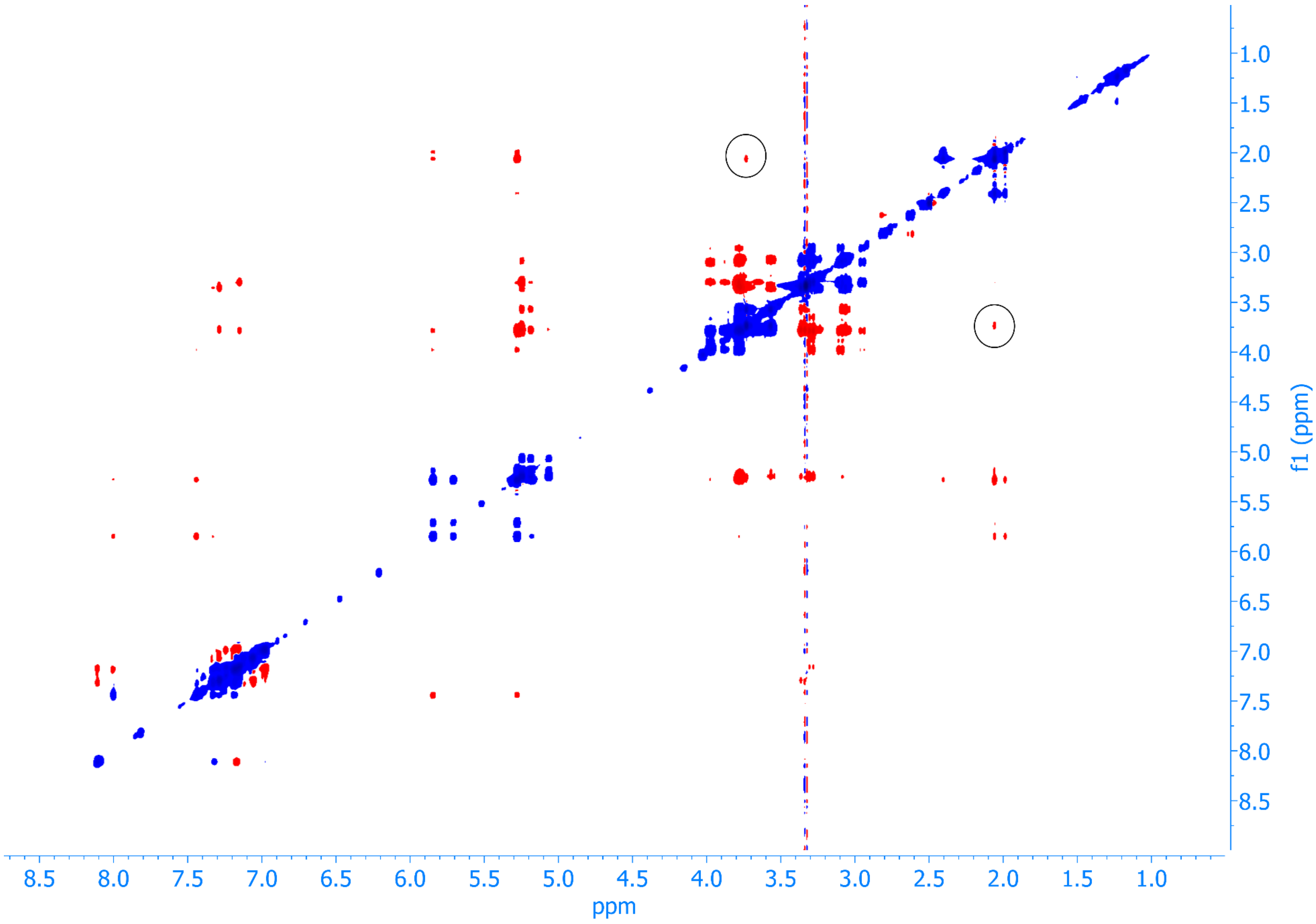

27. [1] HNMR characterization of the *cis*-*cis* species of (2)

Table. ^{1}H NMR chemical shift (δ, ppm, 600 MHz, DMSO-d6, 25 °C) of the main conformer of Ac-(2*S*)-Ind-(2*S*)-Ind-OMe (2)

	cis-*N*-acetyl terminal residue	*cis*-ester terminal residue
C**H**H	3.29	3.34
CH**H**	3.78	3.76
OMe	2.05	3.73
Hα	5.27 (dd, J =11.0 Hz, J=2.4 Hz)	5.24 (dd, J =10.3 Hz, J=1.4 Hz)
Har_1	7.14 (d, J=7.6 Hz)	7.28 (d, J=7.6 Hz)
Har_2	6.97 (t, J=7.6 Hz)	7.06 (t, J=7.6 Hz)
Har_3	7.17 (dd, J=8.1 Hz, J=7.6 Hz)	7.18 (dd, J=8.1 Hz, J=7.6 Hz)
Har_4	8.10 (d, J=8.1 Hz)	8.00 (d, J=8.1 Hz)

28. HPLC-MS analysis of compound (1)

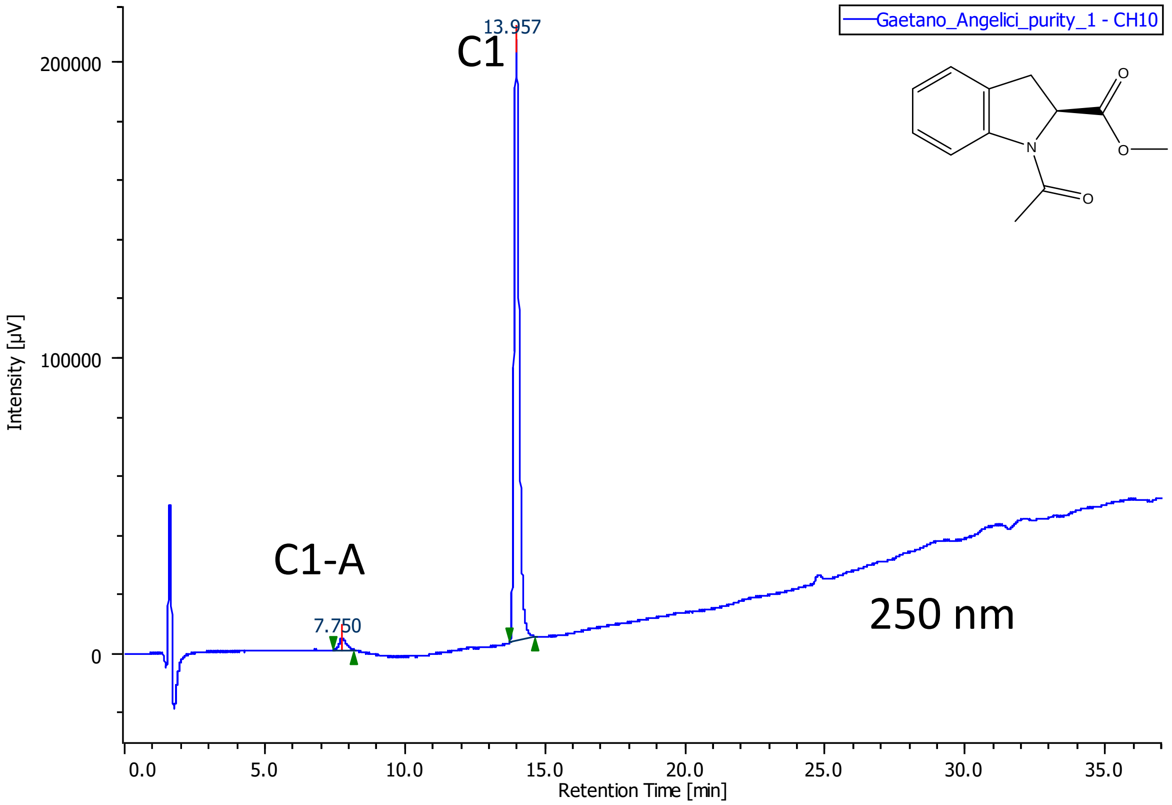

Peak	% a 250 nm
C1-A	3
C1	97

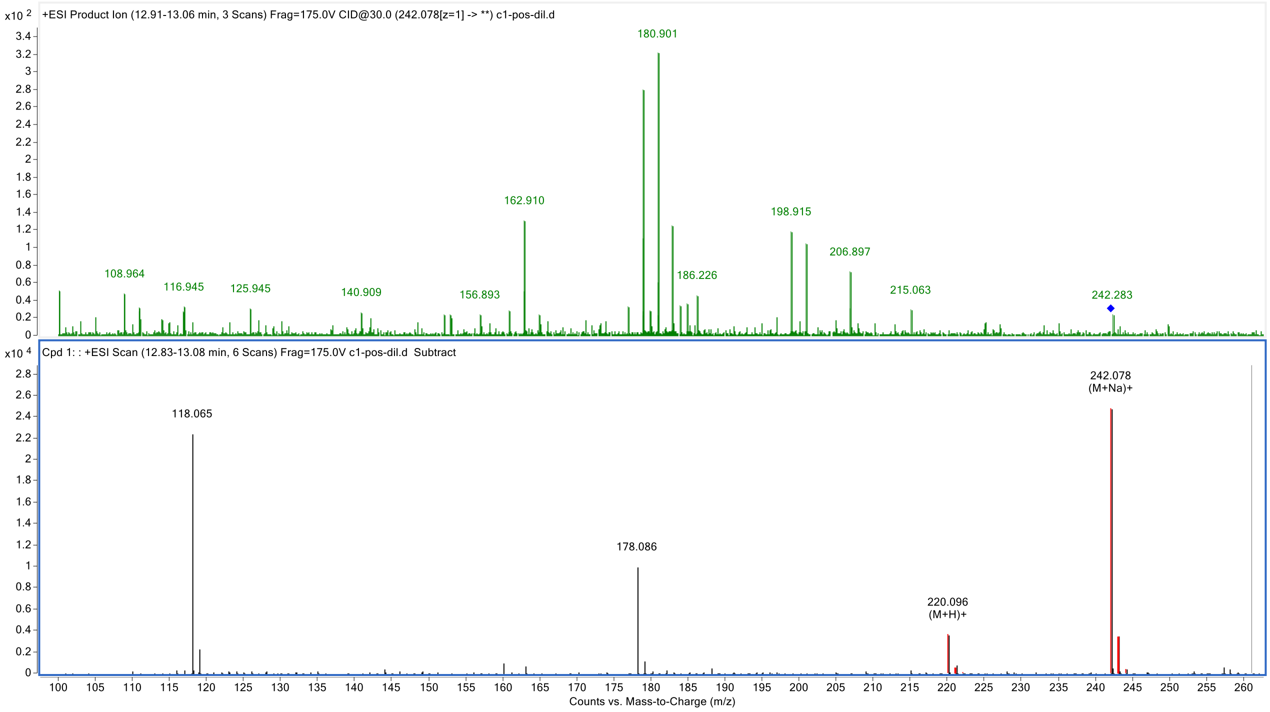
x10 2
+ESI Product Ion (12.91-13.06 min, 3 Scans) Frag=175.0V CID@30.0 (242.078[z=1] -> **) c1-pos-dil.d
180.901
162.910
198.915
206.897
186.226
108.964
116.945
125.945
140.909
156.893
215.063
242.283
x10 4
Cpd 1: : +ESI Scan (12.83-13.08 min, 6 Scans) Frag=175.0V c1-pos-dil.d Subtract
242.078
(M+Na)+
118.065
178.086
220.096
(M+H)+
Counts vs. Mass-to-Charge (m/z)

29. HPLC-MS analysis of compound (2)

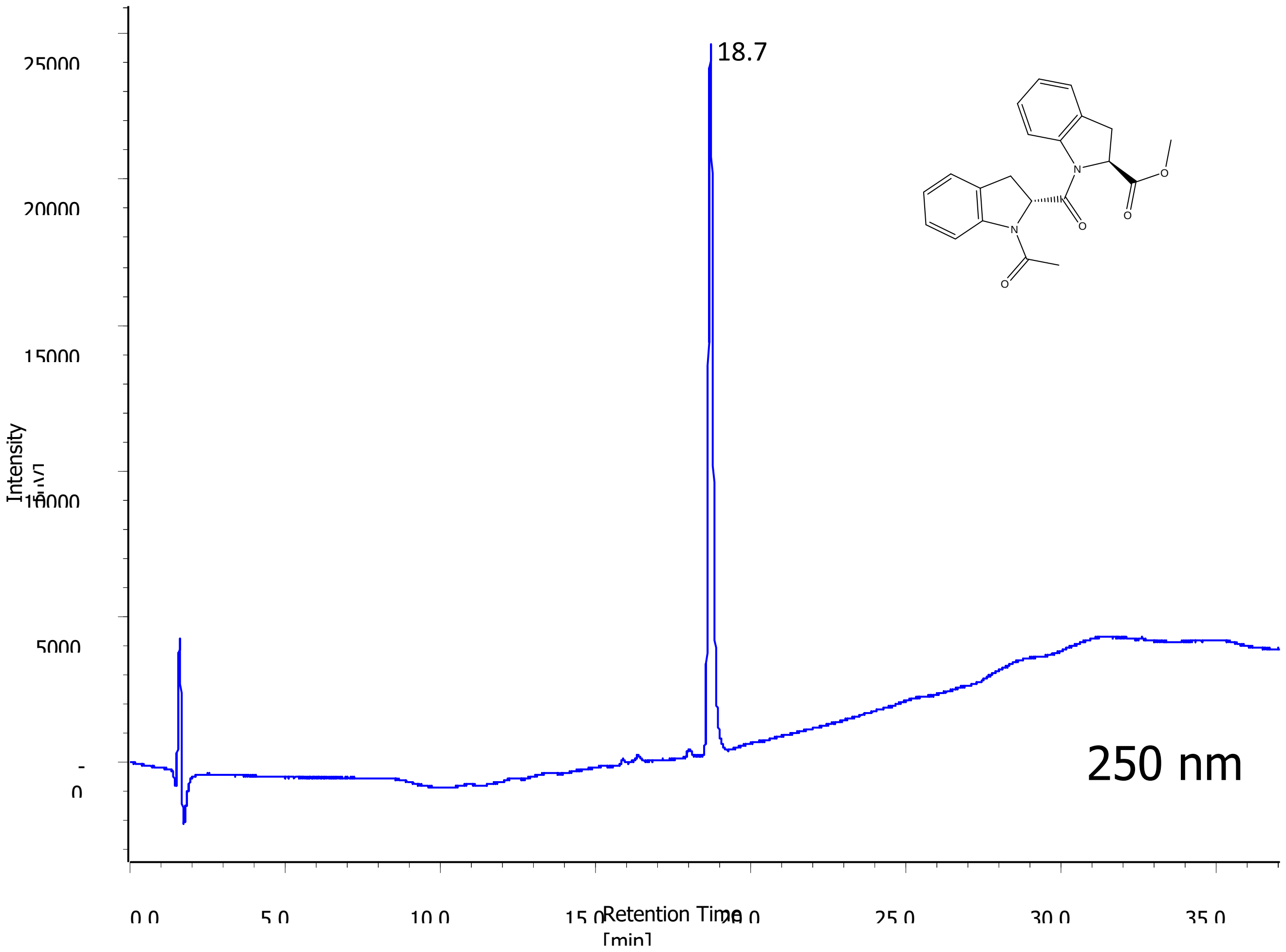

Peak	% a 250 nm
15.9	0.6
16.3	0.7
18.0	1.0
18.7	98

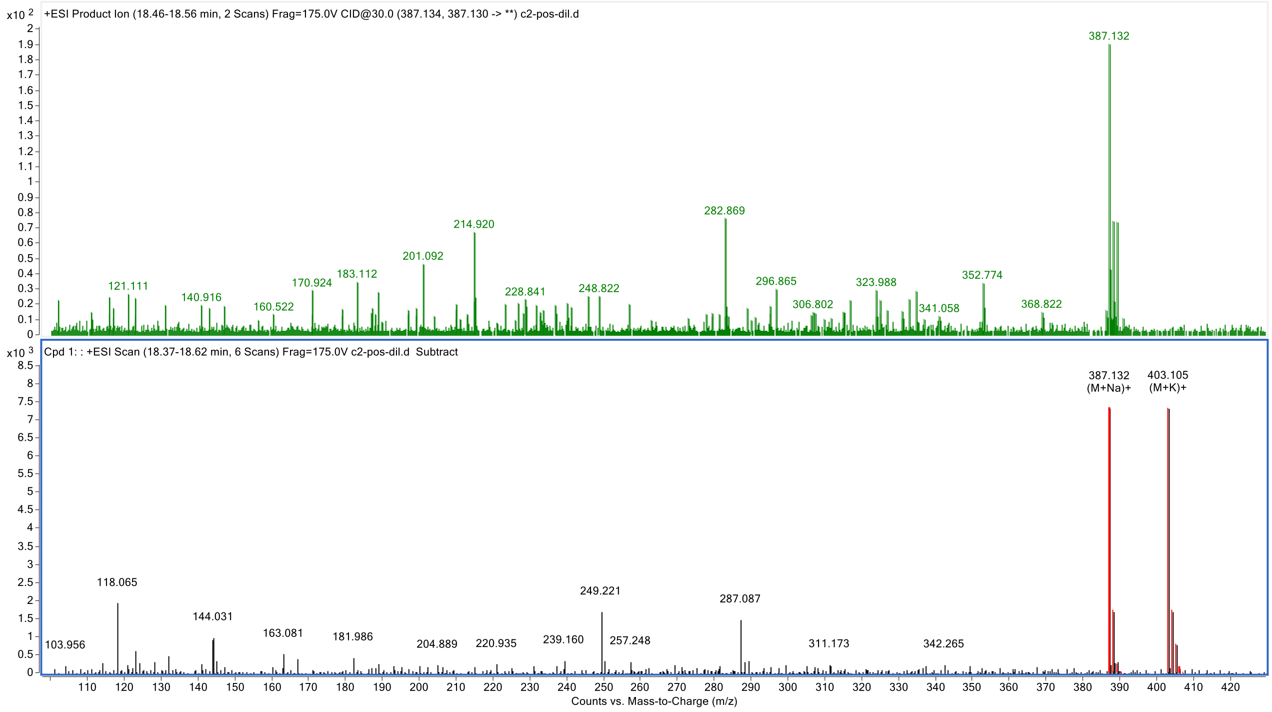

Compound	Formula	Base peak (ion)	Exact mass (m/z)	Experimental mass (m/z)	Δ (ppm)
(**1**)	C12H13NO3	**$[M+Na]^+$**	242.0788	242.0784	1.5
(**2**)	C12H20N2O4	**$[M+Na]^+$**	387.1315	387.1317	0.4

30. Correlation between cis→trans equilibrium and solvent polarity

According to the reaction field method based on Onsager's theory,[1] the electrostatic stabilization of a solute molecule, located in the centre of a spherical cavity, with dipole moment μ and radius r in a solvent modeled as a uniform dielectric with relative permittivity ε_r, can be expressed as:

$$\Delta G^0_{solv} = -\frac{N_A}{4\pi\varepsilon_0}\frac{\varepsilon_r - 1}{2\varepsilon_r + 1}\frac{\mu^2}{r^3} \quad \text{[SI1]}$$

where

$$X = (\varepsilon_r - 1)/(2\varepsilon_r + 1) \quad \text{[SI2]}$$

is the dielectric function. The difference in free energy of solvation for two species in equilibrium, such as the *cis* and *trans* isomers of **1**, is given by:

$$\Delta\Delta G^0_{solv} = -\frac{N_A}{4\pi\varepsilon_0}X\left(\frac{\mu^2_{cis}}{r^3} - \frac{\mu^2_{trans}}{r^3}\right) \quad \text{[SI3]}$$

assuming they have the same radius *r*. Quantitatively, this expression overestimates the solvent effects obtained experimentally for various conformational equilibria. An empirical correlation which better reproduces experimental data uses a parabolic rather than a linear relationship with X:[2]

$$\Delta\Delta G^0_{solv} = -RT\,log\,K_{cis/trans} = A + B\sqrt{0.5 - X} \quad \text{[SI4]}$$

The graphic below shows a plot of experimental $\Delta G^0_{cis/trans}$ of **1**, obtained by NMR data (see Table 1 in main text), against X and their fitting according to eq. [SI4].

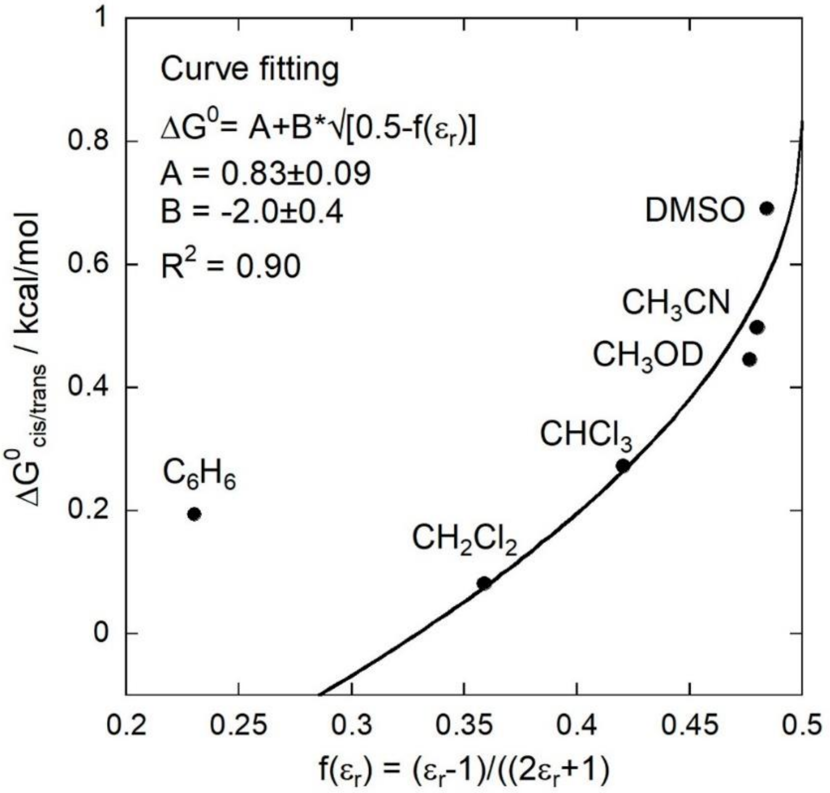

[1] Christian Reichardt and Thomas Welton. *Solvents and Solvent Effects in Organic Chemistry*, 4th ed.; WILEY-VCH Verlag GmbH & Co. KGaA, Weinheim, 2011.

[2] V. V. Samoshin and N. S. Zefirov, *Tetrahedron Lett.* **1981**, *22*, 2209-2212.

31. Representative van't Hoff plot based on the cis/trans equilibrium

Estimated by NMR integration data of the variable temperature experiment performed on a 80 mM DMSO-d6 solution of (1).

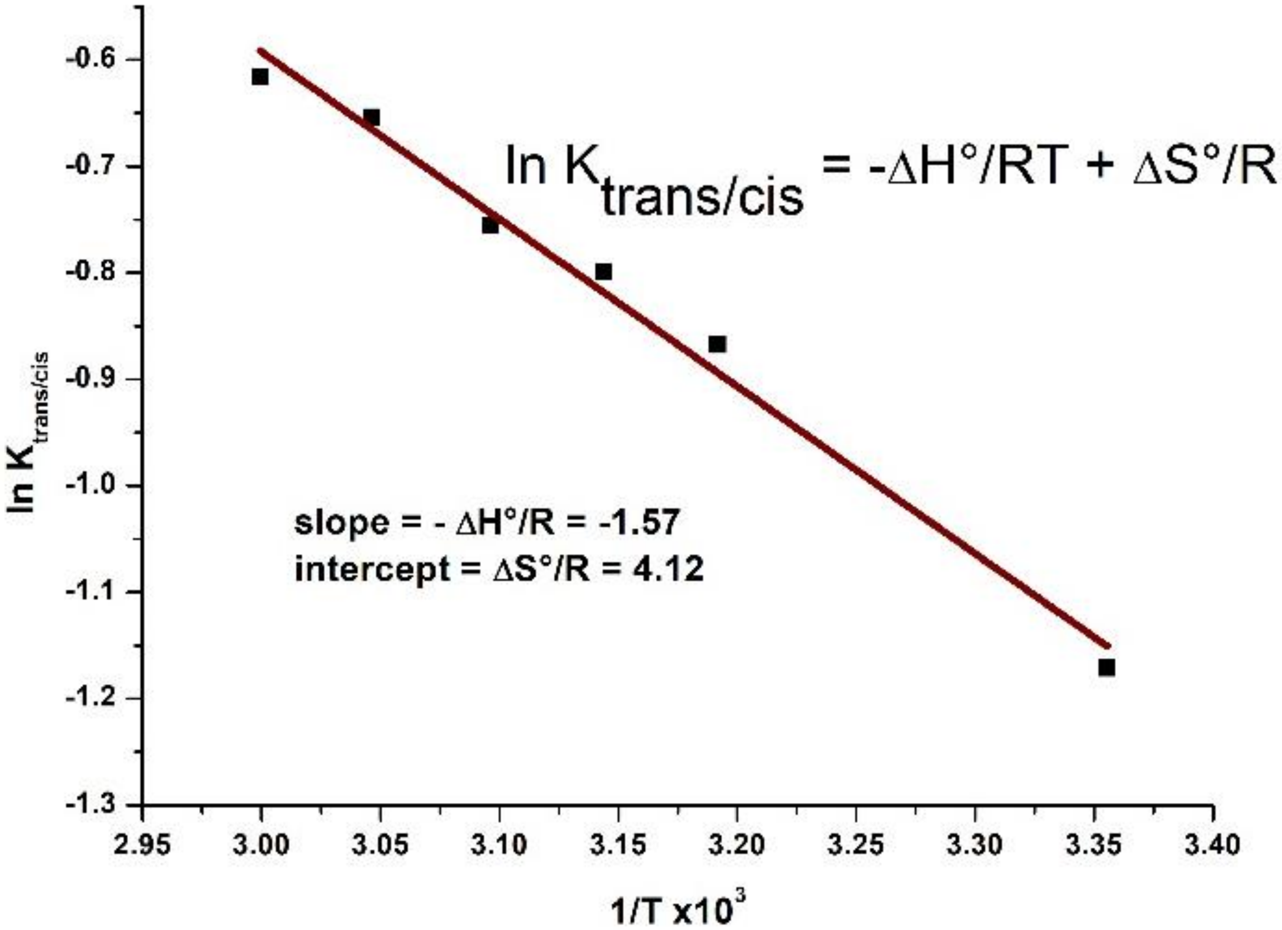

32. Computed absolute free energies (in atomic units) of the various conformers of Ac-(2S)-Ind-OMe (1) in various solvents.

All the data have been obtained using the B3LYP DFT functional augmented with Grimme's D3 dispersion corrections in conjunction with the 6-311+G(d) basis set. Solvation effects have been introduced using the integral equation formalism polarizable continuum model (IEFPCM). All the calculations have been performed using Gaussian 16.

Conformer	Acetonitrile	Benzene	Chloroform	Dichloromethane	DMSO	Methanol
T1	-745,624241	-745,616142	-745,619911	-745,622072	-745,624436	-745,624163
T2	-745,624399	-745,616141	-745,620058	-745,622241	-745,624582	-745,624326
C1	-745,624758	-745,616809	-745,620596	-745,622683	-745,624939	-745,624688
C2	-745,625205	-745,616584	-745,620571	-745,622871	-745,625416	-745,625124

33. Computed dipole moments of the Cis and Trans isomers of Ac-(2S)-Ind-OMe, in kcal/mol.

Solvent	C	T
Benzene	4.24	4.37
$CHCl_3$	3.62	4.62
CH_2Cl_2	4.89	3.69
CH_3OH	3.68	5.19
CH_3CN	4.48	3.73
DMSO	3.65	5.23

34. Analysis of n-π* interaction

Data obtained at B3LYP-D3BJ/6-311+G(d)/IEFPCM (DMSO) level of calculation.

Compound, *Conformer*	d (Å)[2]	θ (°)[2]	Θ (°)[2]	Δ (Å)[2]	n-π* NBO overlap [3]
Ac-Ind-OMe, *T1*	2.97	114	2.57	0.021	0.052
Ac-Ind-OMe, *T2*	3.02	95.8	2.01	0.017	0.045
Ac-Ind-OMe, *C1*	4.34	nr	1.18	0.010	0.004
Ac-Ind-OMe, *C2*	4.29	nr	0.93	0.008	0.004
Ac-Pro-OMe, C^{γ}*-endo*	3.06	95.6	2.03	0.017	0.043
Ac-Pro-OMe, C^{γ}*-endo* [1]	3.06	99.4	2.41	0.02	0.075

[1] Literature data from A. Choudhary, D. Gandla, G.R. Krow and R.T. Raines, *J. Am. Chem. Soc.* 2009, 131, 21, 7244–7246, calculated at B3LYP/6-311+G(2d,p) level in vacuo.

[2] Defined according to the following scheme:

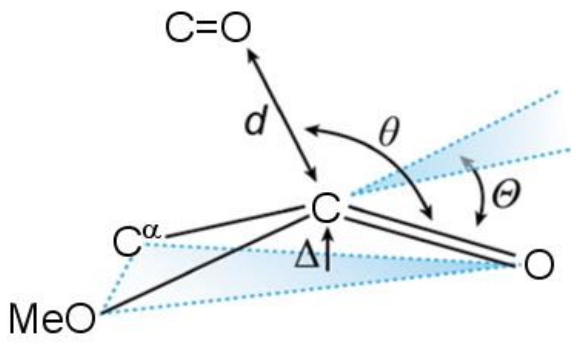

[3] Overlap between n(C=O) and π*(COOMe) natural bond orbitals (NBO), estimated with Multiwfn (v. 3.3.8), T. Lu and F. Chen, *J. Comput. Chem.* **2012**, *33*, 580-592. The plots below show the overlap for conformers T1 (left) and T2 (right).

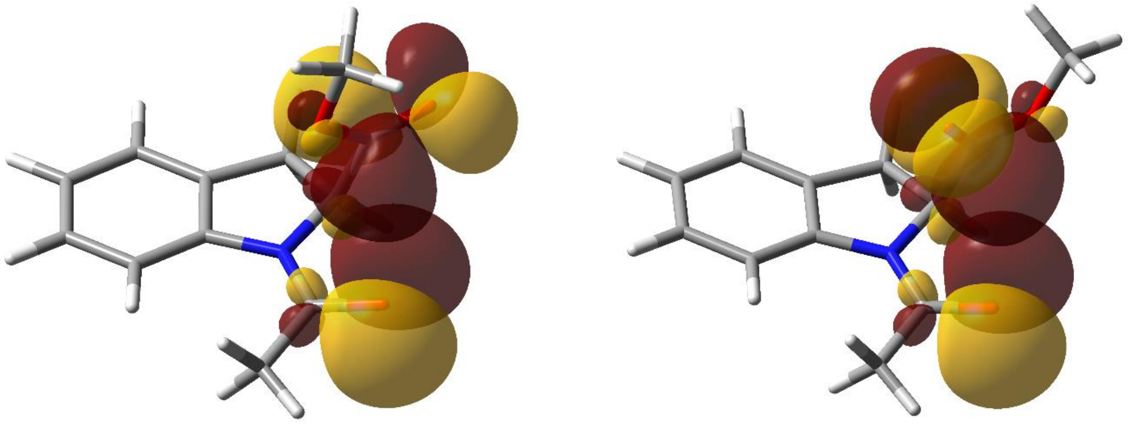

Acetonitrile Trans 1
SCF Energy: -745.814907818 A.U.
Free Energy: -745.624241 A.U.

Atom	Coordinates (Angstroms) X	Y	Z
C	2.555171	-1.672795	-0.367415
C	2.163913	0.922896	0.652323
C	3.554902	-1.057844	0.392719
C	1.376810	-0.988991	-0.617183
C	1.181536	0.306792	-0.120490
C	3.351324	0.225243	0.895242
N	-0.106046	0.763424	-0.502565
C	-0.879887	-0.338622	-1.100506
C	0.174417	-1.434724	-1.406612
C	-1.973883	-0.863980	-0.179658
O	-2.963374	-1.427060	-0.592423
O	-1.686278	-0.692074	1.111329
C	-0.733211	1.972913	-0.355247
O	-1.922805	2.072819	-0.659178
C	-2.647760	-1.199451	2.061298
H	2.696633	-2.675943	-0.755817
H	2.036056	1.905048	1.078778
H	4.481452	-1.581998	0.597907
H	4.120083	0.698179	1.496431
H	-1.377861	0.011683	-2.001014
H	-0.173154	-2.430090	-1.125581
H	0.389735	-1.459183	-2.477742
H	-2.757029	-2.276993	1.945271
H	-2.241121	-0.958880	3.039167
H	-3.610173	-0.710755	1.915466
C	0.051663	3.152314	0.155184
H	1.021702	3.245512	-0.334571
H	0.219320	3.066357	1.231671
H	-0.533635	4.051108	-0.027768

Acetonitrile Trans 2
SCF Energy: -745.815363208 A.U.
Free Energy: -745.624399 A.U.

Atom	Coordinates (Angstroms) X	Y	Z
C	2.299188	-2.048555	-0.339690
C	2.458984	0.607379	0.584124
C	3.453540	-1.597092	0.307790
C	1.241769	-1.173772	-0.527132
C	1.321681	0.152096	-0.079670
C	3.522353	-0.282764	0.764202
N	0.106396	0.819510	-0.380973
C	-0.900487	-0.151076	-0.835152
C	-0.085820	-1.420882	-1.194248
C	-1.906404	-0.435300	0.273938
O	-1.707431	-0.274536	1.455134
O	-3.030742	-0.941688	-0.243200
C	-0.269548	2.134317	-0.284321
O	-1.440321	2.443993	-0.508541
C	-4.053505	-1.338605	0.696008
H	2.228130	-3.073329	-0.688965
H	2.539871	1.609180	0.975363
H	4.288126	-2.271317	0.463383
H	4.410869	0.063799	1.280472
H	-1.437207	0.248126	-1.691727
H	-0.572610	-2.334075	-0.848956
H	0.023361	-1.502628	-2.278653
H	-4.379552	-0.478637	1.279572
H	-4.869610	-1.720690	0.089827
H	-3.672768	-2.114284	1.359422
C	0.765394	3.170284	0.068699
H	1.694066	3.032741	-0.486351
H	0.996945	3.135649	1.136189
H	0.350696	4.150575	-0.157200

Acetonitrile Cis 1
SCF Energy: -745.815009318 A.U.
Free Energy: -745.624758 A.U.

Atom	Coordinates (Angstroms) X	Y	Z
C	2.639470	-1.513658	-0.420683
C	2.110403	1.027696	0.690643
C	3.593702	-0.879719	0.382841
C	1.432645	-0.877617	-0.661663
C	1.170374	0.380964	-0.108132
C	3.325593	0.375865	0.926363
N	-0.130745	0.801687	-0.486008
C	-0.852693	-0.327206	-1.098210
C	0.269718	-1.316536	-1.512834
C	-1.834866	-1.013978	-0.149226
O	-2.666153	-1.801279	-0.542951
O	-1.653972	-0.677427	1.126319
C	-0.688602	2.029715	-0.239111
O	-0.063563	2.922898	0.327140
C	-2.526222	-1.306911	2.091827
H	2.840865	-2.489446	-0.850223
H	1.904327	1.998216	1.113453
H	4.541589	-1.366769	0.583180
H	4.068764	0.861855	1.549397
H	-1.426006	-0.002405	-1.963149
H	-0.024147	-2.354376	-1.355355
H	0.495776	-1.198949	-2.576028
H	-2.394249	-2.387547	2.064534
H	-2.224169	-0.906641	3.054857
H	-3.563281	-1.052746	1.877446
C	-2.110330	2.231302	-0.715157
H	-2.798457	1.517617	-0.256396
H	-2.189629	2.118786	-1.799105
H	-2.421814	3.237562	-0.444569

Acetonitrile Cis 2
SCF Energy: -745.815575610 A.U.
Free Energy: -745.625205 A.U.

Atom	Coordinates (Angstroms) X	Y	Z
C	-2.346294	-1.965295	0.374017
C	-2.460536	0.690298	-0.578782
C	-3.487232	-1.499494	-0.288578
C	-1.274595	-1.106151	0.554547
C	-1.330937	0.209039	0.079233
C	-3.536442	-0.186312	-0.754421
N	-0.111123	0.871174	0.373005
C	0.880299	-0.110197	0.839983
C	0.034787	-1.342990	1.260769
C	1.848917	-0.482190	-0.282339
O	1.694451	-0.222470	-1.452165
O	2.885295	-1.160963	0.216023
C	0.155953	2.202009	0.176633
O	-0.691681	2.973625	-0.265124
C	3.866115	-1.637576	-0.732839
H	-2.300400	-2.984669	0.742820
H	-2.500310	1.705160	-0.941572
H	-4.332650	-2.161369	-0.440310
H	-4.423195	0.168880	-1.268408
H	1.452880	0.277013	1.679330
H	0.516104	-2.281448	0.985101
H	-0.098586	-1.351731	2.345704
H	4.311053	-0.796959	-1.263417
H	4.613540	-2.154783	-0.138748
H	3.398881	-2.319929	-1.441587
C	1.543470	2.674178	0.552230
H	2.318119	2.150944	-0.012500
H	1.744602	2.519620	1.615150
H	1.612268	3.737595	0.334817

Benzene Trans 1
SCF Energy: -745.806869213 A.U.
Free Energy: -745.616142 A.U.

Atom	Coordinates (Angstroms) X	Y	Z
C	2.560045	-1.653573	-0.356308
C	2.125236	0.926749	0.680510
C	3.538072	-1.038801	0.431423
C	1.382356	-0.977421	-0.625379
C	1.164698	0.312538	-0.120119
C	3.312761	0.236656	0.941924
N	-0.116372	0.760928	-0.525647
C	-0.878615	-0.357073	-1.104590
C	0.198955	-1.416783	-1.447974
C	-1.929694	-0.925442	-0.156290
O	-2.832058	-1.632869	-0.537017
O	-1.707437	-0.603727	1.121984
C	-0.754581	1.972729	-0.398973
O	-1.933615	2.068662	-0.721232
C	-2.656871	-1.113025	2.078439
H	2.718960	-2.651920	-0.751128
H	1.976847	1.902480	1.115788
H	4.464053	-1.557719	0.652145
H	4.064331	0.708627	1.565531
H	-1.418588	-0.016585	-1.984671
H	-0.136469	-2.429358	-1.219638
H	0.429051	-1.386060	-2.516879
H	-2.670206	-2.202400	2.054547
H	-2.314101	-0.754034	3.044987
H	-3.652187	-0.729539	1.856530
C	0.024690	3.158377	0.112426
H	1.009036	3.239538	-0.350929
H	0.160533	3.089967	1.194979
H	-0.554080	4.054407	-0.102138

Benzene Trans 2
SCF Energy: -745.807250210 A.U.
Free Energy: -745.616141 A.U.

Atom	Coordinates (Angstroms)		
	X	Y	Z
C	2.321327	-2.032249	-0.322405
C	2.455302	0.634082	0.568002
C	3.470838	-1.563530	0.320478
C	1.256368	-1.170430	-0.521088
C	1.320888	0.161765	-0.088708
C	3.526287	-0.243632	0.759703
N	0.103519	0.813567	-0.404439
C	-0.894262	-0.170936	-0.845787
C	-0.067287	-1.435968	-1.190218
C	-1.893137	-0.447688	0.273230
O	-1.696006	-0.261490	1.447580
O	-3.013246	-0.984132	-0.234203
C	-0.303717	2.123417	-0.297355
O	-1.469605	2.412105	-0.544775
C	-4.034896	-1.339710	0.717925
H	2.260444	-3.062115	-0.659393
H	2.526476	1.641226	0.947203
H	4.311228	-2.228226	0.485802
H	4.411043	0.117283	1.272953
H	-1.440608	0.214613	-1.702734
H	-0.549048	-2.349329	-0.837341
H	0.046931	-1.528890	-2.273897
H	-4.359720	-0.455707	1.265676
H	-4.851724	-1.746791	0.128206
H	-3.656916	-2.084624	1.418058
C	0.704601	3.172517	0.097525
H	1.637967	3.079673	-0.459998
H	0.933785	3.102254	1.163841
H	0.262462	4.147980	-0.093931

Benzene Cis 1
SCF Energy: -745.807125801 A.U.
Free Energy: -745.616809 A.U.

Atom	Coordinates (Angstroms) X	Y	Z
C	2.642206	-1.511799	-0.404489
C	2.107861	1.044238	0.671117
C	3.593599	-0.865771	0.391636
C	1.435206	-0.880621	-0.655309
C	1.170064	0.384152	-0.118338
C	3.322627	0.396834	0.916291
N	-0.129395	0.799729	-0.505207
C	-0.849163	-0.335897	-1.102224
C	0.274946	-1.327793	-1.506850
C	-1.829595	-1.018051	-0.146886
O	-2.657332	-1.811568	-0.529483
O	-1.647529	-0.665503	1.127056
C	-0.699485	2.023303	-0.240384
O	-0.088465	2.912939	0.335232
C	-2.508305	-1.297965	2.096504
H	2.846750	-2.492927	-0.821085
H	1.897697	2.021242	1.076753
H	4.542159	-1.348296	0.600125
H	4.064987	0.892970	1.532401
H	-1.426678	-0.023822	-1.969822
H	-0.022766	-2.364157	-1.344695
H	0.504372	-1.217789	-2.570873
H	-2.364864	-2.377716	2.076542
H	-2.209888	-0.886835	3.056467
H	-3.550459	-1.063094	1.882466
C	-2.123458	2.215117	-0.721609
H	-2.807917	1.493341	-0.269551
H	-2.199492	2.109793	-1.806948
H	-2.440557	3.217755	-0.444527

Benzene Cis 2
SCF Energy: -745.806844963 A.U.
Free Energy: -745.616584 A.U.

Atom	Coordinates (Angstroms) X	Y	Z
C	-2.341002	-1.975077	0.359980
C	-2.462750	0.687893	-0.571994
C	-3.482573	-1.507388	-0.298549
C	-1.272314	-1.113965	0.546291
C	-1.332278	0.204132	0.081434
C	-3.534645	-0.190843	-0.753646
N	-0.116466	0.869250	0.381678
C	0.877754	-0.109422	0.839644
C	0.038132	-1.349765	1.251884
C	1.856083	-0.466902	-0.279880
O	1.736970	-0.164879	-1.439752
O	2.872064	-1.188791	0.218424
C	0.144521	2.205514	0.179231
O	-0.704028	2.971910	-0.254019
C	3.855480	-1.647631	-0.732444
H	-2.292850	-2.997575	0.720830
H	-2.502419	1.706120	-0.925381
H	-4.326302	-2.170263	-0.455983
H	-4.422424	0.165661	-1.265071
H	1.449370	0.273548	1.682959
H	0.527645	-2.282511	0.970265
H	-0.095601	-1.368006	2.337404
H	4.324084	-0.798296	-1.228488
H	4.585100	-2.202055	-0.148897
H	3.386994	-2.291592	-1.476059
C	1.535808	2.680458	0.546642
H	2.303593	2.173398	-0.041898
H	1.754589	2.511035	1.604294
H	1.590137	3.747820	0.345494

Chloroform Trans 1
SCF Energy: -745.810718771 A.U.
Free Energy: -745.619911 A.U.

Atom	Coordinates (Angstroms) X	Y	Z
C	2.557047	-1.663673	-0.360354
C	2.142485	0.924063	0.668055
C	3.544958	-1.048853	0.415042
C	1.379281	-0.983750	-0.621210
C	1.172053	0.309075	-0.120240
C	3.329737	0.230280	0.921642
N	-0.111671	0.761640	-0.515964
C	-0.879803	-0.349276	-1.102536
C	0.187344	-1.425894	-1.429135
C	-1.950227	-0.898474	-0.166166
O	-2.891187	-1.547176	-0.562100
O	-1.698843	-0.637793	1.119016
C	-0.743515	1.973285	-0.381553
O	-1.927057	2.072212	-0.696421
C	-2.653514	-1.145185	2.073531
H	2.707892	-2.664377	-0.751982
H	2.003567	1.902837	1.099219
H	4.471020	-1.570354	0.629137
H	4.089090	0.702612	1.535310
H	-1.401178	-0.004734	-1.992067
H	-0.153815	-2.431009	-1.177030
H	0.411040	-1.420197	-2.499353
H	-2.707455	-2.231538	2.011077
H	-2.282052	-0.834708	3.045980
H	-3.636146	-0.715664	1.882450
C	0.039353	3.155652	0.128767
H	1.017487	3.240849	-0.346521
H	0.189228	3.079903	1.208817
H	-0.541480	4.053452	-0.072313

Chloroform Trans 2
SCF Energy: -745.811127579 A.U.
Free Energy: -745.620058 A.U.

Atom	Coordinates (Angstroms) X	Y	Z
C	2.313014	-2.038868	-0.330538
C	2.457143	0.622641	0.575682
C	3.464713	-1.577293	0.314016
C	1.250723	-1.171874	-0.523449
C	1.321509	0.157453	-0.083995
C	3.525457	-0.259973	0.761450
N	0.104497	0.815623	-0.392783
C	-0.896838	-0.163055	-0.840269
C	-0.074965	-1.430161	-1.190730
C	-1.899628	-0.442884	0.273775
O	-1.701985	-0.268017	1.451392
O	-3.021584	-0.965316	-0.238491
C	-0.288697	2.127828	-0.292119
O	-1.456664	2.425621	-0.531176
C	-4.045152	-1.337690	0.707632
H	2.248094	-3.066441	-0.673222
H	2.532037	1.627206	0.960963
H	4.302974	-2.245841	0.474494
H	4.411774	0.094875	1.276063
H	-1.438462	0.228279	-1.697494
H	-0.558228	-2.343438	-0.840172
H	0.036238	-1.518984	-2.274753
H	-4.368530	-0.463795	1.271857
H	-4.862332	-1.731252	0.109780
H	-3.667959	-2.098022	1.390995
C	0.731075	3.171378	0.084907
H	1.662716	3.058985	-0.471462
H	0.960726	3.116419	1.151963
H	0.301223	4.149329	-0.121498

Chloroform Cis 1
SCF Energy: -745.810926774 A.U.
Free Energy: -745.620596 A.U.

Atom	X	Y	Z
C	2.639626	-1.513635	-0.414578
C	2.110737	1.035514	0.679317
C	3.593716	-0.874206	0.384173
C	1.432896	-0.879189	-0.659199
C	1.170543	0.382609	-0.113647
C	3.325535	0.385147	0.918423
N	-0.129792	0.801720	-0.494657
C	-0.851833	-0.329208	-1.100382
C	0.270255	-1.321600	-1.509479
C	-1.834828	-1.012680	-0.149338
O	-2.671263	-1.794966	-0.539351
O	-1.645597	-0.677878	1.126731
C	-0.693563	2.027268	-0.236437
O	-0.075097	2.918307	0.334338
C	-2.511102	-1.310197	2.094264
H	2.841496	-2.492304	-0.837826
H	1.903691	2.009515	1.093545
H	4.541886	-1.359556	0.587591
H	4.069308	0.875888	1.537073
H	-1.425952	-0.009438	-1.967027
H	-0.026645	-2.358233	-1.348424
H	0.497463	-1.209422	-2.573388
H	-2.379458	-2.390966	2.063124
H	-2.204107	-0.912196	3.056840
H	-3.550380	-1.058773	1.886430
C	-2.117338	2.223690	-0.712696
H	-2.802334	1.504186	-0.258077
H	-2.196420	2.117325	-1.797516
H	-2.432214	3.227148	-0.436016

Chloroform Cis 2
SCF Energy: -745.810946250 A.U.
Free Energy: -745.620571 A.U.

Atom	Coordinates (Angstroms) X	Y	Z
C	-2.346627	-1.969740	0.365941
C	-2.463165	0.690266	-0.574482
C	-3.488442	-1.501286	-0.292359
C	-1.275226	-1.110886	0.548185
C	-1.332782	0.206160	0.079376
C	-3.538243	-0.186100	-0.752021
N	-0.114172	0.868615	0.375475
C	0.878226	-0.111722	0.837897
C	0.035822	-1.349754	1.251133
C	1.854024	-0.474484	-0.281581
O	1.717280	-0.196204	-1.447145
O	2.882491	-1.170644	0.219413
C	0.150296	2.202515	0.178326
O	-0.697900	2.973109	-0.255650
C	3.865801	-1.638418	-0.729370
H	-2.300052	-2.991040	0.729865
H	-2.502044	1.706963	-0.932241
H	-4.333995	-2.162638	-0.446085
H	-4.425792	0.171001	-1.263365
H	1.448399	0.272395	1.681030
H	0.520525	-2.284419	0.967848
H	-0.095676	-1.367792	2.336529
H	4.319944	-0.793596	-1.245683
H	4.606131	-2.170281	-0.138841
H	3.399562	-2.305304	-1.453644
C	1.540671	2.674213	0.549180
H	2.311448	2.156612	-0.026016
H	1.749449	2.514015	1.610087
H	1.604181	3.739124	0.337902

Dichloromethane Trans 1
SCF Energy: -745.812837358 A.U.
Free Energy: -745.622072 A.U.

Atom	Coordinates (Angstroms) X	Y	Z
C	2.556709	-1.668830	-0.361976
C	2.153639	0.923886	0.659511
C	3.550625	-1.052999	0.404964
C	1.378478	-0.987404	-0.617767
C	1.177138	0.307405	-0.120266
C	3.341129	0.228602	0.908273
N	-0.108527	0.761556	-0.509665
C	-0.879471	-0.345418	-1.101098
C	0.181269	-1.432430	-1.415829
C	-1.962465	-0.881665	-0.172665
O	-2.927108	-1.490234	-0.577499
O	-1.694230	-0.661413	1.115882
C	-0.737933	1.972354	-0.370227
O	-1.924310	2.071668	-0.680387
C	-2.653347	-1.166774	2.068224
H	2.702952	-2.671139	-0.751009
H	2.020235	1.904789	1.087344
H	4.477074	-1.575461	0.614933
H	4.105253	0.701988	1.515079
H	-1.388614	0.001147	-1.996851
H	-0.163198	-2.432329	-1.147638
H	0.400804	-1.443607	-2.486584
H	-2.732473	-2.249937	1.983195
H	-2.266465	-0.886108	3.043566
H	-3.626906	-0.709952	1.895338
C	0.045765	3.153590	0.139585
H	1.019736	3.242789	-0.343277
H	0.204839	3.073149	1.217881
H	-0.537580	4.051695	-0.052663

Dichloromethane Trans 2
SCF Energy: -745.813267379 A.U.
Free Energy: -745.622241 A.U.

Atom	Coordinates (Angstroms) X	Y	Z
C	2.306828	-2.043349	-0.334855
C	2.458118	0.615593	0.579686
C	3.459803	-1.586382	0.310948
C	1.246687	-1.172800	-0.525056
C	1.321693	0.154902	-0.081845
C	3.524237	-0.270448	0.762645
N	0.105361	0.817345	-0.387070
C	-0.898515	-0.157658	-0.837702
C	-0.079972	-1.426072	-1.192043
C	-1.902945	-0.439414	0.273891
O	-1.704692	-0.271343	1.453259
O	-3.026132	-0.953884	-0.240818
C	-0.279672	2.130738	-0.288472
O	-1.449107	2.434036	-0.520588
C	-4.049585	-1.337821	0.701902
H	2.239068	-3.069621	-0.680611
H	2.535717	1.618848	0.967836
H	4.296431	-2.257510	0.469051
H	4.411581	0.080592	1.278003
H	-1.437691	0.237226	-1.694781
H	-0.564728	-2.339282	-0.843665
H	0.030086	-1.511817	-2.276236
H	-4.373670	-0.470624	1.275858
H	-4.866512	-1.725071	0.099861
H	-3.671201	-2.106178	1.375342
C	0.747187	3.170911	0.077062
H	1.677549	3.046533	-0.478587
H	0.977557	3.125790	1.144412
H	0.324540	4.150018	-0.138772

Dichloromethane Cis 1
SCF Energy: -745.813000939 A.U.
Free Energy: -745.622683 A.U.

Atom	Coordinates (Angstroms) X	Y	Z
C	2.639076	-1.513962	-0.418711
C	2.111111	1.031123	0.684788
C	3.593738	-0.877672	0.382160
C	1.432324	-0.878396	-0.660804
C	1.170527	0.381713	-0.110851
C	3.326075	0.379717	0.921489
N	-0.130212	0.802077	-0.489652
C	-0.852679	-0.327188	-1.099276
C	0.269108	-1.318679	-1.511111
C	-1.835897	-1.012052	-0.149781
O	-2.672291	-1.793812	-0.542460
O	-1.648021	-0.680136	1.126537
C	-0.690716	2.028878	-0.236476
O	-0.068590	2.920854	0.331865
C	-2.516584	-1.311606	2.092995
H	2.840281	-2.491150	-0.845437
H	1.904906	2.003307	1.103670
H	4.541771	-1.364102	0.583477
H	4.069909	0.867796	1.542141
H	-1.425869	-0.004221	-1.965162
H	-0.026674	-2.355760	-1.351440
H	0.495476	-1.204320	-2.574770
H	-2.387174	-2.392514	2.060787
H	-2.209495	-0.915578	3.056257
H	-3.554581	-1.056059	1.884362
C	-2.113697	2.228066	-0.711856
H	-2.799914	1.510395	-0.256340
H	-2.192986	2.120450	-1.796434
H	-2.427253	3.232326	-0.436473

Dichloromethane Cis 2
SCF Energy: -745.813264579 A.U.
Free Energy: -745.622871 A.U.

Atom	Coordinates (Angstroms) X	Y	Z
C	-2.347302	-1.967430	0.369768
C	-2.462399	0.690516	-0.576444
C	-3.488891	-1.499875	-0.289993
C	-1.275415	-1.108759	0.550699
C	-1.332270	0.207433	0.078925
C	-3.538229	-0.185623	-0.752615
N	-0.112785	0.869416	0.373450
C	0.879051	-0.111481	0.838268
C	0.035183	-1.347348	1.254347
C	1.852014	-0.478400	-0.282094
O	1.706182	-0.210786	-1.450114
O	2.885370	-1.163950	0.218472
C	0.152953	2.201898	0.177638
O	-0.695219	2.973542	-0.258806
C	3.867912	-1.636375	-0.729880
H	-2.301155	-2.987836	0.735952
H	-2.501585	1.706311	-0.936704
H	-4.334640	-2.161234	-0.442396
H	-4.425499	0.170820	-1.264862
H	1.449691	0.274037	1.680084
H	0.517922	-2.283642	0.973428
H	-0.096615	-1.362108	2.339559
H	4.316492	-0.793836	-1.254483
H	4.612423	-2.159335	-0.136913
H	3.401776	-2.312476	-1.445446
C	1.542265	2.673281	0.550251
H	2.314610	2.152262	-0.019681
H	1.747293	2.516322	1.612230
H	1.608882	3.737326	0.335445

Dmso Trans 1
SCF Energy: -745.815086624 A.U.
Free Energy: -745.624436 A.U.

Atom	Coordinates (Angstroms) X	Y	Z
C	2.554868	-1.673069	-0.368677
C	2.164693	0.922361	0.652245
C	3.555117	-1.058605	0.391170
C	1.376510	-0.988901	-0.617582
C	1.181838	0.306740	-0.120363
C	3.352070	0.224349	0.894312
N	-0.105888	0.763828	-0.501562
C	-0.880083	-0.337503	-1.100443
C	0.173579	-1.434340	-1.406301
C	-1.974979	-0.862189	-0.180351
O	-2.966901	-1.420576	-0.593851
O	-1.685306	-0.695641	1.110815
C	-0.732611	1.973317	-0.353465
O	-1.922502	2.073501	-0.656706
C	-2.646863	-1.203687	2.060423
H	2.695870	-2.676111	-0.757502
H	2.037262	1.904364	1.079158
H	4.481665	-1.583029	0.595667
H	4.121215	0.696896	1.495311
H	-1.377131	0.013510	-2.001185
H	-0.174287	-2.429381	-1.124425
H	0.388374	-1.459643	-2.477492
H	-2.759750	-2.280402	1.940314
H	-2.237835	-0.968286	3.038538
H	-3.607933	-0.711296	1.918177
C	0.052639	3.152402	0.156962
H	1.022579	3.245412	-0.333010
H	0.220602	3.066199	1.233379
H	-0.532402	4.051434	-0.025640

Dmso Trans 2
SCF Energy: -745.815544475 A.U.
Free Energy: -745.624582 A.U.

Atom	Coordinates (Angstroms) X	Y	Z
C	2.298073	-2.049206	-0.340557
C	2.458976	0.606124	0.584945
C	3.452562	-1.598631	0.307331
C	1.241065	-1.173815	-0.527594
C	1.321613	0.151749	-0.079329
C	3.521953	-0.284612	0.764596
N	0.106526	0.819857	-0.380093
C	-0.900747	-0.150070	-0.834847
C	-0.086596	-1.419954	-1.194839
C	-1.906825	-0.434801	0.273963
O	-1.707786	-0.274652	1.455360
O	-3.031114	-0.940661	-0.243366
C	-0.268203	2.134929	-0.283912
O	-1.438935	2.445513	-0.507748
C	-4.053849	-1.338846	0.695569
H	2.226611	-3.073729	-0.690474
H	2.540296	1.607651	0.976799
H	4.286824	-2.273342	0.462561
H	4.410580	0.061240	1.281149
H	-1.437255	0.249845	-1.691229
H	-0.573697	-2.333234	-0.850226
H	0.022530	-1.500904	-2.279304
H	-4.379963	-0.479565	1.280085
H	-4.869911	-1.720352	0.088991
H	-3.672838	-2.115273	1.357918
C	0.767697	3.170233	0.068077
H	1.696254	3.031200	-0.486780
H	0.999211	3.136533	1.135609
H	0.354042	4.150733	-0.158839

Dmso Cis 1
SCF Energy: -745.815181680 A.U.
Free Energy: -745.624939 A.U.

Atom	Coordinates (Angstroms) X	Y	Z
C	2.639619	-1.513576	-0.420565
C	2.110240	1.027532	0.691161
C	3.593698	-0.879730	0.383234
C	1.432784	-0.877574	-0.661628
C	1.170369	0.380916	-0.107917
C	3.325451	0.375739	0.926983
N	-0.130761	0.801567	-0.485897
C	-0.852567	-0.327450	-1.098132
C	0.269992	-1.316471	-1.512974
C	-1.834554	-1.014392	-0.149047
O	-2.664832	-1.802871	-0.542664
O	-1.654936	-0.676581	1.126301
C	-0.688491	2.029667	-0.239649
O	-0.063367	2.922970	0.326583
C	-2.527594	-1.305739	2.091755
H	2.841125	-2.489267	-0.850248
H	1.904084	1.997937	1.114207
H	4.541575	-1.366777	0.583619
H	4.068479	0.861629	1.550261
H	-1.426009	-0.002648	-1.962976
H	-0.023629	-2.354404	-1.355747
H	0.496103	-1.198523	-2.576104
H	-2.394774	-2.386293	2.065682
H	-2.226672	-0.904289	3.054645
H	-3.564609	-1.052421	1.876206
C	-2.110014	2.231372	-0.716056
H	-2.798413	1.518297	-0.256753
H	-2.189200	2.117935	-1.799905
H	-2.421257	3.237952	-0.446365

Dmso Cis 2
SCF Energy: -745.815777337 A.U.
Free Energy: -745.625416 A.U.

Atom	Coordinates (Angstroms) X	Y	Z
C	-2.345923	-1.965279	0.374539
C	-2.460415	0.690024	-0.579040
C	-3.486807	-1.499847	-0.288436
C	-1.274386	-1.105912	0.555054
C	-1.330844	0.209113	0.079283
C	-3.536189	-0.186789	-0.754661
N	-0.111011	0.871400	0.372768
C	0.880487	-0.109861	0.840061
C	0.034939	-1.342386	1.261504
C	1.848694	-0.482591	-0.282339
O	1.693063	-0.224468	-1.452483
O	2.885721	-1.160002	0.215999
C	0.155812	2.202183	0.176554
O	-0.692148	2.973685	-0.265161
C	3.866319	-1.637336	-0.732871
H	-2.299962	-2.984528	0.743651
H	-2.500427	1.704804	-0.942051
H	-4.332080	-2.161913	-0.440133
H	-4.422926	0.168114	-1.268859
H	1.453188	0.277705	1.679117
H	0.516133	-2.281053	0.986389
H	-0.098537	-1.350447	2.346419
H	4.310614	-0.797075	-1.264514
H	4.614258	-2.153508	-0.138540
H	3.398968	-2.320779	-1.440460
C	1.543317	2.674610	0.551624
H	2.317743	2.151755	-0.013770
H	1.745173	2.519701	1.614334
H	1.611807	3.738126	0.334568

Methanol Trans 1
SCF Energy: -745.814837578 A.U.
Free Energy: -745.624163 A.U.

Atom	Coordinates (Angstroms) X	Y	Z
C	2.555206	-1.672641	-0.367295
C	2.163478	0.922854	0.652717
C	3.554701	-1.057718	0.393170
C	1.376861	-0.988896	-0.617272
C	1.181353	0.306812	-0.120452
C	3.350892	0.225273	0.895830
N	-0.106162	0.763364	-0.502761
C	-0.879894	-0.338834	-1.100514
C	0.174671	-1.434548	-1.407056
C	-1.973441	-0.864688	-0.179385
O	-2.961946	-1.429655	-0.591894
O	-1.686601	-0.690812	1.111527
C	-0.733363	1.972931	-0.355814
O	-1.922853	2.072823	-0.659942
C	-2.647942	-1.198190	2.061621
H	2.696857	-2.675730	-0.755788
H	2.035399	1.904916	1.079324
H	4.481240	-1.581831	0.598520
H	4.119460	0.698168	1.497298
H	-1.378321	0.011378	-2.000810
H	-0.172745	-2.430137	-1.126632
H	0.390146	-1.458362	-2.478182
H	-2.755758	-2.276028	1.946952
H	-2.242145	-0.955803	3.039396
H	-3.610911	-0.710951	1.914600
C	0.051515	3.152418	0.154465
H	1.021781	3.245264	-0.334920
H	0.218686	3.066866	1.231062
H	-0.533580	4.051219	-0.029098

Methanol Trans 2
SCF Energy: -745.815292087 A.U.
Free Energy: -745.624326 A.U.

Atom	Coordinates (Angstroms) X	Y	Z
C	2.299260	-2.048452	-0.339819
C	2.458909	0.607365	0.584255
C	3.453572	-1.597019	0.307753
C	1.241818	-1.173703	-0.527225
C	1.321654	0.152138	-0.079626
C	3.522317	-0.282746	0.764298
N	0.106373	0.819502	-0.380919
C	-0.900460	-0.151088	-0.835160
C	-0.085745	-1.420811	-1.194362
C	-1.906382	-0.435407	0.273952
O	-1.707429	-0.274385	1.455119
O	-3.030592	-0.942142	-0.243097
C	-0.269633	2.134352	-0.284487
O	-1.440252	2.443990	-0.509292
C	-4.053457	-1.338605	0.696265
H	2.228278	-3.073204	-0.689190
H	2.539741	1.609121	0.975618
H	4.288165	-2.271247	0.463318
H	4.410792	0.063797	1.280662
H	-1.437247	0.248162	-1.691683
H	-0.572505	-2.334061	-0.849150
H	0.023433	-1.502473	-2.278787
H	-4.379240	-0.478394	1.279607
H	-4.869665	-1.720651	0.090192
H	-3.672850	-2.114189	1.359865
C	0.765131	3.170305	0.069122
H	1.693964	3.032981	-0.485737
H	0.996406	3.135356	1.136665
H	0.350407	4.150624	-0.156592

Methanol Cis 1
SCF Energy: -745.814941570 A.U.
Free Energy: -745.624688 A.U.

Atom	Coordinates (Angstroms) X	Y	Z
C	2.639409	-1.513714	-0.420672
C	2.110479	1.027762	0.690440
C	3.593688	-0.879739	0.382753
C	1.432601	-0.877644	-0.661659
C	1.170393	0.380982	-0.108216
C	3.325648	0.375908	0.926161
N	-0.130716	0.801736	-0.486084
C	-0.852722	-0.327126	-1.098245
C	0.269631	-1.316570	-1.512780
C	-1.834969	-1.013831	-0.149285
O	-2.666547	-1.800794	-0.543036
O	-1.653717	-0.677647	1.126317
C	-0.688616	2.029739	-0.238954
O	-0.063617	2.922867	0.327326
C	-2.525833	-1.307265	2.091834
H	2.840767	-2.489547	-0.850135
H	1.904448	1.998342	1.113129
H	4.541570	-1.366803	0.583099
H	4.068873	0.861932	1.549102
H	-1.426003	-0.002333	-1.963210
H	-0.024323	-2.354372	-1.355204
H	0.495686	-1.199111	-2.575995
H	-2.394035	-2.387915	2.064202
H	-2.223488	-0.907306	3.054904
H	-3.562915	-1.052916	1.877774
C	-2.110424	2.231269	-0.714862
H	-2.798455	1.517514	-0.256062
H	-2.189859	2.118845	-1.798812
H	-2.421920	3.237491	-0.444150

Methanol Cis 2
SCF Energy: -745.815496447 A.U.
Free Energy: -745.625124 A.U.

Atom	Coordinates (Angstroms) X	Y	Z
C	-2.346262	-1.965400	0.373890
C	-2.460570	0.690253	-0.578742
C	-3.487223	-1.499571	-0.288629
C	-1.274571	-1.106251	0.554423
C	-1.330955	0.208964	0.079209
C	-3.536456	-0.186363	-0.754393
N	-0.111187	0.871137	0.373046
C	0.880270	-0.110192	0.839948
C	0.034835	-1.343094	1.260608
C	1.849030	-0.481998	-0.282324
O	1.694958	-0.221800	-1.452057
O	2.885184	-1.161228	0.216063
C	0.155818	2.202025	0.176654
O	-0.691838	2.973597	-0.264991
C	3.866069	-1.637659	-0.732802
H	-2.300347	-2.984799	0.742629
H	-2.500331	1.705137	-0.941476
H	-4.332639	-2.161446	-0.440380
H	-4.423226	0.168843	-1.268342
H	1.452793	0.276990	1.679362
H	0.516237	-2.281478	0.984824
H	-0.098529	-1.351994	2.345549
H	4.311263	-0.796945	-1.263016
H	4.613297	-2.155245	-0.138785
H	3.398834	-2.319632	-1.441919
C	1.543347	2.674231	0.552255
H	2.318016	2.150943	-0.012395
H	1.744416	2.519826	1.615217
H	1.612117	3.737622	0.334711

35. Computed relative free energies (in kcal/mol) of the various conformers of Ac-(2*S*)-Ind-(2*S*)-Ind-OMe in chloroform and DMSO.

We report the relative free energy (in kcal/mol) of the fifteen stable conformers of Ac-(2S)-Ind-(2S)-Ind-OMe. The most stable conformer is used as a reference. All the data have been obtained using the B3LYP DFT functional augmented with Grimme's D3 dispersion corrections in conjunction with the 6-311+G(d) basis set. Solvation effects have been introduced using the integral equation formalism polarizable continuum model (IEFPCM). All the calculations have been performed using Gaussian 16. The absolute values are reported together with the atomic coordinates of all the structures in the following. The ψ dihedrals specifying the conformations are reported in section 36.

Conformer	Chloroform	DMSO
TT1	0.64	0.88
TT2	0.28	1.24
CT1	0.35	0.74
TC1	0.31	0.55
CT2	0.34	0.06
CC	0.07	0.14
TT2	2.50	3.32
CT3	0.00	1.13
CC2	0.95	1.87
CC3	0.24	0.00
TC2	2.28	2.98
TC3	1.64	1.34
TC4	1.81	2.10
TC5	3.59	1.34
CT4	3.94	5.12

36. Geometries, computed SCF and free energies of all the optimized structures of the conformers of Ac-(2*S*)-Ind-(2*S*)-Ind-OMe in various solvents.

In the following, we report the coordinates, SCF energies and free energies for all structures resulting from a systematic conformational search (15 conformers) in chloroform and dmso. All the calculations were obtained using the B3LYP-D3BJ/6-311+G(d)/IEFPCM (DMSO) level of theory, using the default grid in Gaussian 16 for DFT exchange-correlation integration, corresponding to the grid=ultrafine option (i.e., a pruned 99,590 grid). All structures are minima (i.e., no negative frequencies). In order to better clarify the structure of the various conformers, besides the cis (C) or trans (T) conformation of the two amidic bonds (ω dihedral), we report in parentheses the value of the two ψ dihedrals as well. Energies and free energies are given in atomic units, coordinates in Angstroms. We also report a table listing all the values of the two ψ dihedrals for the various solvents, together with the label used to identify the conformation in the previous section.

Conformer	Chloroform				DMSO			
	ψ_1	ω_1	ψ_2	ω_2	ψ_1	ω_1	ψ_2	ω_2
TT1	-21	177	-21	177	-21	176	-25	177
TT2	-25	177	163	-178	-24	175	163	-176
CT1	-12	5	-21	172	-14	5	-27	171
TC1	-27	177	-40	-9	-29	176	-39	-8
CT2	-22	4	163	179	-20	4	163	-177
CC	-15	5	-13	-8	-14	6	-10	-7
TT2	-40	178	142	154	-40	178	142	155
CT3	143	-1	174	-9	143	-3	173	-8
CC2	150	0	3	-10	151	-3	2	-8
CC3	-14	5	170	-8	-14	5	169	-8
TC2	149	164	-6	10	171	169	1	-4
TC3	-17	177	122	-10	-30	175	159	-6
TC4	143	170	174	-8	146	170	173	-7
TC5	-17	170	-176	-7	-90	175	159	-6
CT4	122	-5	-18	-169	122	5	-28	-169

Chloroform TT(-21-21)
SCF Energy: -1223.14047532 A.U.
Free Energy: -1222.818904 A.U.

Atom	X	Y	Z
C	3.077707	1.388800	2.090532
C	4.342454	0.215932	-0.135583
C	4.362716	1.795020	1.716956
C	2.441031	0.401426	1.358374
C	3.069499	-0.192686	0.255141
C	4.978945	1.210620	0.613309
N	2.193142	-1.146202	-0.318380
C	0.846185	-0.989260	0.249392
C	1.078187	-0.205395	1.567034
C	-0.019871	-0.178163	-0.714680
O	0.484635	0.543929	-1.564979
C	2.363407	-2.075996	-1.313415
O	1.394191	-2.716269	-1.714629
N	-1.380702	-0.234641	-0.580975
C	-2.240697	-0.880289	0.346087
C	-3.571545	-0.677985	-0.046574
C	-3.627078	0.117427	-1.323604
C	-2.169460	0.609619	-1.496569
C	-4.617800	-1.196196	0.697942
C	-4.338656	-1.928173	1.855522
C	-3.016958	-2.119538	2.248289
C	-1.953834	-1.595731	1.506849
C	-2.037130	2.096019	-1.181406
O	-2.326297	2.959050	-1.977116
O	-1.623413	2.326565	0.068042
C	-1.471411	3.709448	0.447481
H	2.581411	1.844148	2.941446
H	4.840252	-0.190153	-1.001977
H	4.872677	2.570106	2.277842
H	5.968688	1.536750	0.312666
H	0.417535	-1.975245	0.399735
H	0.300941	0.539973	1.742568
H	1.081511	-0.884897	2.424594
H	-4.336703	0.945064	-1.283096
H	-3.913496	-0.513184	-2.169269
H	-1.810311	0.479641	-2.514121
H	-5.643332	-1.032420	0.383534
H	-5.147994	-2.338257	2.448831
H	-2.796774	-2.677311	3.151874
H	-0.949822	-1.750026	1.866022
H	-2.426204	4.228564	0.371470
H	-0.735815	4.192881	-0.194202
H	-1.126492	3.689732	1.477446
C	3.745673	-2.305713	-1.868351
H	3.729234	-3.234223	-2.435384
H	4.501555	-2.370916	-1.084574
H	4.024946	-1.495637	-2.546830

Chloroform TT(-24,163)
SCF Energy: -1223.14085996 A.U.
Free Energy: -1222.819479 A.U.

Atom	Coordinates (Angstroms) X	Y	Z
C	3.077707	1.388800	2.090532
C	4.342454	0.215932	-0.135583
C	4.362716	1.795020	1.716956
C	2.441031	0.401426	1.358374
C	3.069499	-0.192686	0.255141
C	4.978945	1.210620	0.613309
N	2.193142	-1.146202	-0.318380
C	0.846185	-0.989260	0.249392
C	1.078187	-0.205395	1.567034
C	-0.019871	-0.178163	-0.714680
O	0.484635	0.543929	-1.564979
C	2.363407	-2.075996	-1.313415
O	1.394191	-2.716269	-1.714629
N	-1.380702	-0.234641	-0.580975
C	-2.240697	-0.880289	0.346087
C	-3.571545	-0.677985	-0.046574
C	-3.627078	0.117427	-1.323604
C	-2.169460	0.609619	-1.496569
C	-4.617800	-1.196196	0.697942
C	-4.338656	-1.928173	1.855522
C	-3.016958	-2.119538	2.248289
C	-1.953834	-1.595731	1.506849
C	-2.037130	2.096019	-1.181406
O	-2.326297	2.959050	-1.977116
O	-1.623413	2.326565	0.068042
C	-1.471411	3.709448	0.447481
H	2.581411	1.844148	2.941446
H	4.840252	-0.190153	-1.001977
H	4.872677	2.570106	2.277842
H	5.968688	1.536750	0.312666
H	0.417535	-1.975245	0.399735
H	0.300941	0.539973	1.742568
H	1.081511	-0.884897	2.424594
H	-4.336703	0.945064	-1.283096
H	-3.913496	-0.513184	-2.169269
H	-1.810311	0.479641	-2.514121
H	-5.643332	-1.032420	0.383534
H	-5.147994	-2.338257	2.448831
H	-2.796774	-2.677311	3.151874
H	-0.949822	-1.750026	1.866022
H	-2.426204	4.228564	0.371470
H	-0.735815	4.192881	-0.194202
H	-1.126492	3.689732	1.477446
C	3.745673	-2.305713	-1.868351
H	3.729234	-3.234223	-2.435384
H	4.501555	-2.370916	-1.084574
H	4.024946	-1.495637	-2.546830

Chloroform TC(-14,-21)
SCF Energy: -1223.14055176 A.U.
Free Energy: -1222.819364 A.U.

Atom	Coordinates (Angstroms) X	Y	Z
C	3.077707	1.388800	2.090532
C	4.342454	0.215932	-0.135583
C	4.362716	1.795020	1.716956
C	2.441031	0.401426	1.358374
C	3.069499	-0.192686	0.255141
C	4.978945	1.210620	0.613309
N	2.193142	-1.146202	-0.318380
C	0.846185	-0.989260	0.249392
C	1.078187	-0.205395	1.567034
C	-0.019871	-0.178163	-0.714680
O	0.484635	0.543929	-1.564979
C	2.363407	-2.075996	-1.313415
O	1.394191	-2.716269	-1.714629
N	-1.380702	-0.234641	-0.580975
C	-2.240697	-0.880289	0.346087
C	-3.571545	-0.677985	-0.046574
C	-3.627078	0.117427	-1.323604
C	-2.169460	0.609619	-1.496569
C	-4.617800	-1.196196	0.697942
C	-4.338656	-1.928173	1.855522
C	-3.016958	-2.119538	2.248289
C	-1.953834	-1.595731	1.506849
C	-2.037130	2.096019	-1.181406
O	-2.326297	2.959050	-1.977116
O	-1.623413	2.326565	0.068042
C	-1.471411	3.709448	0.447481
H	2.581411	1.844148	2.941446
H	4.840252	-0.190153	-1.001977
H	4.872677	2.570106	2.277842
H	5.968688	1.536750	0.312666
H	0.417535	-1.975245	0.399735
H	0.300941	0.539973	1.742568
H	1.081511	-0.884897	2.424594
H	-4.336703	0.945064	-1.283096
H	-3.913496	-0.513184	-2.169269
H	-1.810311	0.479641	-2.514121
H	-5.643332	-1.032420	0.383534
H	-5.147994	-2.338257	2.448831
H	-2.796774	-2.677311	3.151874
H	-0.949822	-1.750026	1.866022
H	-2.426204	4.228564	0.371470
H	-0.735815	4.192881	-0.194202
H	-1.126492	3.689732	1.477446
C	3.745673	-2.305713	-1.868351
H	3.729234	-3.234223	-2.435384
H	4.501555	-2.370916	-1.084574
H	4.024946	-1.495637	-2.546830

Chloroform CT(-29,-40)
SCF Energy: -1223.14135631 A.U.
Free Energy: -1222.819424 A.U.

Atom	X	Y	Z
C	3.077707	1.388800	2.090532
C	4.342454	0.215932	-0.135583
C	4.362716	1.795020	1.716956
C	2.441031	0.401426	1.358374
C	3.069499	-0.192686	0.255141
C	4.978945	1.210620	0.613309
N	2.193142	-1.146202	-0.318380
C	0.846185	-0.989260	0.249392
C	1.078187	-0.205395	1.567034
C	-0.019871	-0.178163	-0.714680
O	0.484635	0.543929	-1.564979
C	2.363407	-2.075996	-1.313415
O	1.394191	-2.716269	-1.714629
N	-1.380702	-0.234641	-0.580975
C	-2.240697	-0.880289	0.346087
C	-3.571545	-0.677985	-0.046574
C	-3.627078	0.117427	-1.323604
C	-2.169460	0.609619	-1.496569
C	-4.617800	-1.196196	0.697942
C	-4.338656	-1.928173	1.855522
C	-3.016958	-2.119538	2.248289
C	-1.953834	-1.595731	1.506849
C	-2.037130	2.096019	-1.181406
O	-2.326297	2.959050	-1.977116
O	-1.623413	2.326565	0.068042
C	-1.471411	3.709448	0.447481
H	2.581411	1.844148	2.941446
H	4.840252	-0.190153	-1.001977
H	4.872677	2.570106	2.277842
H	5.968688	1.536750	0.312666
H	0.417535	-1.975245	0.399735
H	0.300941	0.539973	1.742568
H	1.081511	-0.884897	2.424594
H	-4.336703	0.945064	-1.283096
H	-3.913496	-0.513184	-2.169269
H	-1.810311	0.479641	-2.514121
H	-5.643332	-1.032420	0.383534
H	-5.147994	-2.338257	2.448831
H	-2.796774	-2.677311	3.151874
H	-0.949822	-1.750026	1.866022
H	-2.426204	4.228564	0.371470
H	-0.735815	4.192881	-0.194202
H	-1.126492	3.689732	1.477446
C	3.745673	-2.305713	-1.868351
H	3.729234	-3.234223	-2.435384
H	4.501555	-2.370916	-1.084574
H	4.024946	-1.495637	-2.546830

Chloroform TC(-20,163)
SCF Energy: -1223.14076926 A.U.
Free Energy: -1222.819380 A.U.

Atom	Coordinates (Angstroms) X	Y	Z
C	3.077707	1.388800	2.090532
C	4.342454	0.215932	-0.135583
C	4.362716	1.795020	1.716956
C	2.441031	0.401426	1.358374
C	3.069499	-0.192686	0.255141
C	4.978945	1.210620	0.613309
N	2.193142	-1.146202	-0.318380
C	0.846185	-0.989260	0.249392
C	1.078187	-0.205395	1.567034
C	-0.019871	-0.178163	-0.714680
O	0.484635	0.543929	-1.564979
C	2.363407	-2.075996	-1.313415
O	1.394191	-2.716269	-1.714629
N	-1.380702	-0.234641	-0.580975
C	-2.240697	-0.880289	0.346087
C	-3.571545	-0.677985	-0.046574
C	-3.627078	0.117427	-1.323604
C	-2.169460	0.609619	-1.496569
C	-4.617800	-1.196196	0.697942
C	-4.338656	-1.928173	1.855522
C	-3.016958	-2.119538	2.248289
C	-1.953834	-1.595731	1.506849
C	-2.037130	2.096019	-1.181406
O	-2.326297	2.959050	-1.977116
O	-1.623413	2.326565	0.068042
C	-1.471411	3.709448	0.447481
H	2.581411	1.844148	2.941446
H	4.840252	-0.190153	-1.001977
H	4.872677	2.570106	2.277842
H	5.968688	1.536750	0.312666
H	0.417535	-1.975245	0.399735
H	0.300941	0.539973	1.742568
H	1.081511	-0.884897	2.424594
H	-4.336703	0.945064	-1.283096
H	-3.913496	-0.513184	-2.169269
H	-1.810311	0.479641	-2.514121
H	-5.643332	-1.032420	0.383534
H	-5.147994	-2.338257	2.448831
H	-2.796774	-2.677311	3.151874
H	-0.949822	-1.750026	1.866022
H	-2.426204	4.228564	0.371470
H	-0.735815	4.192881	-0.194202
H	-1.126492	3.689732	1.477446
C	3.745673	-2.305713	-1.868351
H	3.729234	-3.234223	-2.435384
H	4.501555	-2.370916	-1.084574
H	4.024946	-1.495637	-2.546830

Chloroform CC(-14,-13)
SCF Energy: -1223.14139751 A.U.
Free Energy: -1222.819812 A.U.

Atom	Coordinates (Angstroms) X	Y	Z
C	3.077707	1.388800	2.090532
C	4.342454	0.215932	-0.135583
C	4.362716	1.795020	1.716956
C	2.441031	0.401426	1.358374
C	3.069499	-0.192686	0.255141
C	4.978945	1.210620	0.613309
N	2.193142	-1.146202	-0.318380
C	0.846185	-0.989260	0.249392
C	1.078187	-0.205395	1.567034
C	-0.019871	-0.178163	-0.714680
O	0.484635	0.543929	-1.564979
C	2.363407	-2.075996	-1.313415
O	1.394191	-2.716269	-1.714629
N	-1.380702	-0.234641	-0.580975
C	-2.240697	-0.880289	0.346087
C	-3.571545	-0.677985	-0.046574
C	-3.627078	0.117427	-1.323604
C	-2.169460	0.609619	-1.496569
C	-4.617800	-1.196196	0.697942
C	-4.338656	-1.928173	1.855522
C	-3.016958	-2.119538	2.248289
C	-1.953834	-1.595731	1.506849
C	-2.037130	2.096019	-1.181406
O	-2.326297	2.959050	-1.977116
O	-1.623413	2.326565	0.068042
C	-1.471411	3.709448	0.447481
H	2.581411	1.844148	2.941446
H	4.840252	-0.190153	-1.001977
H	4.872677	2.570106	2.277842
H	5.968688	1.536750	0.312666
H	0.417535	-1.975245	0.399735
H	0.300941	0.539973	1.742568
H	1.081511	-0.884897	2.424594
H	-4.336703	0.945064	-1.283096
H	-3.913496	-0.513184	-2.169269
H	-1.810311	0.479641	-2.514121
H	-5.643332	-1.032420	0.383534
H	-5.147994	-2.338257	2.448831
H	-2.796774	-2.677311	3.151874
H	-0.949822	-1.750026	1.866022
H	-2.426204	4.228564	0.371470
H	-0.735815	4.192881	-0.194202
H	-1.126492	3.689732	1.477446
C	3.745673	-2.305713	-1.868351
H	3.729234	-3.234223	-2.435384
H	4.501555	-2.370916	-1.084574
H	4.024946	-1.495637	-2.546830

Chloroform TT(-40,142)
SCF Energy: -1223.13818237 A.U.
Free Energy: -1222.815941 A.U.

Atom	X	Y	Z
C	3.077707	1.388800	2.090532
C	4.342454	0.215932	-0.135583
C	4.362716	1.795020	1.716956
C	2.441031	0.401426	1.358374
C	3.069499	-0.192686	0.255141
C	4.978945	1.210620	0.613309
N	2.193142	-1.146202	-0.318380
C	0.846185	-0.989260	0.249392
C	1.078187	-0.205395	1.567034
C	-0.019871	-0.178163	-0.714680
O	0.484635	0.543929	-1.564979
C	2.363407	-2.075996	-1.313415
O	1.394191	-2.716269	-1.714629
N	-1.380702	-0.234641	-0.580975
C	-2.240697	-0.880289	0.346087
C	-3.571545	-0.677985	-0.046574
C	-3.627078	0.117427	-1.323604
C	-2.169460	0.609619	-1.496569
C	-4.617800	-1.196196	0.697942
C	-4.338656	-1.928173	1.855522
C	-3.016958	-2.119538	2.248289
C	-1.953834	-1.595731	1.506849
C	-2.037130	2.096019	-1.181406
O	-2.326297	2.959050	-1.977116
O	-1.623413	2.326565	0.068042
C	-1.471411	3.709448	0.447481
H	2.581411	1.844148	2.941446
H	4.840252	-0.190153	-1.001977
H	4.872677	2.570106	2.277842
H	5.968688	1.536750	0.312666
H	0.417535	-1.975245	0.399735
H	0.300941	0.539973	1.742568
H	1.081511	-0.884897	2.424594
H	-4.336703	0.945064	-1.283096
H	-3.913496	-0.513184	-2.169269
H	-1.810311	0.479641	-2.514121
H	-5.643332	-1.032420	0.383534
H	-5.147994	-2.338257	2.448831
H	-2.796774	-2.677311	3.151874
H	-0.949822	-1.750026	1.866022
H	-2.426204	4.228564	0.371470
H	-0.735815	4.192881	-0.194202
H	-1.126492	3.689732	1.477446
C	3.745673	-2.305713	-1.868351
H	3.729234	-3.234223	-2.435384
H	4.501555	-2.370916	-1.084574
H	4.024946	-1.495637	-2.546830

Coordinates (Angstroms)

Chloroform CC(144,174)
SCF Energy: -1223.14362501 A.U.
Free Energy: -1222.819919 A.U.

Atom	X	Y	Z
C	3.077707	1.388800	2.090532
C	4.342454	0.215932	-0.135583
C	4.362716	1.795020	1.716956
C	2.441031	0.401426	1.358374
C	3.069499	-0.192686	0.255141
C	4.978945	1.210620	0.613309
N	2.193142	-1.146202	-0.318380
C	0.846185	-0.989260	0.249392
C	1.078187	-0.205395	1.567034
C	-0.019871	-0.178163	-0.714680
O	0.484635	0.543929	-1.564979
C	2.363407	-2.075996	-1.313415
O	1.394191	-2.716269	-1.714629
N	-1.380702	-0.234641	-0.580975
C	-2.240697	-0.880289	0.346087
C	-3.571545	-0.677985	-0.046574
C	-3.627078	0.117427	-1.323604
C	-2.169460	0.609619	-1.496569
C	-4.617800	-1.196196	0.697942
C	-4.338656	-1.928173	1.855522
C	-3.016958	-2.119538	2.248289
C	-1.953834	-1.595731	1.506849
C	-2.037130	2.096019	-1.181406
O	-2.326297	2.959050	-1.977116
O	-1.623413	2.326565	0.068042
C	-1.471411	3.709448	0.447481
H	2.581411	1.844148	2.941446
H	4.840252	-0.190153	-1.001977
H	4.872677	2.570106	2.277842
H	5.968688	1.536750	0.312666
H	0.417535	-1.975245	0.399735
H	0.300941	0.539973	1.742568
H	1.081511	-0.884897	2.424594
H	-4.336703	0.945064	-1.283096
H	-3.913496	-0.513184	-2.169269
H	-1.810311	0.479641	-2.514121
H	-5.643332	-1.032420	0.383534
H	-5.147994	-2.338257	2.448831
H	-2.796774	-2.677311	3.151874
H	-0.949822	-1.750026	1.866022
H	-2.426204	4.228564	0.371470
H	-0.735815	4.192881	-0.194202
H	-1.126492	3.689732	1.477446
C	3.745673	-2.305713	-1.868351
H	3.729234	-3.234223	-2.435384
H	4.501555	-2.370916	-1.084574
H	4.024946	-1.495637	-2.546830

Chloroform CC(151,3)
SCF Energy: -1223.14202625 A.U.
Free Energy: -1222.818402 A.U.

Atom	X	Y	Z
C	3.077707	1.388800	2.090532
C	4.342454	0.215932	-0.135583
C	4.362716	1.795020	1.716956
C	2.441031	0.401426	1.358374
C	3.069499	-0.192686	0.255141
C	4.978945	1.210620	0.613309
N	2.193142	-1.146202	-0.318380
C	0.846185	-0.989260	0.249392
C	1.078187	-0.205395	1.567034
C	-0.019871	-0.178163	-0.714680
O	0.484635	0.543929	-1.564979
C	2.363407	-2.075996	-1.313415
O	1.394191	-2.716269	-1.714629
N	-1.380702	-0.234641	-0.580975
C	-2.240697	-0.880289	0.346087
C	-3.571545	-0.677985	-0.046574
C	-3.627078	0.117427	-1.323604
C	-2.169460	0.609619	-1.496569
C	-4.617800	-1.196196	0.697942
C	-4.338656	-1.928173	1.855522
C	-3.016958	-2.119538	2.248289
C	-1.953834	-1.595731	1.506849
C	-2.037130	2.096019	-1.181406
O	-2.326297	2.959050	-1.977116
O	-1.623413	2.326565	0.068042
C	-1.471411	3.709448	0.447481
H	2.581411	1.844148	2.941446
H	4.840252	-0.190153	-1.001977
H	4.872677	2.570106	2.277842
H	5.968688	1.536750	0.312666
H	0.417535	-1.975245	0.399735
H	0.300941	0.539973	1.742568
H	1.081511	-0.884897	2.424594
H	-4.336703	0.945064	-1.283096
H	-3.913496	-0.513184	-2.169269
H	-1.810311	0.479641	-2.514121
H	-5.643332	-1.032420	0.383534
H	-5.147994	-2.338257	2.448831
H	-2.796774	-2.677311	3.151874
H	-0.949822	-1.750026	1.866022
H	-2.426204	4.228564	0.371470
H	-0.735815	4.192881	-0.194202
H	-1.126492	3.689732	1.477446
C	3.745673	-2.305713	-1.868351
H	3.729234	-3.234223	-2.435384
H	4.501555	-2.370916	-1.084574
H	4.024946	-1.495637	-2.546830

Chloroform CC(-14,170)
SCF Energy: -1223.14114444 A.U.
Free Energy: -1222.819544 A.U.

Atom	Coordinates (Angstroms) X	Y	Z
C	3.077707	1.388800	2.090532
C	4.342454	0.215932	-0.135583
C	4.362716	1.795020	1.716956
C	2.441031	0.401426	1.358374
C	3.069499	-0.192686	0.255141
C	4.978945	1.210620	0.613309
N	2.193142	-1.146202	-0.318380
C	0.846185	-0.989260	0.249392
C	1.078187	-0.205395	1.567034
C	-0.019871	-0.178163	-0.714680
O	0.484635	0.543929	-1.564979
C	2.363407	-2.075996	-1.313415
O	1.394191	-2.716269	-1.714629
N	-1.380702	-0.234641	-0.580975
C	-2.240697	-0.880289	0.346087
C	-3.571545	-0.677985	-0.046574
C	-3.627078	0.117427	-1.323604
C	-2.169460	0.609619	-1.496569
C	-4.617800	-1.196196	0.697942
C	-4.338656	-1.928173	1.855522
C	-3.016958	-2.119538	2.248289
C	-1.953834	-1.595731	1.506849
C	-2.037130	2.096019	-1.181406
O	-2.326297	2.959050	-1.977116
O	-1.623413	2.326565	0.068042
C	-1.471411	3.709448	0.447481
H	2.581411	1.844148	2.941446
H	4.840252	-0.190153	-1.001977
H	4.872677	2.570106	2.277842
H	5.968688	1.536750	0.312666
H	0.417535	-1.975245	0.399735
H	0.300941	0.539973	1.742568
H	1.081511	-0.884897	2.424594
H	-4.336703	0.945064	-1.283096
H	-3.913496	-0.513184	-2.169269
H	-1.810311	0.479641	-2.514121
H	-5.643332	-1.032420	0.383534
H	-5.147994	-2.338257	2.448831
H	-2.796774	-2.677311	3.151874
H	-0.949822	-1.750026	1.866022
H	-2.426204	4.228564	0.371470
H	-0.735815	4.192881	-0.194202
H	-1.126492	3.689732	1.477446
C	3.745673	-2.305713	-1.868351
H	3.729234	-3.234223	-2.435384
H	4.501555	-2.370916	-1.084574
H	4.024946	-1.495637	-2.546830

Chloroform CT(171,-6)
SCF Energy: -1223.14021841 A.U.
Free Energy: -1222.816283 A.U.

Atom	Coordinates (Angstroms) X	Y	Z
C	3.077707	1.388800	2.090532
C	4.342454	0.215932	-0.135583
C	4.362716	1.795020	1.716956
C	2.441031	0.401426	1.358374
C	3.069499	-0.192686	0.255141
C	4.978945	1.210620	0.613309
N	2.193142	-1.146202	-0.318380
C	0.846185	-0.989260	0.249392
C	1.078187	-0.205395	1.567034
C	-0.019871	-0.178163	-0.714680
O	0.484635	0.543929	-1.564979
C	2.363407	-2.075996	-1.313415
O	1.394191	-2.716269	-1.714629
N	-1.380702	-0.234641	-0.580975
C	-2.240697	-0.880289	0.346087
C	-3.571545	-0.677985	-0.046574
C	-3.627078	0.117427	-1.323604
C	-2.169460	0.609619	-1.496569
C	-4.617800	-1.196196	0.697942
C	-4.338656	-1.928173	1.855522
C	-3.016958	-2.119538	2.248289
C	-1.953834	-1.595731	1.506849
C	-2.037130	2.096019	-1.181406
O	-2.326297	2.959050	-1.977116
O	-1.623413	2.326565	0.068042
C	-1.471411	3.709448	0.447481
H	2.581411	1.844148	2.941446
H	4.840252	-0.190153	-1.001977
H	4.872677	2.570106	2.277842
H	5.968688	1.536750	0.312666
H	0.417535	-1.975245	0.399735
H	0.300941	0.539973	1.742568
H	1.081511	-0.884897	2.424594
H	-4.336703	0.945064	-1.283096
H	-3.913496	-0.513184	-2.169269
H	-1.810311	0.479641	-2.514121
H	-5.643332	-1.032420	0.383534
H	-5.147994	-2.338257	2.448831
H	-2.796774	-2.677311	3.151874
H	-0.949822	-1.750026	1.866022
H	-2.426204	4.228564	0.371470
H	-0.735815	4.192881	-0.194202
H	-1.126492	3.689732	1.477446
C	3.745673	-2.305713	-1.868351
H	3.729234	-3.234223	-2.435384
H	4.501555	-2.370916	-1.084574
H	4.024946	-1.495637	-2.546830

Chloroform CT(-30,122)
SCF Energy: -1223.13953476 A.U.
Free Energy: -1222.817311 A.U.

Atom	Coordinates (Angstroms) X	Y	Z
C	3.077707	1.388800	2.090532
C	4.342454	0.215932	-0.135583
C	4.362716	1.795020	1.716956
C	2.441031	0.401426	1.358374
C	3.069499	-0.192686	0.255141
C	4.978945	1.210620	0.613309
N	2.193142	-1.146202	-0.318380
C	0.846185	-0.989260	0.249392
C	1.078187	-0.205395	1.567034
C	-0.019871	-0.178163	-0.714680
O	0.484635	0.543929	-1.564979
C	2.363407	-2.075996	-1.313415
O	1.394191	-2.716269	-1.714629
N	-1.380702	-0.234641	-0.580975
C	-2.240697	-0.880289	0.346087
C	-3.571545	-0.677985	-0.046574
C	-3.627078	0.117427	-1.323604
C	-2.169460	0.609619	-1.496569
C	-4.617800	-1.196196	0.697942
C	-4.338656	-1.928173	1.855522
C	-3.016958	-2.119538	2.248289
C	-1.953834	-1.595731	1.506849
C	-2.037130	2.096019	-1.181406
O	-2.326297	2.959050	-1.977116
O	-1.623413	2.326565	0.068042
C	-1.471411	3.709448	0.447481
H	2.581411	1.844148	2.941446
H	4.840252	-0.190153	-1.001977
H	4.872677	2.570106	2.277842
H	5.968688	1.536750	0.312666
H	0.417535	-1.975245	0.399735
H	0.300941	0.539973	1.742568
H	1.081511	-0.884897	2.424594
H	-4.336703	0.945064	-1.283096
H	-3.913496	-0.513184	-2.169269
H	-1.810311	0.479641	-2.514121
H	-5.643332	-1.032420	0.383534
H	-5.147994	-2.338257	2.448831
H	-2.796774	-2.677311	3.151874
H	-0.949822	-1.750026	1.866022
H	-2.426204	4.228564	0.371470
H	-0.735815	4.192881	-0.194202
H	-1.126492	3.689732	1.477446
C	3.745673	-2.305713	-1.868351
H	3.729234	-3.234223	-2.435384
H	4.501555	-2.370916	-1.084574
H	4.024946	-1.495637	-2.546830

Chloroform CT(146,174)
SCF Energy: -1223.14094744 A.U.
Free Energy: -1222.817036 A.U.

Atom	Coordinates (Angstroms) X	Y	Z
C	3.077707	1.388800	2.090532
C	4.342454	0.215932	-0.135583
C	4.362716	1.795020	1.716956
C	2.441031	0.401426	1.358374
C	3.069499	-0.192686	0.255141
C	4.978945	1.210620	0.613309
N	2.193142	-1.146202	-0.318380
C	0.846185	-0.989260	0.249392
C	1.078187	-0.205395	1.567034
C	-0.019871	-0.178163	-0.714680
O	0.484635	0.543929	-1.564979
C	2.363407	-2.075996	-1.313415
O	1.394191	-2.716269	-1.714629
N	-1.380702	-0.234641	-0.580975
C	-2.240697	-0.880289	0.346087
C	-3.571545	-0.677985	-0.046574
C	-3.627078	0.117427	-1.323604
C	-2.169460	0.609619	-1.496569
C	-4.617800	-1.196196	0.697942
C	-4.338656	-1.928173	1.855522
C	-3.016958	-2.119538	2.248289
C	-1.953834	-1.595731	1.506849
C	-2.037130	2.096019	-1.181406
O	-2.326297	2.959050	-1.977116
O	-1.623413	2.326565	0.068042
C	-1.471411	3.709448	0.447481
H	2.581411	1.844148	2.941446
H	4.840252	-0.190153	-1.001977
H	4.872677	2.570106	2.277842
H	5.968688	1.536750	0.312666
H	0.417535	-1.975245	0.399735
H	0.300941	0.539973	1.742568
H	1.081511	-0.884897	2.424594
H	-4.336703	0.945064	-1.283096
H	-3.913496	-0.513184	-2.169269
H	-1.810311	0.479641	-2.514121
H	-5.643332	-1.032420	0.383534
H	-5.147994	-2.338257	2.448831
H	-2.796774	-2.677311	3.151874
H	-0.949822	-1.750026	1.866022
H	-2.426204	4.228564	0.371470
H	-0.735815	4.192881	-0.194202
H	-1.126492	3.689732	1.477446
C	3.745673	-2.305713	-1.868351
H	3.729234	-3.234223	-2.435384
H	4.501555	-2.370916	-1.084574
H	4.024946	-1.495637	-2.546830

Chloroform CT(-90,-176)
SCF Energy: -1223.13732365 A.U.
Free Energy: -1222.814194 A.U.

Atom	X	Y	Z
C	3.077707	1.388800	2.090532
C	4.342454	0.215932	-0.135583
C	4.362716	1.795020	1.716956
C	2.441031	0.401426	1.358374
C	3.069499	-0.192686	0.255141
C	4.978945	1.210620	0.613309
N	2.193142	-1.146202	-0.318380
C	0.846185	-0.989260	0.249392
C	1.078187	-0.205395	1.567034
C	-0.019871	-0.178163	-0.714680
O	0.484635	0.543929	-1.564979
C	2.363407	-2.075996	-1.313415
O	1.394191	-2.716269	-1.714629
N	-1.380702	-0.234641	-0.580975
C	-2.240697	-0.880289	0.346087
C	-3.571545	-0.677985	-0.046574
C	-3.627078	0.117427	-1.323604
C	-2.169460	0.609619	-1.496569
C	-4.617800	-1.196196	0.697942
C	-4.338656	-1.928173	1.855522
C	-3.016958	-2.119538	2.248289
C	-1.953834	-1.595731	1.506849
C	-2.037130	2.096019	-1.181406
O	-2.326297	2.959050	-1.977116
O	-1.623413	2.326565	0.068042
C	-1.471411	3.709448	0.447481
H	2.581411	1.844148	2.941446
H	4.840252	-0.190153	-1.001977
H	4.872677	2.570106	2.277842
H	5.968688	1.536750	0.312666
H	0.417535	-1.975245	0.399735
H	0.300941	0.539973	1.742568
H	1.081511	-0.884897	2.424594
H	-4.336703	0.945064	-1.283096
H	-3.913496	-0.513184	-2.169269
H	-1.810311	0.479641	-2.514121
H	-5.643332	-1.032420	0.383534
H	-5.147994	-2.338257	2.448831
H	-2.796774	-2.677311	3.151874
H	-0.949822	-1.750026	1.866022
H	-2.426204	4.228564	0.371470
H	-0.735815	4.192881	-0.194202
H	-1.126492	3.689732	1.477446
C	3.745673	-2.305713	-1.868351
H	3.729234	-3.234223	-2.435384
H	4.501555	-2.370916	-1.084574
H	4.024946	-1.495637	-2.546830

Chloroform TC(122,-18)
SCF Energy: -1223.13626547 A.U.
Free Energy: -1222.813633 A.U.

Atom	Coordinates (Angstroms) X	Y	Z
C	3.077707	1.388800	2.090532
C	4.342454	0.215932	-0.135583
C	4.362716	1.795020	1.716956
C	2.441031	0.401426	1.358374
C	3.069499	-0.192686	0.255141
C	4.978945	1.210620	0.613309
N	2.193142	-1.146202	-0.318380
C	0.846185	-0.989260	0.249392
C	1.078187	-0.205395	1.567034
C	-0.019871	-0.178163	-0.714680
O	0.484635	0.543929	-1.564979
C	2.363407	-2.075996	-1.313415
O	1.394191	-2.716269	-1.714629
N	-1.380702	-0.234641	-0.580975
C	-2.240697	-0.880289	0.346087
C	-3.571545	-0.677985	-0.046574
C	-3.627078	0.117427	-1.323604
C	-2.169460	0.609619	-1.496569
C	-4.617800	-1.196196	0.697942
C	-4.338656	-1.928173	1.855522
C	-3.016958	-2.119538	2.248289
C	-1.953834	-1.595731	1.506849
C	-2.037130	2.096019	-1.181406
O	-2.326297	2.959050	-1.977116
O	-1.623413	2.326565	0.068042
C	-1.471411	3.709448	0.447481
H	2.581411	1.844148	2.941446
H	4.840252	-0.190153	-1.001977
H	4.872677	2.570106	2.277842
H	5.968688	1.536750	0.312666
H	0.417535	-1.975245	0.399735
H	0.300941	0.539973	1.742568
H	1.081511	-0.884897	2.424594
H	-4.336703	0.945064	-1.283096
H	-3.913496	-0.513184	-2.169269
H	-1.810311	0.479641	-2.514121
H	-5.643332	-1.032420	0.383534
H	-5.147994	-2.338257	2.448831
H	-2.796774	-2.677311	3.151874
H	-0.949822	-1.750026	1.866022
H	-2.426204	4.228564	0.371470
H	-0.735815	4.192881	-0.194202
H	-1.126492	3.689732	1.477446
C	3.745673	-2.305713	-1.868351
H	3.729234	-3.234223	-2.435384
H	4.501555	-2.370916	-1.084574
H	4.024946	-1.495637	-2.546830

DMSO, TT(-21,-25)
SCF Energy: -1223.14712640 A.U.
Free Energy: -1222.825829 A.U.

Atom	Coordinates (Angstroms) X	Y	Z
C	3.059261	1.372598	2.111050
C	4.338849	0.228510	-0.123294
C	4.344923	1.786652	1.747463
C	2.429246	0.390997	1.364723
C	3.065671	-0.188093	0.258413
C	4.968904	1.216770	0.640016
N	2.192962	-1.135346	-0.333875
C	0.843134	-0.990606	0.231841
C	1.067582	-0.222202	1.560316
C	-0.027176	-0.174229	-0.722397
O	0.474985	0.559682	-1.568082
C	2.380093	-2.062498	-1.324553
O	1.414162	-2.697447	-1.750197
N	-1.385361	-0.234663	-0.589550
C	-2.244181	-0.890742	0.333974
C	-3.575870	-0.669019	-0.043027
C	-3.639671	0.155690	-1.300440
C	-2.175198	0.619766	-1.496845
C	-4.620472	-1.193420	0.700274
C	-4.337052	-1.950927	1.840294
C	-3.013396	-2.161255	2.217873
C	-1.952243	-1.631769	1.477434
C	-2.008727	2.102643	-1.184904
O	-2.219305	2.971352	-2.001294
O	-1.665955	2.324252	0.084816
C	-1.504969	3.702799	0.482160
H	2.557429	1.816538	2.964451
H	4.842565	-0.167197	-0.991115
H	4.849903	2.556633	2.319720
H	5.959387	1.548507	0.348379
H	0.420818	-1.980719	0.371332
H	0.288389	0.518957	1.743741
H	1.069819	-0.912775	2.408245
H	-4.329966	0.996884	-1.221254
H	-3.960798	-0.446218	-2.153926
H	-1.835414	0.478789	-2.519448
H	-5.647006	-1.013774	0.398697
H	-5.144646	-2.365540	2.432757
H	-2.789615	-2.737822	3.108507
H	-0.947313	-1.802087	1.826261
H	-2.443760	4.241025	0.358362
H	-0.724634	4.173455	-0.114413
H	-1.217558	3.668751	1.528884
C	3.771778	-2.300533	-1.849842
H	3.767211	-3.237090	-2.403866
H	4.512693	-2.354628	-1.051529
H	4.065261	-1.501130	-2.534938

DMSO TT(-26,163)
SCF Energy: -1223.14733201 A.U.
Free Energy: -1222.825265 A.U.

Atom	Coordinates (Angstroms) X	Y	Z
C	3.059261	1.372598	2.111050
C	4.338849	0.228510	-0.123294
C	4.344923	1.786652	1.747463
C	2.429246	0.390997	1.364723
C	3.065671	-0.188093	0.258413
C	4.968904	1.216770	0.640016
N	2.192962	-1.135346	-0.333875
C	0.843134	-0.990606	0.231841
C	1.067582	-0.222202	1.560316
C	-0.027176	-0.174229	-0.722397
O	0.474985	0.559682	-1.568082
C	2.380093	-2.062498	-1.324553
O	1.414162	-2.697447	-1.750197
N	-1.385361	-0.234663	-0.589550
C	-2.244181	-0.890742	0.333974
C	-3.575870	-0.669019	-0.043027
C	-3.639671	0.155690	-1.300440
C	-2.175198	0.619766	-1.496845
C	-4.620472	-1.193420	0.700274
C	-4.337052	-1.950927	1.840294
C	-3.013396	-2.161255	2.217873
C	-1.952243	-1.631769	1.477434
C	-2.008727	2.102643	-1.184904
O	-2.219305	2.971352	-2.001294
O	-1.665955	2.324252	0.084816
C	-1.504969	3.702799	0.482160
H	2.557429	1.816538	2.964451
H	4.842565	-0.167197	-0.991115
H	4.849903	2.556633	2.319720
H	5.959387	1.548507	0.348379
H	0.420818	-1.980719	0.371332
H	0.288389	0.518957	1.743741
H	1.069819	-0.912775	2.408245
H	-4.329966	0.996884	-1.221254
H	-3.960798	-0.446218	-2.153926
H	-1.835414	0.478789	-2.519448
H	-5.647006	-1.013774	0.398697
H	-5.144646	-2.365540	2.432757
H	-2.789615	-2.737822	3.108507
H	-0.947313	-1.802087	1.826261
H	-2.443760	4.241025	0.358362
H	-0.724634	4.173455	-0.114413
H	-1.217558	3.668751	1.528884
C	3.771778	-2.300533	-1.849842
H	3.767211	-3.237090	-2.403866
H	4.512693	-2.354628	-1.051529
H	4.065261	-1.501130	-2.534938

DMSO TC(-13,-27)
SCF Energy: -1223.14779392 A.U.
Free Energy: -1222.826054 A.U.

Atom	X	Y	Z
C	3.059261	1.372598	2.111050
C	4.338849	0.228510	-0.123294
C	4.344923	1.786652	1.747463
C	2.429246	0.390997	1.364723
C	3.065671	-0.188093	0.258413
C	4.968904	1.216770	0.640016
N	2.192962	-1.135346	-0.333875
C	0.843134	-0.990606	0.231841
C	1.067582	-0.222202	1.560316
C	-0.027176	-0.174229	-0.722397
O	0.474985	0.559682	-1.568082
C	2.380093	-2.062498	-1.324553
O	1.414162	-2.697447	-1.750197
N	-1.385361	-0.234663	-0.589550
C	-2.244181	-0.890742	0.333974
C	-3.575870	-0.669019	-0.043027
C	-3.639671	0.155690	-1.300440
C	-2.175198	0.619766	-1.496845
C	-4.620472	-1.193420	0.700274
C	-4.337052	-1.950927	1.840294
C	-3.013396	-2.161255	2.217873
C	-1.952243	-1.631769	1.477434
C	-2.008727	2.102643	-1.184904
O	-2.219305	2.971352	-2.001294
O	-1.665955	2.324252	0.084816
C	-1.504969	3.702799	0.482160
H	2.557429	1.816538	2.964451
H	4.842565	-0.167197	-0.991115
H	4.849903	2.556633	2.319720
H	5.959387	1.548507	0.348379
H	0.420818	-1.980719	0.371332
H	0.288389	0.518957	1.743741
H	1.069819	-0.912775	2.408245
H	-4.329966	0.996884	-1.221254
H	-3.960798	-0.446218	-2.153926
H	-1.835414	0.478789	-2.519448
H	-5.647006	-1.013774	0.398697
H	-5.144646	-2.365540	2.432757
H	-2.789615	-2.737822	3.108507
H	-0.947313	-1.802087	1.826261
H	-2.443760	4.241025	0.358362
H	-0.724634	4.173455	-0.114413
H	-1.217558	3.668751	1.528884
C	3.771778	-2.300533	-1.849842
H	3.767211	-3.237090	-2.403866
H	4.512693	-2.354628	-1.051529
H	4.065261	-1.501130	-2.534938

DMSO CT(-27,-39)
SCF Energy: -1223.14750516 A.U.
Free Energy: -1222.826352 A.U.

Atom	Coordinates (Angstroms) X	Y	Z
C	3.059261	1.372598	2.111050
C	4.338849	0.228510	-0.123294
C	4.344923	1.786652	1.747463
C	2.429246	0.390997	1.364723
C	3.065671	-0.188093	0.258413
C	4.968904	1.216770	0.640016
N	2.192962	-1.135346	-0.333875
C	0.843134	-0.990606	0.231841
C	1.067582	-0.222202	1.560316
C	-0.027176	-0.174229	-0.722397
O	0.474985	0.559682	-1.568082
C	2.380093	-2.062498	-1.324553
O	1.414162	-2.697447	-1.750197
N	-1.385361	-0.234663	-0.589550
C	-2.244181	-0.890742	0.333974
C	-3.575870	-0.669019	-0.043027
C	-3.639671	0.155690	-1.300440
C	-2.175198	0.619766	-1.496845
C	-4.620472	-1.193420	0.700274
C	-4.337052	-1.950927	1.840294
C	-3.013396	-2.161255	2.217873
C	-1.952243	-1.631769	1.477434
C	-2.008727	2.102643	-1.184904
O	-2.219305	2.971352	-2.001294
O	-1.665955	2.324252	0.084816
C	-1.504969	3.702799	0.482160
H	2.557429	1.816538	2.964451
H	4.842565	-0.167197	-0.991115
H	4.849903	2.556633	2.319720
H	5.959387	1.548507	0.348379
H	0.420818	-1.980719	0.371332
H	0.288389	0.518957	1.743741
H	1.069819	-0.912775	2.408245
H	-4.329966	0.996884	-1.221254
H	-3.960798	-0.446218	-2.153926
H	-1.835414	0.478789	-2.519448
H	-5.647006	-1.013774	0.398697
H	-5.144646	-2.365540	2.432757
H	-2.789615	-2.737822	3.108507
H	-0.947313	-1.802087	1.826261
H	-2.443760	4.241025	0.358362
H	-0.724634	4.173455	-0.114413
H	-1.217558	3.668751	1.528884
C	3.771778	-2.300533	-1.849842
H	3.767211	-3.237090	-2.403866
H	4.512693	-2.354628	-1.051529
H	4.065261	-1.501130	-2.534938

DMSO TC(-23,163)
SCF Energy: -1223.14786572 A.U.
Free Energy: -1222.827140 A.U.

Atom	Coordinates (Angstroms) X	Y	Z
C	3.059261	1.372598	2.111050
C	4.338849	0.228510	-0.123294
C	4.344923	1.786652	1.747463
C	2.429246	0.390997	1.364723
C	3.065671	-0.188093	0.258413
C	4.968904	1.216770	0.640016
N	2.192962	-1.135346	-0.333875
C	0.843134	-0.990606	0.231841
C	1.067582	-0.222202	1.560316
C	-0.027176	-0.174229	-0.722397
O	0.474985	0.559682	-1.568082
C	2.380093	-2.062498	-1.324553
O	1.414162	-2.697447	-1.750197
N	-1.385361	-0.234663	-0.589550
C	-2.244181	-0.890742	0.333974
C	-3.575870	-0.669019	-0.043027
C	-3.639671	0.155690	-1.300440
C	-2.175198	0.619766	-1.496845
C	-4.620472	-1.193420	0.700274
C	-4.337052	-1.950927	1.840294
C	-3.013396	-2.161255	2.217873
C	-1.952243	-1.631769	1.477434
C	-2.008727	2.102643	-1.184904
O	-2.219305	2.971352	-2.001294
O	-1.665955	2.324252	0.084816
C	-1.504969	3.702799	0.482160
H	2.557429	1.816538	2.964451
H	4.842565	-0.167197	-0.991115
H	4.849903	2.556633	2.319720
H	5.959387	1.548507	0.348379
H	0.420818	-1.980719	0.371332
H	0.288389	0.518957	1.743741
H	1.069819	-0.912775	2.408245
H	-4.329966	0.996884	-1.221254
H	-3.960798	-0.446218	-2.153926
H	-1.835414	0.478789	-2.519448
H	-5.647006	-1.013774	0.398697
H	-5.144646	-2.365540	2.432757
H	-2.789615	-2.737822	3.108507
H	-0.947313	-1.802087	1.826261
H	-2.443760	4.241025	0.358362
H	-0.724634	4.173455	-0.114413
H	-1.217558	3.668751	1.528884
C	3.771778	-2.300533	-1.849842
H	3.767211	-3.237090	-2.403866
H	4.512693	-2.354628	-1.051529
H	4.065261	-1.501130	-2.534938

DMSO CC(-15,-10)
SCF Energy: -1223.14855481 A.U.
Free Energy: -1222.827013 A.U.

Atom	Coordinates (Angstroms) X	Y	Z
C	3.059261	1.372598	2.111050
C	4.338849	0.228510	-0.123294
C	4.344923	1.786652	1.747463
C	2.429246	0.390997	1.364723
C	3.065671	-0.188093	0.258413
C	4.968904	1.216770	0.640016
N	2.192962	-1.135346	-0.333875
C	0.843134	-0.990606	0.231841
C	1.067582	-0.222202	1.560316
C	-0.027176	-0.174229	-0.722397
O	0.474985	0.559682	-1.568082
C	2.380093	-2.062498	-1.324553
O	1.414162	-2.697447	-1.750197
N	-1.385361	-0.234663	-0.589550
C	-2.244181	-0.890742	0.333974
C	-3.575870	-0.669019	-0.043027
C	-3.639671	0.155690	-1.300440
C	-2.175198	0.619766	-1.496845
C	-4.620472	-1.193420	0.700274
C	-4.337052	-1.950927	1.840294
C	-3.013396	-2.161255	2.217873
C	-1.952243	-1.631769	1.477434
C	-2.008727	2.102643	-1.184904
O	-2.219305	2.971352	-2.001294
O	-1.665955	2.324252	0.084816
C	-1.504969	3.702799	0.482160
H	2.557429	1.816538	2.964451
H	4.842565	-0.167197	-0.991115
H	4.849903	2.556633	2.319720
H	5.959387	1.548507	0.348379
H	0.420818	-1.980719	0.371332
H	0.288389	0.518957	1.743741
H	1.069819	-0.912775	2.408245
H	-4.329966	0.996884	-1.221254
H	-3.960798	-0.446218	-2.153926
H	-1.835414	0.478789	-2.519448
H	-5.647006	-1.013774	0.398697
H	-5.144646	-2.365540	2.432757
H	-2.789615	-2.737822	3.108507
H	-0.947313	-1.802087	1.826261
H	-2.443760	4.241025	0.358362
H	-0.724634	4.173455	-0.114413
H	-1.217558	3.668751	1.528884
C	3.771778	-2.300533	-1.849842
H	3.767211	-3.237090	-2.403866
H	4.512693	-2.354628	-1.051529
H	4.065261	-1.501130	-2.534938

DMSO TT(-40,142)
SCF Energy: -1223.14387330 A.U.
Free Energy: -1222.821938 A.U.

Atom	X	Y	Z
C	3.059261	1.372598	2.111050
C	4.338849	0.228510	-0.123294
C	4.344923	1.786652	1.747463
C	2.429246	0.390997	1.364723
C	3.065671	-0.188093	0.258413
C	4.968904	1.216770	0.640016
N	2.192962	-1.135346	-0.333875
C	0.843134	-0.990606	0.231841
C	1.067582	-0.222202	1.560316
C	-0.027176	-0.174229	-0.722397
O	0.474985	0.559682	-1.568082
C	2.380093	-2.062498	-1.324553
O	1.414162	-2.697447	-1.750197
N	-1.385361	-0.234663	-0.589550
C	-2.244181	-0.890742	0.333974
C	-3.575870	-0.669019	-0.043027
C	-3.639671	0.155690	-1.300440
C	-2.175198	0.619766	-1.496845
C	-4.620472	-1.193420	0.700274
C	-4.337052	-1.950927	1.840294
C	-3.013396	-2.161255	2.217873
C	-1.952243	-1.631769	1.477434
C	-2.008727	2.102643	-1.184904
O	-2.219305	2.971352	-2.001294
O	-1.665955	2.324252	0.084816
C	-1.504969	3.702799	0.482160
H	2.557429	1.816538	2.964451
H	4.842565	-0.167197	-0.991115
H	4.849903	2.556633	2.319720
H	5.959387	1.548507	0.348379
H	0.420818	-1.980719	0.371332
H	0.288389	0.518957	1.743741
H	1.069819	-0.912775	2.408245
H	-4.329966	0.996884	-1.221254
H	-3.960798	-0.446218	-2.153926
H	-1.835414	0.478789	-2.519448
H	-5.647006	-1.013774	0.398697
H	-5.144646	-2.365540	2.432757
H	-2.789615	-2.737822	3.108507
H	-0.947313	-1.802087	1.826261
H	-2.443760	4.241025	0.358362
H	-0.724634	4.173455	-0.114413
H	-1.217558	3.668751	1.528884
C	3.771778	-2.300533	-1.849842
H	3.767211	-3.237090	-2.403866
H	4.512693	-2.354628	-1.051529
H	4.065261	-1.501130	-2.534938

DMSO CC(143,173)
SCF Energy: -1223.14900468 A.U.
Free Energy: -1222.825436 A.U.

Atom	X	Y	Z
C	3.059261	1.372598	2.111050
C	4.338849	0.228510	-0.123294
C	4.344923	1.786652	1.747463
C	2.429246	0.390997	1.364723
C	3.065671	-0.188093	0.258413
C	4.968904	1.216770	0.640016
N	2.192962	-1.135346	-0.333875
C	0.843134	-0.990606	0.231841
C	1.067582	-0.222202	1.560316
C	-0.027176	-0.174229	-0.722397
O	0.474985	0.559682	-1.568082
C	2.380093	-2.062498	-1.324553
O	1.414162	-2.697447	-1.750197
N	-1.385361	-0.234663	-0.589550
C	-2.244181	-0.890742	0.333974
C	-3.575870	-0.669019	-0.043027
C	-3.639671	0.155690	-1.300440
C	-2.175198	0.619766	-1.496845
C	-4.620472	-1.193420	0.700274
C	-4.337052	-1.950927	1.840294
C	-3.013396	-2.161255	2.217873
C	-1.952243	-1.631769	1.477434
C	-2.008727	2.102643	-1.184904
O	-2.219305	2.971352	-2.001294
O	-1.665955	2.324252	0.084816
C	-1.504969	3.702799	0.482160
H	2.557429	1.816538	2.964451
H	4.842565	-0.167197	-0.991115
H	4.849903	2.556633	2.319720
H	5.959387	1.548507	0.348379
H	0.420818	-1.980719	0.371332
H	0.288389	0.518957	1.743741
H	1.069819	-0.912775	2.408245
H	-4.329966	0.996884	-1.221254
H	-3.960798	-0.446218	-2.153926
H	-1.835414	0.478789	-2.519448
H	-5.647006	-1.013774	0.398697
H	-5.144646	-2.365540	2.432757
H	-2.789615	-2.737822	3.108507
H	-0.947313	-1.802087	1.826261
H	-2.443760	4.241025	0.358362
H	-0.724634	4.173455	-0.114413
H	-1.217558	3.668751	1.528884
C	3.771778	-2.300533	-1.849842
H	3.767211	-3.237090	-2.403866
H	4.512693	-2.354628	-1.051529
H	4.065261	-1.501130	-2.534938

DMSO CC(150,2)
SCF Energy: -1223.14767623 A.U.
Free Energy: -1222.824258 A.U.

Atom	Coordinates (Angstroms) X	Y	Z
C	3.059261	1.372598	2.111050
C	4.338849	0.228510	-0.123294
C	4.344923	1.786652	1.747463
C	2.429246	0.390997	1.364723
C	3.065671	-0.188093	0.258413
C	4.968904	1.216770	0.640016
N	2.192962	-1.135346	-0.333875
C	0.843134	-0.990606	0.231841
C	1.067582	-0.222202	1.560316
C	-0.027176	-0.174229	-0.722397
O	0.474985	0.559682	-1.568082
C	2.380093	-2.062498	-1.324553
O	1.414162	-2.697447	-1.750197
N	-1.385361	-0.234663	-0.589550
C	-2.244181	-0.890742	0.333974
C	-3.575870	-0.669019	-0.043027
C	-3.639671	0.155690	-1.300440
C	-2.175198	0.619766	-1.496845
C	-4.620472	-1.193420	0.700274
C	-4.337052	-1.950927	1.840294
C	-3.013396	-2.161255	2.217873
C	-1.952243	-1.631769	1.477434
C	-2.008727	2.102643	-1.184904
O	-2.219305	2.971352	-2.001294
O	-1.665955	2.324252	0.084816
C	-1.504969	3.702799	0.482160
H	2.557429	1.816538	2.964451
H	4.842565	-0.167197	-0.991115
H	4.849903	2.556633	2.319720
H	5.959387	1.548507	0.348379
H	0.420818	-1.980719	0.371332
H	0.288389	0.518957	1.743741
H	1.069819	-0.912775	2.408245
H	-4.329966	0.996884	-1.221254
H	-3.960798	-0.446218	-2.153926
H	-1.835414	0.478789	-2.519448
H	-5.647006	-1.013774	0.398697
H	-5.144646	-2.365540	2.432757
H	-2.789615	-2.737822	3.108507
H	-0.947313	-1.802087	1.826261
H	-2.443760	4.241025	0.358362
H	-0.724634	4.173455	-0.114413
H	-1.217558	3.668751	1.528884
C	3.771778	-2.300533	-1.849842
H	3.767211	-3.237090	-2.403866
H	4.512693	-2.354628	-1.051529
H	4.065261	-1.501130	-2.534938

DMSO CC(-15,169)
SCF Energy: -1223.14874899 A.U.
Free Energy: -1222.827235 A.U.

Atom	Coordinates (Angstroms) X	Y	Z
C	3.059261	1.372598	2.111050
C	4.338849	0.228510	-0.123294
C	4.344923	1.786652	1.747463
C	2.429246	0.390997	1.364723
C	3.065671	-0.188093	0.258413
C	4.968904	1.216770	0.640016
N	2.192962	-1.135346	-0.333875
C	0.843134	-0.990606	0.231841
C	1.067582	-0.222202	1.560316
C	-0.027176	-0.174229	-0.722397
O	0.474985	0.559682	-1.568082
C	2.380093	-2.062498	-1.324553
O	1.414162	-2.697447	-1.750197
N	-1.385361	-0.234663	-0.589550
C	-2.244181	-0.890742	0.333974
C	-3.575870	-0.669019	-0.043027
C	-3.639671	0.155690	-1.300440
C	-2.175198	0.619766	-1.496845
C	-4.620472	-1.193420	0.700274
C	-4.337052	-1.950927	1.840294
C	-3.013396	-2.161255	2.217873
C	-1.952243	-1.631769	1.477434
C	-2.008727	2.102643	-1.184904
O	-2.219305	2.971352	-2.001294
O	-1.665955	2.324252	0.084816
C	-1.504969	3.702799	0.482160
H	2.557429	1.816538	2.964451
H	4.842565	-0.167197	-0.991115
H	4.849903	2.556633	2.319720
H	5.959387	1.548507	0.348379
H	0.420818	-1.980719	0.371332
H	0.288389	0.518957	1.743741
H	1.069819	-0.912775	2.408245
H	-4.329966	0.996884	-1.221254
H	-3.960798	-0.446218	-2.153926
H	-1.835414	0.478789	-2.519448
H	-5.647006	-1.013774	0.398697
H	-5.144646	-2.365540	2.432757
H	-2.789615	-2.737822	3.108507
H	-0.947313	-1.802087	1.826261
H	-2.443760	4.241025	0.358362
H	-0.724634	4.173455	-0.114413
H	-1.217558	3.668751	1.528884
C	3.771778	-2.300533	-1.849842
H	3.767211	-3.237090	-2.403866
H	4.512693	-2.354628	-1.051529
H	4.065261	-1.501130	-2.534938

DMSO CT(149,1)

SCF Energy: -1223.14677912 A.U.

Free Energy: -1222.822485 A.U.

Atom	X	Y	Z
C	3.059261	1.372598	2.111050
C	4.338849	0.228510	-0.123294
C	4.344923	1.786652	1.747463
C	2.429246	0.390997	1.364723
C	3.065671	-0.188093	0.258413
C	4.968904	1.216770	0.640016
N	2.192962	-1.135346	-0.333875
C	0.843134	-0.990606	0.231841
C	1.067582	-0.222202	1.560316
C	-0.027176	-0.174229	-0.722397
O	0.474985	0.559682	-1.568082
C	2.380093	-2.062498	-1.324553
O	1.414162	-2.697447	-1.750197
N	-1.385361	-0.234663	-0.589550
C	-2.244181	-0.890742	0.333974
C	-3.575870	-0.669019	-0.043027
C	-3.639671	0.155690	-1.300440
C	-2.175198	0.619766	-1.496845
C	-4.620472	-1.193420	0.700274
C	-4.337052	-1.950927	1.840294
C	-3.013396	-2.161255	2.217873
C	-1.952243	-1.631769	1.477434
C	-2.008727	2.102643	-1.184904
O	-2.219305	2.971352	-2.001294
O	-1.665955	2.324252	0.084816
C	-1.504969	3.702799	0.482160
H	2.557429	1.816538	2.964451
H	4.842565	-0.167197	-0.991115
H	4.849903	2.556633	2.319720
H	5.959387	1.548507	0.348379
H	0.420818	-1.980719	0.371332
H	0.288389	0.518957	1.743741
H	1.069819	-0.912775	2.408245
H	-4.329966	0.996884	-1.221254
H	-3.960798	-0.446218	-2.153926
H	-1.835414	0.478789	-2.519448
H	-5.647006	-1.013774	0.398697
H	-5.144646	-2.365540	2.432757
H	-2.789615	-2.737822	3.108507
H	-0.947313	-1.802087	1.826261
H	-2.443760	4.241025	0.358362
H	-0.724634	4.173455	-0.114413
H	-1.217558	3.668751	1.528884
C	3.771778	-2.300533	-1.849842
H	3.767211	-3.237090	-2.403866
H	4.512693	-2.354628	-1.051529
H	4.065261	-1.501130	-2.534938

DMSO CT(-17,159)
SCF Energy: -1223.14717244 A.U.
Free Energy: -1222.825098 A.U.

Atom	Coordinates (Angstroms) X	Y	Z
C	3.059261	1.372598	2.111050
C	4.338849	0.228510	-0.123294
C	4.344923	1.786652	1.747463
C	2.429246	0.390997	1.364723
C	3.065671	-0.188093	0.258413
C	4.968904	1.216770	0.640016
N	2.192962	-1.135346	-0.333875
C	0.843134	-0.990606	0.231841
C	1.067582	-0.222202	1.560316
C	-0.027176	-0.174229	-0.722397
O	0.474985	0.559682	-1.568082
C	2.380093	-2.062498	-1.324553
O	1.414162	-2.697447	-1.750197
N	-1.385361	-0.234663	-0.589550
C	-2.244181	-0.890742	0.333974
C	-3.575870	-0.669019	-0.043027
C	-3.639671	0.155690	-1.300440
C	-2.175198	0.619766	-1.496845
C	-4.620472	-1.193420	0.700274
C	-4.337052	-1.950927	1.840294
C	-3.013396	-2.161255	2.217873
C	-1.952243	-1.631769	1.477434
C	-2.008727	2.102643	-1.184904
O	-2.219305	2.971352	-2.001294
O	-1.665955	2.324252	0.084816
C	-1.504969	3.702799	0.482160
H	2.557429	1.816538	2.964451
H	4.842565	-0.167197	-0.991115
H	4.849903	2.556633	2.319720
H	5.959387	1.548507	0.348379
H	0.420818	-1.980719	0.371332
H	0.288389	0.518957	1.743741
H	1.069819	-0.912775	2.408245
H	-4.329966	0.996884	-1.221254
H	-3.960798	-0.446218	-2.153926
H	-1.835414	0.478789	-2.519448
H	-5.647006	-1.013774	0.398697
H	-5.144646	-2.365540	2.432757
H	-2.789615	-2.737822	3.108507
H	-0.947313	-1.802087	1.826261
H	-2.443760	4.241025	0.358362
H	-0.724634	4.173455	-0.114413
H	-1.217558	3.668751	1.528884
C	3.771778	-2.300533	-1.849842
H	3.767211	-3.237090	-2.403866
H	4.512693	-2.354628	-1.051529
H	4.065261	-1.501130	-2.534938

DMSO CT(144,173)
SCF Energy: -1223.14804174 A.U.
Free Energy: -1222.823883 A.U.

Atom	Coordinates (Angstroms) X	Y	Z
C	3.059261	1.372598	2.111050
C	4.338849	0.228510	-0.123294
C	4.344923	1.786652	1.747463
C	2.429246	0.390997	1.364723
C	3.065671	-0.188093	0.258413
C	4.968904	1.216770	0.640016
N	2.192962	-1.135346	-0.333875
C	0.843134	-0.990606	0.231841
C	1.067582	-0.222202	1.560316
C	-0.027176	-0.174229	-0.722397
O	0.474985	0.559682	-1.568082
C	2.380093	-2.062498	-1.324553
O	1.414162	-2.697447	-1.750197
N	-1.385361	-0.234663	-0.589550
C	-2.244181	-0.890742	0.333974
C	-3.575870	-0.669019	-0.043027
C	-3.639671	0.155690	-1.300440
C	-2.175198	0.619766	-1.496845
C	-4.620472	-1.193420	0.700274
C	-4.337052	-1.950927	1.840294
C	-3.013396	-2.161255	2.217873
C	-1.952243	-1.631769	1.477434
C	-2.008727	2.102643	-1.184904
O	-2.219305	2.971352	-2.001294
O	-1.665955	2.324252	0.084816
C	-1.504969	3.702799	0.482160
H	2.557429	1.816538	2.964451
H	4.842565	-0.167197	-0.991115
H	4.849903	2.556633	2.319720
H	5.959387	1.548507	0.348379
H	0.420818	-1.980719	0.371332
H	0.288389	0.518957	1.743741
H	1.069819	-0.912775	2.408245
H	-4.329966	0.996884	-1.221254
H	-3.960798	-0.446218	-2.153926
H	-1.835414	0.478789	-2.519448
H	-5.647006	-1.013774	0.398697
H	-5.144646	-2.365540	2.432757
H	-2.789615	-2.737822	3.108507
H	-0.947313	-1.802087	1.826261
H	-2.443760	4.241025	0.358362
H	-0.724634	4.173455	-0.114413
H	-1.217558	3.668751	1.528884
C	3.771778	-2.300533	-1.849842
H	3.767211	-3.237090	-2.403866
H	4.512693	-2.354628	-1.051529
H	4.065261	-1.501130	-2.534938

DMSO TC (-17,159)
SCF Energy: -1223.14717239 A.U.
Free Energy: -1222.825095 A.U.

Atom	Coordinates (Angstroms) X	Y	Z
C	3.059261	1.372598	2.111050
C	4.338849	0.228510	-0.123294
C	4.344923	1.786652	1.747463
C	2.429246	0.390997	1.364723
C	3.065671	-0.188093	0.258413
C	4.968904	1.216770	0.640016
N	2.192962	-1.135346	-0.333875
C	0.843134	-0.990606	0.231841
C	1.067582	-0.222202	1.560316
C	-0.027176	-0.174229	-0.722397
O	0.474985	0.559682	-1.568082
C	2.380093	-2.062498	-1.324553
O	1.414162	-2.697447	-1.750197
N	-1.385361	-0.234663	-0.589550
C	-2.244181	-0.890742	0.333974
C	-3.575870	-0.669019	-0.043027
C	-3.639671	0.155690	-1.300440
C	-2.175198	0.619766	-1.496845
C	-4.620472	-1.193420	0.700274
C	-4.337052	-1.950927	1.840294
C	-3.013396	-2.161255	2.217873
C	-1.952243	-1.631769	1.477434
C	-2.008727	2.102643	-1.184904
O	-2.219305	2.971352	-2.001294
O	-1.665955	2.324252	0.084816
C	-1.504969	3.702799	0.482160
H	2.557429	1.816538	2.964451
H	4.842565	-0.167197	-0.991115
H	4.849903	2.556633	2.319720
H	5.959387	1.548507	0.348379
H	0.420818	-1.980719	0.371332
H	0.288389	0.518957	1.743741
H	1.069819	-0.912775	2.408245
H	-4.329966	0.996884	-1.221254
H	-3.960798	-0.446218	-2.153926
H	-1.835414	0.478789	-2.519448
H	-5.647006	-1.013774	0.398697
H	-5.144646	-2.365540	2.432757
H	-2.789615	-2.737822	3.108507
H	-0.947313	-1.802087	1.826261
H	-2.443760	4.241025	0.358362
H	-0.724634	4.173455	-0.114413
H	-1.217558	3.668751	1.528884
C	3.771778	-2.300533	-1.849842
H	3.767211	-3.237090	-2.403866
H	4.512693	-2.354628	-1.051529
H	4.065261	-1.501130	-2.534938

DMSO CT (123,-28)
SCF Energy: -1223.14182934 A.U.
Free Energy: -1222.819083 A.U.

Atom	Coordinates (Angstroms) X	Y	Z
C	3.059261	1.372598	2.111050
C	4.338849	0.228510	-0.123294
C	4.344923	1.786652	1.747463
C	2.429246	0.390997	1.364723
C	3.065671	-0.188093	0.258413
C	4.968904	1.216770	0.640016
N	2.192962	-1.135346	-0.333875
C	0.843134	-0.990606	0.231841
C	1.067582	-0.222202	1.560316
C	-0.027176	-0.174229	-0.722397
O	0.474985	0.559682	-1.568082
C	2.380093	-2.062498	-1.324553
O	1.414162	-2.697447	-1.750197
N	-1.385361	-0.234663	-0.589550
C	-2.244181	-0.890742	0.333974
C	-3.575870	-0.669019	-0.043027
C	-3.639671	0.155690	-1.300440
C	-2.175198	0.619766	-1.496845
C	-4.620472	-1.193420	0.700274
C	-4.337052	-1.950927	1.840294
C	-3.013396	-2.161255	2.217873
C	-1.952243	-1.631769	1.477434
C	-2.008727	2.102643	-1.184904
O	-2.219305	2.971352	-2.001294
O	-1.665955	2.324252	0.084816
C	-1.504969	3.702799	0.482160
H	2.557429	1.816538	2.964451
H	4.842565	-0.167197	-0.991115
H	4.849903	2.556633	2.319720
H	5.959387	1.548507	0.348379
H	0.420818	-1.980719	0.371332
H	0.288389	0.518957	1.743741
H	1.069819	-0.912775	2.408245
H	-4.329966	0.996884	-1.221254
H	-3.960798	-0.446218	-2.153926
H	-1.835414	0.478789	-2.519448
H	-5.647006	-1.013774	0.398697
H	-5.144646	-2.365540	2.432757
H	-2.789615	-2.737822	3.108507
H	-0.947313	-1.802087	1.826261
H	-2.443760	4.241025	0.358362
H	-0.724634	4.173455	-0.114413
H	-1.217558	3.668751	1.528884
C	3.771778	-2.300533	-1.849842
H	3.767211	-3.237090	-2.403866
H	4.512693	-2.354628	-1.051529
H	4.065261	-1.501130	-2.534938
