# Supplementary figures and images for: A Proline Mimetic for the Design of New Stable Secondary Structures: Solvent-Dependent Amide Bond Isomerization of (S)-Indoline-2-carboxylic Acid Derivatives
Source: J Org Chem. 2021 Jun 3;86(12):7946–54. doi: 10.1021/acs.joc.1c00184 (PMC8456495; doi:10.1021/acs.joc.1c00184)

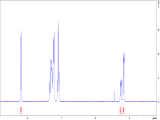

Supplement: Supplementary file 2 — jo1c00184_si_002.zip [file jo1c00184_si_002.zip › FID for pubblication/Ac-Ind-OH HNMR 400 MHz/Ac-Ind-OH in Acetic Acid/pdata/1/thumb.png]

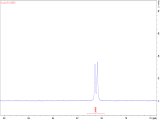

Supplement: Supplementary file 2 — jo1c00184_si_002.zip [file jo1c00184_si_002.zip › FID for pubblication/Ac-Ind-OH HNMR 400 MHz/Ac-Ind-OH in DMSO/pdata/1/thumb.png]

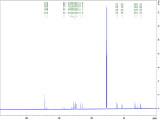

Supplement: Supplementary file 2 — jo1c00184_si_002.zip [file jo1c00184_si_002.zip › FID for pubblication/Ac-Ind-OMe (1) characterization in CDCl3 400 MHz/CNMR (1)/pdata/1/thumb.png]

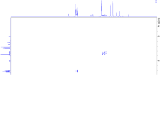

Supplement: Supplementary file 2 — jo1c00184_si_002.zip [file jo1c00184_si_002.zip › FID for pubblication/Ac-Ind-OMe (1) characterization in CDCl3 400 MHz/COSY (1)/pdata/1/thumb.png]

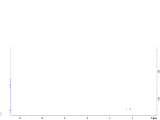

Supplement: Supplementary file 2 — jo1c00184_si_002.zip [file jo1c00184_si_002.zip › FID for pubblication/Ac-Ind-OMe (1) characterization in CDCl3 400 MHz/HMBC (1)/pdata/1/thumb.png]

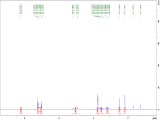

Supplement: Supplementary file 2 — jo1c00184_si_002.zip [file jo1c00184_si_002.zip › FID for pubblication/Ac-Ind-OMe (1) characterization in CDCl3 400 MHz/HNMR (1) 0.1 M/pdata/1/thumb.png]
